# Supplementary material for: An intranasal adjuvanted, recombinant influenza A/H5 vaccine primes against diverse H5N1 clades: a phase I trial
Source: Nat Commun. 2025 Nov 6;16:9321. doi: 10.1038/s41467-025-64686-3 (PMC12592354; doi:10.1038/s41467-025-64686-3)
Supplement: Supplementary file 1 — Supplementary Information [file 41467_2025_64686_MOESM1_ESM.pdf]

SUPPLEMENTARY TABLE

TITLE: An intranasal adjuvanted, recombinant influenza A/H5 vaccine primes against diverse H5N1 clades: a phase I trial

Supplementary Table 1. Serum hemagglutination inhibition assay by group

Vaccine allocation	Group A	Group B	Group C	Group D	Group E
Day 1 and 29	Low-Dose rH5-NE (clade 2.1)	Med-Dose rH5-NE (clade 2.1)	High-Dose rH5-NE (clade 2.1)	Unadjuvanted rH5 (clade 2.1)	Placebo
Day 197	H5N1 IIV (clade 1)	H5N1 IIV (clade 1)	H5N1 IIV (clade 1)	H5N1 IIV (clade 1)	H5N1 IIV (clade 1)
**H5N1 clade 2.1**					
**Day 1-n=**	8	8	8	8	8
GMT (95% CI)	5.5 (4.4-6.7)	5.0 (5.0-5.0)	5.5 (4.4-6.7)	5.0 (5.0-5.0)	5.0 (5.0-5.0)
%seroconversion (95% CI)	0.0 (0.0-36.9)	0.0 (0.0-36.9)	0.0 (0.0-36.9)	0.0 (0.0-36.9)	0.0 (0.0-36.9)
**Day 57-n=**	8	8	8	8	6
GMT (95% CI)	5.5 (4.4-6.7)	5.5 (4.4-6.7)	8.4 (3.8-18.8)	5.0 (5.0-5.0)	5.0 (5.0-5.0)
%seroconversion (95% CI)	0.0 (0.0-36.9)	0.0 (0.0-36.9)	12.5 (0.3-52.7)	0.0 (0.0-36.9)	0.0 (0.0-45.9)
GMFR (95% CI)	1.0 (1.0-1.0)	1.1 (0.9-1.3)	1.5 (0.8-2.9)	1.0 (1.0-1.0)	1.0 (1.0-1.0)
**Day 197-n=**	8	8	8	7	6
GMT (95% CI)	5.0 (5.0-5.0)	5.5 (4.4-6.7)	6.0 (4.0-9.0)	5.0 (5.0-5.0)	5.0 (5.0-5.0)
%seroconversion (95% CI)	0.0 (0.0-36.9)	0.0 (0.0-36.9)	0.0 (0.0-36.9)	0.0 (0.0-41.0)	0.0 (0.0-45.9)
GMFR (95% CI)	0.9 (0.7-1.1)	1.1 (0.9-1.3)	1.1 (0.9-1.3)	1.0 (1.0-1.0)	1.0 (1.0-1.0)
**Day 204-n=**	8	7	8	6	6
GMT (95% CI)	68.3 (25.9-180.1)*	14.9 (8.0-27.8)*	43.6 (18.7-101.5)*	10.0 (6.3-15.8)	15.9 (5.9-42.9)
%seroconversion (95% CI)	75.0 (34.9-96.8)	28.6 (3.7-71.0)	75.0 (34.9-96.8)	0.0 (0.0-45.9)	16.7 (0.4-64.1)
GMFR (95% CI)	12.5 (4.1-38.3)	3.0 (1.6-5.6)	8 (3.7-17.1)	2.0 (1.3-3.2)	3.2 (1.2-8.6)
**Day 225-n=**	8	8	8	7	6
GMT (95% CI)	95.1 (30.2-300.0)*†	18.3 (4.1-82.2)	80.0 (51.6-124.0)*†	5.5 (4.3-7.0)	7.9 (2.4-26.0)
%seroconversion (95% CI)	87.5 (47.3-99.7)	37.5 (8.5-75.5)	100.0 (63.1-100)	0.0 (0.0-41.0)	16.7 (0.4-64.1)
GMFR (95% CI)	17.4 (4.6-66.0)	3.7 (0.8-16.4)	14.7 (10.1-21.3)	1.1 (0.9-1.4)	1.6 (0.5-5.2)
**H5N1 clade 1**					
**Day 1-n=**	8	8	8	8	8
GMT (95% CI)	5.0 (5.0-5.0)	5.5 (4.4-6.7)	5.5 (4.4-6.7)	5.0 (5.0-5.0)	7.7 (2.8-21.5)
%seroconversion (95% CI)	0.0 (0.0-36.9)	0.0 (0.0-36.9)	0.0 (0.0-36.9)	0.0 (0.0-36.9)	0.0 (0.0-36.9)
**Day 57-n=**	8	8	8	8	6
GMT (95% CI)	5.0 (5.0-5.0)	5.0 (5.0-5.0)	5.5 (4.4-6.7)	5.0 (5.0-5.0)	5.0 (5.0-5.0)
%seroconversion (95% CI)	0.0 (0.0-36.9)	0.0 (0.0-36.9)	0.0 (0.0-36.9)	0.0 (0.0-36.9)	0.0 (0.0-45.9)
GMFR (95% CI)	1.0 (1.0-1.0)	0.9 (0.7-1.1)	1.0 (1.0-1.0)	1.0 (1.0-1.0)	1.0 (1.0-1.0)
**Day 197-n=**	8	8	8	7	6
GMT (95% CI)	5.0 (5.0-5.0)	5.5 (4.4-6.7)	5.5 (4.4-6.7)	5.0 (5.0-5.0)	5.0 (5.0-5.0)
%seroconversion (95% CI)	0.0 (0.0-36.9)	0.0 (0.0-36.9)	0.0 (0.0-36.9)	0.0 (0.0-41.0)	0.0 (0.0-45.9)
GMFR (95% CI)	1.0 (1.0-1.0)	1.0 (0.7-1.4)	1.0 (1.0-1.0)	1.0 (1.0-1.0)	1.0 (1.0-1.0)
**Day 204-n=**	8	7	8	6	6
GMT (95% CI)	40.0 (9.4-171.0)*	6.1 (4.5-8.3)	33.6 (10.7-106.1)*	17.0 (3.8-75.9)	7.1 (2.9-17.2)
%seroconversion (95% CI)	62.5 (24.5-91.5)	0.0 (0.0-41.0)	75.0 (34.9-96.8)	33.3 (4.3-77.7)	16.7 (0.4-64.1)
GMFR (95% CI)	8.0 (1.9-34.2)	1.1 (0.9-1.4)	6.2 (1.9-19.6)	3.4 (0.8-15.2)	1.4 (0.6-3.4)
**Day 225-n=**	8	8	8	7	6
GMT (95% CI)	103.8 (52.1-206.5)*†	16.8 (4.4-64.3)	56.6 (22.3-143.3)*†	36.2 (5.4-242.9)	11.2 (3.9-32.8)
%seroconversion (95% CI)	100.0 (63.1-100.0)	37.5 (8.5-75.5)	87.5 (47.3-99.7)	57.1 (18.4-90.1)	33.3 (4.3-77.7)
GMFR (95% CI)	20.7 (10.4-41.3)	3.1 (0.9-11.0)	10.4 (3.9-27.5)	7.2 (1.1-48.6)	2.2 (0.8-6.5)

Sera that were negative by hemagglutination inhibition assay at the initial dilution were assigned a titer of 5. Seroconversion was defined as the percentage of subjects with either a pre-vaccination titer <10 and a post-vaccination titer ≥40 or a pre-vaccination titer ≥10 and a minimum of four-fold rise in post-vaccination titer. Geometric mean fold rise (GMFR) was calculated as the ratio of a GMT at particular timepoint over the baseline value.*Wilcoxon

signed-rank test within group comparison to baseline (Day 1) $p<0.05$. †Wilcoxon signed-rank test within group comparison to Day 57 $p<0.05$.

Supplementary Table 2. Serum microneutralization assay by group

Vaccine allocation	Group A	Group B	Group C	Group D	Group E
Day 1 and 29	Low-Dose rH5-NE (clade 2.1)	Med-Dose rH5-NE (clade 2.1)	High-Dose rH5-NE (clade 2.1)	Unadjuvanted rH5 (clade 2.1)	Placebo
Day 197	H5N1 IIV (clade 1)	H5N1 IIV (clade 1)	H5N1 IIV (clade 1)	H5N1 IIV (clade 1)	H5N1 IIV (clade 1)
**H5N1 clade 2.1**					
**Day 1-n=**	8	8	8	8	8
GMT (95% CI)	10.0 (10.0-10.0)	10.0 (10.0-10.0)	10.0 (10.0-10.0)	10.0 (10.0-10.0)	10.0 (10.0-10.0)
%seroconversion (95% CI)	0.0 (0.0-36.9)	0.0 (0.0-36.9)	0.0 (0.0-36.9)	0.0 (0.0-36.9)	0.0 (0.0-36.9)
**Day 57-n=**	8	8	8	8	6
GMT (95% CI)	13.0 (8.4-20.0)	10.0 (10.0-10.0)	11.9 (7.9-17.9)	10.0 (10.0-10.0)	10.0 (10.0-10.0)
%seroconversion (95% CI)	12.5 (0.3-52.7)	0.0 (0.0-36.9)	12.5 (0.3-52.7)	0.0 (0.0-36.9)	0.0 (0.0-45.9)
GMFR (95% CI)	1.3 (0.8-2.0)	1.0 (1.0-1.0)	1.2 (0.8-1.8)	1.0 (1.0-1.0)	1.0 (1.0-1.0)
**Day 197-n=**	8	8	8	7	6
GMT (95% CI)	10.0 (10.0-10.0)	10.0 (10.0-10.0)	10.0 (10.0-10.0)	10.0 (10.0-10.0)	10.0 (10.0-10.0)
%seroconversion (95% CI)	0.0 (0.0-36.9)	0.0 (0.0-36.9)	0.0 (0.0-36.9)	0.0 (0.0-41)	0.0 (0.0-45.9)
GMFR (95% CI)	1.0 (1.0-1.0)	1.0 (1.0-1.0)	1.0 (1.0-1.0)	1.0 (1.0-1.0)	1.0 (1.0-1.0)
**Day 225-n=**	8	8	8	7	6
GMT (95% CI)	207.5 (40.4-1066.7)*†	146.7 (56.7-380.0)*†	73.4 (35.6-151.1)*†	11.0 (8.7-14.1)	10.0 (10.0-10.0)
%seroconversion (95% CI)	75.0 (34.9-96.8)	87.5 (47.3-99.7)	87.5 (47.3-99.7)	0.0 (0.0-41.0)	0.0 (0.0-45.9)
GMFR (95% CI)	20.8 (4.0-106.7)	14.7 (5.7-38.0)	7.3 (3.6-15.1)	1.1 (0.9-1.4)	1.0 (1.0-1.0)
**H5N1 clade 1**					
**Day 1-n=**	8	8	8	8	8
GMT (95% CI)	10.0 (10.0-10.0)	10.0 (10.0-10.0)	10.0 (10.0-10.0)	10.0 (10.0-10.0)	10.0 (10.0-10.0)
%seroconversion (95% CI)	0.0 (0.0-36.9)	0.0 (0.0-36.9)	0.0 (0.0-36.9)	0.0 (0.0-36.9)	0.0 (0.0-36.9)
**Day 57-n=**	8	8	8	8	6
GMT (95% CI)	10.0 (10.0-10.0)	10.0 (10.0-10.0)	10.0 (10.0-10.0)	10.0 (10.0-10.0)	10.0 (10.0-10.0)
%seroconversion (95% CI)	0.0 (0.0-36.9)	0.0 (0.0-36.9)	0.0 (0.0-36.9)	0.0 (0.0-36.9)	0.0 (0.0-45.9)
GMFR (95% CI)	1.0 (1.0-1.0)	1.0 (1.0-1.0)	1.0 (1.0-1.0)	1.0 (1.0-1.0)	1.0 (1.0-1.0)
**Day 197-n=**	8	8	8	7	6
GMT (95% CI)	13 (8.4-20)	10.0 (10.0-10.0)	10.0 (10.0-10.0)	10.0 (10.0-10.0)	10.0 (10.0-10.0)
%seroconversion (95% CI)	12.5 (0.3-52.7)	0.0 (0.0-36.9)	0.0 (0.0-36.9)	0.0 (0.0-41.0)	0.0 (0.0-45.9)
GMFR (95% CI)	1.3 (0.8-2)	1.0 (1.0-1.0)	1.0 (1.0-1.0)	1.0 (1.0-1.0)	1.0 (1.0-1.0)
**Day 225-n=**	8	8	8	7	6
GMT (95% CI)	174.5 (36.6-832.2)*†	61.7 (25.8-147.6)*†	47.6 (22.6-100.0)*†	36.2 (7.4-177.5)	12.6 (8.7-18.3)
%seroconversion (95% CI)	87.5 (47.3-99.7)	75.0 (34.9-96.8)	75.0 (34.9-96.8)	42.9 (9.9-81.6)	0.0 (0.0-45.9)
GMFR (95% CI)	17.5 (3.7-83.2)	6.2 (2.6-14.8)	4.8 (2.3-10.0)	3.6 (0.7-17.8)	1.3 (0.9-1.8)
**H5N1 clade 2.2, n=**					
**Day 225-n=**	7	7	7	3	0
GMT (95% CI)	97.5 (24.8-384.0)	65.6 (22.0-195.7)	40.0 (15.0-106.5)	10.0 (10.0-10.0)	--
%seroconversion (95% CI)	71.4 (29.0, 96.3)	57.1 (18.4, 90.1)	57.1 (18.4-90.1)	0.0 (0, 70.8)	--
**H5N1 clade 2.2.1, n=**					
**Day 225-n=**	7	7	7	3	0
GMT (95% CI)	144.9 (37.3-562.5)	131.3 (50.3-342.5)	80.0(47.4-135.0)	10.0 (10.0-10.0)	--
%seroconversion (95% CI)	71.4 (29.0, 96.3)	71.4 (29.0, 96.3)	57.1 (18.4-90.1)	66.7 (9.4, 99.2)	--
**H5N1 clade 2.3.4, n=**					
**Day 225-n=**	7	7	7	3	0
GMT (95% CI)	144.9 (37.3-562.5)	131.3 (50.3-342.5)	80.0 (47.4-135.0)	10.0 (10.0-10.0)	--
%seroconversion (95% CI)	85.7 (42.1, 99.6)	100.0 (59.0, 100.0)	100.0 (59.0, 100.0)	0.0 (0.0, 70.8)	--
**H5N1 clade 2.3.4.4b, n=**					
**Day 225-n=**	7	7	7	3	0

GMT (95% CI)	72.5 (40.7-129.0)	72.46 (46.6-112.8)	40.0 (21.1-75.9)	20.0 (20.0-20.0)	--
%seroconversion (95% CI)	100.0 (59.0, 100.0)	100.0 (59.0, 100.0)	85.7 (42.1, 99.6)	0.0 (0.0 70.8)	--

Sera that were negative by microneutralization assay at the initial dilution were assigned a titer of 10. Seroconversion was defined as the percentage of subjects with either a pre-vaccination titer of 10 and a post-vaccination titer ≥40 or a pre-vaccination titer ≥10 and a minimum of four-fold rise in post-vaccination titer. In post hoc analyses, when there was no baseline titer information, we defined seroconversion as a post-vaccination titer ≥40. Geometric mean fold rises (GMFR) were calculated as the ratio of a GMT at particular timepoint over the baseline value.*Wilcoxon signed-rank test within group comparison to baseline (Day 1) $p<0.05$. †Wilcoxon signed-rank test within group comparison to Day 57 $p<0.05$. No statistical comparisons were done for microneutralization results for clade 2.2, clade 2.2.1, clade 2.3.4, or clade 2.3.4.4b.

Supplementary Table 3. H5N1 clade 2.1 serum IgG and IgA and H5 stalk serum IgG by group

Vaccine allocation	Group A	Group B	Group C	Group D	Group E
Day 1 and 29	Low-Dose rH5-NE (clade 2.1)	Med-Dose rH5-NE (clade 2.1)	High-Dose rH5-NE (clade 2.1)	Unadjuvanted rH5 (clade 2.1)	Placebo
Day 197	H5N1 IIV (clade 1)	H5N1 IIV (clade 1)	H5N1 IIV (clade 1)	H5N1 IIV (clade 1)	H5N1 IIV (clade 1)
**H5N1 clade 2.1 IgG**					
**Day 1, n=**	8	8	8	8	8
GMT (95% CI)	9122.5 (5191.7-16029.4)	8163.3 (3556.4-18,737.8)	6216.6 (2780.3-13,900.2)	8630.2 (5323.9-13,989.7)	3901.5 (1763.9-8629.5)
**Day 57, n=**	8	8	8	8	6
GMT (95% CI)	49,976.0 (32,392.7-77,105.0)*	39,017.0 (27,607.0-55,143.9)*	38,849.0 (18,416.5-81,952.1)*	7842.8 (4661.2-13,196.0)	3460.8 (1247.4-9601.8)
%seroconversion (95% CI)	75.0 (34.9-96.8)	37.5 (8.5-75.5)	62.5 (24.5-91.5)	0.0 (0.0-36.9)	0.0 (0.0-45.9)
GMFR (95% CI)	5.5 (3.2-9.4)	4.8 (1.9-11.7)	6.2 (2.1-18.3)	0.9 (0.7-1.1)	0.8 (0.5-1.2)
**Day 197, n=**	8	8	8	7	6
GMT (95% CI)	25,151.0 (13,899.0-45,513.4)*	28,654.0 (16,378.9-50,136.3)*	13,466.0 (7680.1-23,609.5)*	6289.5 (4122.6-9595.5)	3371.6 (1208.7-9405.1)
%seroconversion (95% CI)	12.5 (0.3-52.7)	37.5 (8.5-75.5)	12.5 (0.3-52.7)	0.0 (0.0-41.0)	0.0 (0.0-45.9)
GMFR (95% CI)	2.8 (2.0-3.8)	3.5 (1.6-7.5)	2.2 (1.2-3.8)	0.8 (0.6-1.1)	0.8 (0.6-1.0)
**Day 225, n=**	8	8	8	7	6
GMT (95% CI)	337,623.0 (159,635.5-714,058.3)*†	222,299.0 (99,567.2-49,6316.3)*†	200,487.0 (123,428.7-325,653.4)*†	49,670.0 (30,435.3-81,061.5)*†	24,731.0 (9660.0-63,315.8)*†
%seroconversion (95% CI)	100.0 (63.1-100.0)	87.5 (47.4-99.7)	100.0 (63.1-100.0)	71.4 (29.0-96.3)	50.0 (11.8-88.2)
GMFR (95% CI)	37.0 (13.1-104.6)	27.2 (6.9-107.8)	32.3 (14.2-73.2)	6.4 (3.4-11.9)	5.8 (1.1-31.5)
**H5N1 clade 2.1 IgA**					
**Day 1, n=**	8	8	8	8	8
GMT (95% CI)	138.3 (67.3-284.1)	167.8 (53.3-527.9)	118.6 (41.4-340.2)	177.9 (62.6-505.3)	128.1 (66.9-245.2)
**Day 57, n=**	8	8	8	8	6
GMT (95% CI)	2197.7 (681.3-7088.8)*	1476.2 (367.6-5928.3)*	1204.2 (602.4-2406.9)*	245.3 (93.8-641.3)	123.3 (58.7-258.8)
%seroconversion (95% CI)	75.0 (34.9-96.8)	62.5 (24.5-91.5)	62.5 (24.5-91.5)	0.0 (0.0-36.9)	0.0 (0.0-45.9)
GMFR (95% CI)	15.9 (5.0-50.1)	8.8 (2.9-27.1)	10.1 (2.2-47.9)	1.4 (1.0-1.9)	0.9 (0.7-1.1)
**Day 197, n=**	8	8	8	7	6
GMT (95% CI)	1281.1 (461.2-3558.5)*	823.8 (200.0-3393.1)*	466.6 (210.3-1035.4)*	259.4 (97.5-690.2)	118.0 (53.9-258.3)
%seroconversion (95% CI)	75.0 (34.9-96.8)	50.0 (15.7-84.3)	37.5 (8.5-75.5)	0.0 (0.0-41.0)	0.0 (0.0-45.9)
GMFR (95% CI)	9.3 (3.6-24.0)	4.9 (2.2-10.9)	3.9 (1.2-12.4)	1.5 (1.0-2.3)	0.8 (0.7-1.1)
**Day 225, n=**	8	8	8	7	6
GMT (95% CI)	4900.7 (1343.3-17,878.1)*†	4483.1 (1753.8-11,460.1)*†	2858.2 (1680.1-4862.3)*†	864.7 (299.9-2493.6)*†	423.2 (135.1-1325.6)*†
%seroconversion (95% CI)	100.0 (63.1-100.0)	87.5 (47.4-99.7)	100.0 (63.1-100.0)	85.7 (42.1-99.6)	16.7 (0.4-64.1)
**Phylogenetic group 1 anti-stalk IgG**					
**Day 1, n=**	8	8	8	8	8
GMT (95% CI)	27,456.0 (13499.7-55840.8)	29,966.0 (14886.7-60321.4)	25,005.0 (14811.5-42214.9)	20,420.0 (10569.4-39452.1)	15,706.0 (9107.7-27084.3
**Day 57, n=**	8	8	8	8	6

GMT (95% CI)	71,304.0 (37766.5-134621.9)*	58,522.0 (34440.0-99442.4)	56,628.0 (34033.6-94223.1)*	32,176.0 (19072.5-54282.2)	10,581.0 (3148.2-35559.0)
%seroconversion (95% CI)	12.5 (0.3-52.7)	25.0 (3.2-65.1)	0.0 (0.0-36.9)	12.5 (0.3-52.7)	0.0 (0.0-45.9)
GMFR (95% CI)	2.6 (1.7-3.9)	2.0 (0.7-5.2)	2.3 (1.6-3.1)	1.6 (0.7-3.4)	0.6 (0.2-1.9)
**Day 197, n=**	8	8	8	7	6
GMT (95% CI)	97,078.0 (49792.9-189265.3)*	104,445.0 (61460.1-177492.6)*	33,956.0 (21558.7-53483.5)	31,198.0 (13527.0-71953.0)	16,822.0 (5316.7-53227.6)
%seroconversion (95% CI)	37.5 (8.5-75.5)	37.5 (8.5-75.5)	0.0 (0.0-36.9)	0.0 (0.0-41.0)	0.0 (0.0-45.9)
GMFR (95% CI)	3.5 (2.5-5.0)	3.5 (1.5-8.0)	1.4 (0.7-2.5)	1.6 (0.7-3.9)	0.9 (0.3-3.3)
**Day 225, n=**	8	8	8	7	6
GMT (95% CI)	179,662.0 (101879.0-316829.4)*	156,010.0 (81021.3-300403.1)*	83,481.0 (54574.4-127699.1)*†	73,730.0 (45392.8-119757.8)*	55,851.0 (25441.1-122610.6)*†
%seroconversion (95% CI)	50.0 (15.7-84.3)	62.5 (24.5-91.5)	12.5 (0.3-52.7)	57.1 (18.4-90.1)	16.7 (0.4-64.1)
GMFR (95% CI)	6.5 (1.9-22.2)	5.2 (2.8-9.8)	3.3 (2.7-4.1)	3.9 (1.8-8.2)	3.1 (1.7-5.5)

*Wilcoxon signed-rank test within group comparison to baseline (Day 1) $p<0.05$. †Wilcoxon signed-rank test within group comparison to Day 197 $p<0.05$.

Supplementary Table 4. H5N1 clade 2.1 and clade 1 serum surface plasmon resonance by group

Vaccine allocation	Group A	Group B	Group C	Group D	Group E
Day 1 and 29	Low-Dose rH5-NE (clade 2.1)	Med-Dose rH5-NE (clade 2.1)	High-Dose rH5-NE (clade 2.1)	Unadjuvanted rH5 (clade 2.1)	Placebo
Day 197	H5N1 IIV (clade 1)	H5N1 IIV (clade 1)	H5N1 IIV (clade 1)	H5N1 IIV (clade 1)	H5N1 IIV (clade 1)
**H5N1 clade 2.1**					
**Day 1, n=**	8	8	8	8	8
Mean±SD	6.4±9.6	18.8±16.8	29.2±24.6	19.6±30.0	5.6±9.8
**Day 57, n=**	8	8	8	8	6
Mean±SD	132.9±191.2*	71.8±80.7	135.2±148.4*	64.2±146.7	3.2±4.5
**Day 225, n=**	8	8	8	7	6
Mean±SD	573.4±416.2*†	416.1±339.3*†	350.9±245.6*†	100.9±108.0*†	24.8±25.6
**H5N1 clade 1**					
**Day 1, n=**	8	8	8	8	8
Mean±SD	5.4±7.2	16.1±21.3	14.1±15.5	7.0±7.5	6.6±6.7
**Day 57, n=**	8	8	8	8	6
Mean±SD	46.7±52.5	22.7±21.9	61.8±59.7	22.6±45.6	7.7±6.4
**Day 225, n=**	8	8	8	7	6
Mean±SD	342.3±313.3*†	123.2±102.5*†	178.4±151.4*†	34.8±30.3*†	13.9±11.0

*Wilcoxon signed-rank test within group comparison to baseline (Day 1) p<0.05. †Wilcoxon signed-rank test within group comparison to Day 197 p<0.05.

Supplementary Table 5. Anti-H5 antibodies with ADCC activity by group

rH5 clade 2.1					
**Day 1, n=**	6	7	8	8	6
Mean±SD [RLU]	12205 ±5799.01	41304.71 ±42063.5	62649.62 ±70682.4	58951 ±51188.07	16582 ±19309.68
**Day 57, n=**	6	7	8	8	6
Mean±SD [RLU]	116509.5 ±104854.5*	169474.29 ±140155.4*	229747.5 ±102467.5*	58274.62 ±60914.2	17916.67 ±22420.5
%ADCC seroconversion (95% CI)	50 (11.8-88.1)	57.1 (18.4-90.1)	75 (34.9-96.8)	0 (0-36.9)	0 (0-45.9)
FR: Mean±SD	9.91±7.64	4.38±2.23	12.31±17.18	1±0.38	1.05±0.19
**Day 225, n=**	6	7	8	6	6
Mean±SD [RLU]	284639.5 ±90146.2*	321506.29 ±132808.6*	375434.88 ±58041.2*	224664.5 ±160491.6*	76969.83 ±92758.6*
%ADCC seroconversion (95% CI)	100 (54.0-100.0)	85.7 (42.1-99.6)	75 (34.9-96.8)	33.3 (4.3-77.7)	50 (11.8-88.1)
FR: Mean±SD	28.59±18.45	15.27±15.14	20.53±20.19	3.94±3.61	5.24±5.05

*Wilcoxon signed-rank test within group comparison to baseline (Day 1) p<0.05.

RLU: Relative luminescence units. FR: fold rise

Supplementary Table 6. H5N1 clade 2.1 nasal wash IgG and IgA ratios (H5-specific/total) by group

Vaccine allocation	Group A	Group B	Group C	Group D	Group E
Day 1 and 29	Low-Dose rH5-NE (clade 2.1)	Med-Dose rH5-NE (clade 2.1)	High-Dose rH5-NE (clade 2.1)	Unadjuvanted rH5 (clade 2.1)	Placebo
Day 197	H5N1 IIV (clade 1)	H5N1 IIV (clade 1)	H5N1 IIV (clade 1)	H5N1 IIV (clade 1)	H5N1 IIV (clade 1)
**Clade 2.1 H5 IgG/total IgG**					
**Day 1,** n=	8	8	8	8	8
Median [Q1, Q3]	0.5 [0.3,0.7]	0.4 [0.2,0.7]	0.6 [0.2,1.2]	0.5 [0.3,0.8]	0.2 [0.1,0.3]
**Day 43,** n=	8	8	8	7	6
Median [Q1, Q3]	3.9 [2.3,10.0]*	3.8 [1.8,6.5]*	2.5 [1.3,6.1]*	0.6 [0.3,1.7]	0.2 [0.1,0.3]
**Day 57,** n=	8	8	8	8	6
Median [Q1, Q3]	2.8 [1.7,4.1]*	3.4 [1.7,5.7]*	3.9 [2.0,5.6]*	0.6 [0.4,1.1]	0.3 [0.1,0.4]
**Day 197,** n=	8	8	8	7	6
Median [Q1, Q3]	2.5 [1.7,2.8]*	3.1 [1.6,5.4]*	2.2 [1.5,2.4]*	0.7 [0.4,1.0]	0.3 [0.1,0.5]
**Day 225,** n=	8	7	8	7	6
Median [Q1, Q3]	26.5 [13.1,47.4]*†	21.7 [13.3,43.9]*†	11.0 [7.6,16.7]*†	4.5 [1.9,6.4]*†	2.1 [0.6,3.2]*†
**Clade 2.1 H5 IgA/total IgA**					
**Day 1,** n=	8	8	8	8	8
Median [Q1, Q3]	0.2 [0.1,0.5]	0.2 [0.2,0.3]	0.2 [0.1,0.4]	0.2 [0.2,1.8]	0.5 [0.2,3.4]
**Day 43,** n=	8	8	8	7	6
Median [Q1, Q3]	2.1 [1.1,4.5]*	1.4 [06,3.3]*	0.9 [0.7,1.6]	0.3 [0,0.6]	0.3 [0.1,1.0]
**Day 57,** n=	8	8	8	8	6
Median [Q1, Q3]	2.6 [0.7,5.5]*	1.1 [0.4,2.9]*	0.6 [0.4,3.0]	0.2 [0.1,0.5]	0.4 [0.0,1.6]
**Day 197,** n=	8	8	8	7	6
Median [Q1, Q3]	2.0 [1.0,2.6]*	0.7 [0.2,1.8]*	0.4 [0.2,1.3]	0.3 [0.2,0.5]	0.2 [0.2,0.4]
**Day 225,** n=	8	7	8	7	6
Median [Q1, Q3]	2.0 [0.8,2.7]*	0.8 [0.4,2.7]*†	0.9 [0.6,1.6]†	0.5 [0.2,0.7]	0.4 [0.2,0.8]

*Wilcoxon signed-rank test within group comparison to baseline (Day 1) $p<0.05$. †Wilcoxon signed-rank test within group comparison to Day 197 $p<0.05$.

Supplementary Table 8. Solicited or unsolicited adverse events within 60 minutes and within seven days of intranasal vaccinations by group

Vaccine allocation	Group A	Group B	Group C	Group D	Group E
Day 1 and 29	Low-Dose rH5-NE (clade 2.1)	Med-Dose rH5-NE (clade 2.1)	High-Dose rH5-NE (clade 2.1)	Unadjuvanted rH5 (clade 2.1)	Placebo
**Dose 1**					
Participants, n=	8	8	8	8	8
Participants reporting solicited or unsolicited adverse events within 60 minutes of first intranasal vaccination					
Any events, n (%)	8 (100.0)	7 (87.5)	8 (100.0)	3 (37.5)	3 (37.5)
Any Grade 2 events, n (%)	1 (12.5)	0 (0.0)	1 (12.5)	1 (12.5)	0 (0.0)
Participants reporting solicited or unsolicited adverse events within seven days of first intranasal vaccination (including events within 60 minutes)					
Any events, n (%)	8 (100.0)	7 (87.5)	8 (100.0)	5 (62.5)	6 (75.0)
Any Grade 2 events, n (%)	1 (12.5)	1 (12.5)	3 (37.5)	2 (25.0)	0 (0.0)
**Dose 2**					
Participants, n=	8	8	8	8	7
Participants reporting solicited or unsolicited adverse events within 60 minutes of second intranasal vaccination					
Any events, n (%)	8 (100.0)	8 (100.0)	8 (100.0)	2 (25.0)	3 (42.9)
Any Grade 2 events, n (%)	1 (12.5)	0 (0.0)	0 (0.0)	0 (0.0)	0 (0.0)
Participants reporting solicited or unsolicited adverse events within seven days of second intranasal vaccination (including events within 60 minutes)					
Any events, n (%)	8 (100.0)	8 (100.0)	8 (100.0)	5 (62.5)	5 (62.5)
Any Grade 2 events, n (%)	1 (12.5)	2 (25.0)	0 (0.0)	1 (12.5.0)	0 (0.0)

Adverse event severity grading was based on standardized assessments recommended by the FDA. Event severity was assessed as either Grade 1 (mild), Grade 2 (moderate), Grade 3 (severe), or Grade 4 (life threatening). After the first intranasal vaccination, participants reported the following Grade 2 unsolicited AEs: COVID-19 and arthralgia, each considered unrelated to study product. After the second intranasal vaccination, participants reported the following Grade 2 unsolicited AEs: COVID-19, oral herpes, bronchitis (with onset 23 days after vaccination), anemia, and two participants with urinary tract infections, each considered unrelated to study product. There were no solicited or unsolicited adverse events of severity Grade 3 or higher within seven days of intranasal vaccination.

Supplementary Table 9. Unsolicited adverse events, laboratory abnormalities, and medically attended adverse events after intranasal vaccination by group

Vaccine allocation	Group A	Group B	Group C	Group D	Group E
Day 1 and 29	Low-Dose rH5-NE (clade 2.1)	Med-Dose rH5-NE (clade 2.1)	High-Dose rH5-NE (clade 2.1)	Unadjuvanted rH5 (clade 2.1)	Placebo
Participants reporting unsolicited adverse events within 28 days of any intranasal vaccination					
Participants, n=	8	8	8	8	8
Any events, n (%)	3 (37.5%)	4 (50.0%)	5 (62.5%)	6 (75.0%)	4 (50.0%)
Any Grade 2 events, n (%)	0 (0.0%)	1 (12.5%)	1 (12.5%)	3 (37.5%)	1 (12.5%)
Participants reporting any medically attended adverse events within 28 days of any intranasal vaccination					
Participants, n=	8	8	8	8	8
Any medically attended events, n (%)	0 (0.0%)	1 (12.5%)	1 (12.5%)	2 (25.0%)	0 (0.0%)
Any Grade 2 events, n (%)	0 (0.0%)	1 (12.5%)	0 (0.0%)	2 (25.0%)	0 (0.0%)
Participants with any laboratory abnormality within seven days of first intranasal vaccination					
Participants, n=	8	8	8	8	8
Any laboratory abnormality, n (%)	4 (50.0)	4 (50.0%)	5 (62.5%)	3 (37.5%)	5 (62.5%)
Any Grade 2 or higher laboratory abnormality, n (%)	1 (12.5%)	0 (0.0%)	0 (0.0%)	1 (12.5%)	0 (0.0%)
Participants with any laboratory abnormality within 14 days of second intranasal vaccination					
Participants, n=	8	8	8	8	7
Any laboratory abnormality, n (%)	4 (50.0%)	1 (12.5%)	2 (25.0%)	1 (12.5%)	0 (0.0%)
Any Grade 2 or higher laboratory abnormality, n (%)	0 (0.0%)	0 (0.0%)	0 (0.0%)	0 (0.0%)	0 (0.0%)

Adverse event severity grading was based on standardized assessments recommended by the FDA. Event severity was assessed as either Grade 1 (mild), Grade 2 (moderate), Grade 3 (severe), or Grade 4 (life threatening). There were no unsolicited adverse events or laboratory abnormalities of severity Grade 3 or higher during the time period.

Supplementary Table 10. Unsolicited adverse events

Group	Days post-vaccination #1/#2/#3	AE term (MedDRA preferred term)	Immediate reaction	MAAE	Severity	Relation to vaccine
D	16	COVID-19	No	No	Grade 2	Not Related
A	70/42	Malpositioned teeth	No	Yes	Grade 1	Not Related
D	37/9	COVID-19	No	No	Grade 1	Not Related
E	0	Bradycardia	Yes	No	Grade 1	Not Related
**A**	**0**	**Blood pressure diastolic**	**Yes**	**No**	**Grade 1**	**RELATED**
D	31/3	Urinary tract infection	No	Yes	Grade 2	Not Related
D	88/60	Oral herpes	No	Yes	Grade 2	Not Related
E	40/12	COVID-19	No	No	Grade 2	Not Related
B	19	Hordeolum	No	No	Grade 1	Not Related
D	27	Otitis externa	No	No	Grade 1	Not Related
**A**	**28/0**	**Toothache**	**Yes**	**No**	**Grade 1**	**RELATED**
A	21	Animal bite	No	No	Grade 1	Not Related
**E**	**36/8**	**Cough**	**No**	**No**	**Grade 1**	**RELATED**
D	31/3	Urinary tract infection	No	Yes	Grade 2	Not Related
B	201/173/5	COVID-19	No	No	Grade 2	Not Related
D	8	COVID-19	No	No	Grade 1	Not Related
B	32/4	Upper respiratory tract infection	No	No	Grade 1	Not Related
**A**	**36/8**	**Upper-airway cough**	**No**	**No**	**Grade 1**	**RELATED**
C	19	COVID-19	No	No	Grade 1	Not Related
**C**	**0**	**Sinus pain**	**Yes**	**No**	**Grade 1**	**RELATED**
B	16	COVID-19	No	No	Grade 1	Not Related
C	208/180/12	Hypersensitivity	No	Yes	Grade 2	Not Related
B	51/23	Bronchitis	No	Yes	Grade 2	Not Related
**C**	**28/0**	**Nasal discomfort**	**Yes**	**No**	**Grade 1**	**RELATED**
C	80/52	Influenza	No	Yes	Grade 1	Not Related
E	207/179/11	Toothache	No	Yes	Grade 1	Not Related
C	0	Arthralgia	No	No	Grade 2	Not Related
C	39/8	Dry eye	No	Yes	Grade 1	Not Related
**C**	**28/0**	**Nasal discomfort**	**Yes**	**No**	**Grade 1**	**RELATED**
C	18	COVID-19	No	No	Grade 1	Not Related
C	181/149	Anemia	No	Yes	Grade 2	Not Related
E	41/13	Headache	No	No	Grade 1	Not Related
E	61/33	Pruritus	No	Yes	Grade 1	Not Related

Among all subjects enrolled and vaccinated. Immediate reaction is within 60 minutes of intranasal vaccination or within 30 minutes of intramuscular vaccination. MAAE= medically attended event. All recovered without sequelae. Bold font indicates events deemed related to study vaccine by the blinded investigator.

Supplementary Table 11. Laboratory Abnormalities

Group	Visit number	Clinical safety laboratory test	Value*	Severity	Related to vaccine
E	4	Total bilirubin (with normal ALT)	High	Grade 1	Not related
E	12	Hemoglobin	Low	Grade 1	Not related
A	4	Alkaline phosphatase	Low	Grade 1	Not related
A	7	Alkaline phosphatase	Low	Grade 1	Not related
A	12	ALT	High	Grade 1	Not related
A	12	Alkaline phosphatase	Low	Grade 1	Not related
E	4	Total bilirubin (with normal ALT)	High	Grade 1	Not related
E	12	Alkaline phosphatase	Low	Grade 1	Not related
A	4	Total bilirubin (with normal ALT)	High	Grade 1	Not related
A	7	Total bilirubin (with normal ALT)	High	Grade 1	Not related
B	7	Alkaline phosphatase	Low	Grade 1	Not related
B	12	Alkaline phosphatase	Low	Grade 1	Not related
D	4	Hemoglobin	Low	Grade 1	Not related (related to study procedure)
D	7	Platelet count	High	Grade 1	Not related
**B**	**4**	**WBC count**	**Low**	**Grade 1**	**RELATED**
B	4	Total bilirubin (with normal ALT)	Low	Grade 1	Not related
B	12	WBC count	Low	Grade 1	Not related
B	12	Alkaline phosphatase	Low	Grade 1	Not related
A	4	Hemoglobin	Low	Grade 2	Not related (related to study procedure)
A	7	Hemoglobin	Low	Grade 1	Not related
A	12	Hemoglobin	Low	Grade 3	Not related (related to study procedure)
D	12	Alkaline phosphatase	Low	Grade 1	Not related
A	4	Total bilirubin (with normal ALT)	High	Grade 1	Not related
**D**	**4**	**Platelet count**	**Low**	**Grade 1**	**RELATED**
**D**	**4**	**ALT**	**High**	**Grade 2**	**RELATED**
D	12	Alkaline phosphatase	Low	Grade 1	Not related
B	4	WBC count	Low	Grade 1	Not related
A	7	Hemoglobin	Low	Grade 1	Not related (related to study procedure)
A	7	Alkaline phosphatase	Low	Grade 1	Not related
A	12	Hemoglobin	Low	Grade 1	Not related (related to study procedure)
D	4	Hemoglobin	Low	Grade 1	Not related (related to study procedure)
**D**	**4**	**ALT**	**High**	**Grade 1**	**RELATED**
**C**	**4**	**WBC count**	**Low**	**Grade 1**	**RELATED**
C	7	Alkaline phosphatase	Low	Grade 1	Not related
C	12	Alkaline phosphatase	Low	Grade 1	Not related
B	4	Platelet count	High	Grade 1	Not related
C	12	Alkaline phosphatase	Low	Grade 1	Not related
B	12	Hemoglobin	Low	Grade 1	Not related
**B**	**4**	**Alkaline phosphatase**	**High**	**Grade 1**	**RELATED**
C	4	Alkaline phosphatase	Low	Grade 1	Not related
C	12	Alkaline phosphatase	Low	Grade 1	Not related
E	4	Alkaline phosphatase	Low	Grade 1	Not related
**E**	**4**	**WBC count**	**Low**	**Grade 1**	**RELATED**
C	4	Alkaline phosphatase	Low	Grade 1	Not related
C	4	Alkaline phosphatase	Low	Grade 1	Not related
C	12	Alkaline phosphatase	Low	Grade 1	Not related
C	4	Hemoglobin	Low	Grade 1	Not related
C	7	Hemoglobin	Low	Grade 1	Not related
C	12	Hemoglobin	Low	Grade 3	Not related (related to study procedure)
**E**	**4**	**Alkaline phosphatase**	**Low**	**Grade 1**	**RELATED**

*Relative to reference range. Bold font indicates events deemed related to study vaccine by the blinded investigator. Low hemoglobin values related to study procedures were attributed to study phlebotomy by the blinded investigator.

Supplementary Table 12. Intramuscular H5N1 IIV safety endpoints by group

Vaccine allocation	Group A	Group B	Group C	Group D	Group E
Day 1 and 29	Low-Dose rH5-NE (clade 2.1)	Med-Dose rH5-NE (clade 2.1)	High-Dose rH5-NE (clade 2.1)	Unadjuvanted rH5 (clade 2.1)	Placebo
Day 197	H5N1 IIV (clade 1)	H5N1 IIV (clade 1)	H5N1 IIV (clade 1)	H5N1 IIV (clade 1)	H5N1 IIV (clade 1)
Participants, n=	8	8	8	7	6
Participants reporting solicited or unsolicited adverse events within 30 minutes of intramuscular vaccination,					
Any events, n (%)	1 (12.5%)	3 (37.5%)	5 (62.5%)	2 (28.6%)	2 (33.3%)
Any Grade 2 events, n (%)	1 (12.5%)	1 (12.5%)	0 (0.0%)	1 (14.3%)	0 (0.0%)
Participants reporting solicited or unsolicited adverse events within seven days of intramuscular vaccination (including events within 30 minutes)					
Any events, n (%)	4 (50.0%)	5 (62.5%)	7 (87.5%)	4 (57.1%)	4 (66.7%)
Any Grade 2, n (%)	1 (12.5%)	2 (25.0%)	0 (0.0%)	1 (14.3%)	0 (0.0%)
Participants reporting unsolicited adverse events within 28 days of intramuscular vaccination					
Any events, n (%)	0 (0.0%)	1 (12.5%)	1 (12.5%)	0 (0.0%)	1 (16.7%)
Any Grade 2 or higher events, n (%)	0 (0.0%)	1 (12.5%)	1 (12.5%)	0 (0.0%)	0 (0.0%)
Participants with medically attended adverse events within 28 days of intramuscular vaccination					
Any medically attended events, n (%)	0 (0.0%)	0 (0.0%)	1 (12.5%)	0 (0.0%)	1 (16.7)%)
Any Grade 2 events, n (%)	0 (0.0%)	0 (0.0%)	1 (12.5%)	0 (0.0%)	0 (0.0%)
Participants with laboratory abnormalities within seven days of intramuscular vaccination					
Any laboratory abnormality, n (%)	3 (37.5%)	3 (37.5%)	5 (62.5%)	2 (28.6%)	2 (33.3%)
Any Grade 2 or higher laboratory abnormality, n (%)	1 (12.5%)	0 (0.0%)	1 (12.5%)	0 (0.0%)	0 (0.0%)

Adverse event severity grading was based on standardized assessments recommended by the FDA. Event severity was assessed as either Grade 1 (mild), Grade 2 (moderate), Grade 3 (severe), or Grade 4 (life threatening). There were no solicited or unsolicited adverse events of severity Grade 3 or higher within seven days of intramuscular vaccination, no unsolicited adverse events or medically attended events of severity Grade 3 or higher within 28 days of intramuscular vaccination, and two laboratory abnormalities of severity Grade 3 or higher within seven days of intramuscular vaccination.

Supplementary Table 13. Potentially immune-mediated medical conditions, new onset chronic medical conditions, and serious adverse events by group

Vaccine allocation	Group A	Group B	Group C	Group D	Group E
Day 1 and 29	Low-Dose rH5-NE (clade 2.1)	Med-Dose rH5-NE (clade 2.1)	High-Dose rH5-NE (clade 2.1)	Unadjuvanted rH5 (clade 2.1)	Placebo
Day 197	H5N1 IIV (clade 1)	H5N1 IIV (clade 1)	H5N1 IIV (clade 1)	H5N1 IIV (clade 1)	H5N1 IIV (clade 1)
Participants, n=	8	8	8	8	8
Participants with potential immune-mediated medical conditions, n (%)	0 (0.0%)	0 (0.0%)	0 (0.0%)	0 (0.0%)	0 (0.0%)
Participants with new onset chronic medical conditions, n (%)	0 (0.0%)	0 (0.0%)	0 (0.0%)	0 (0.0%)	0 (0.0%)
Participants with serious adverse events, n (%)	0 (0.0%)	0 (0.0%)	0 (0.0%)	0 (0.0%)	0 (0.0%)

Potential immune-mediated medical conditions, new onset chronic medical conditions, and serious adverse events were monitored from enrollment through the last study visit (Day 393).

Supplementary Table 14. Antibodies used in flow cytometry

Target	Fluorochrome	Provider	Cat#	Clone	Lot#
CD107a	FITC	BD Biosciences	555800	H4A3	931781
CD62L	PE:	eBiosciences	12-0629-42	DREG	2504394
CD69	ECD:	Beckman Coulter	6607110	TP1.55.3	7620108F
CD4	PerCP-Cy5.5:	BD Biosciences	552838	L200	2199958
IFN-g	PE-Cy7:	BD Biosciences	557643	B27	3089581
CD137	BV421	BioLegend	309820	4-1BB	B368560
CD19	BV570	BioLegend	302236	HIB19	B386458
CD56	BV570	BioLegend	318330	HCD56	B355511
IL-17A	BV605:	BioLegend	512326	BL168	B376461
CD3	BV650:	BD Biosciences	563999	SK7	2320659
TNF-a	BV711:	BioLegend	502940	MAb11	B366939
CD154	BV785:	BioLegend	310842	24-31	B337743
IL-2	Ax647	BioLegend	500315	MQ1-17H12	B380796
CD8	A700:	BD Biosciences	557945	RPA-T8	3101625
CD45RA	APC-H7:	BD Biosciences	560674	HI100	3115564
CD28/CD49d	N/A	BD Biosciences	347690	L293/L25	B293922

Supplementary Figure 1. CD4 T cell responses to rH5 (clade 2.1) antigen

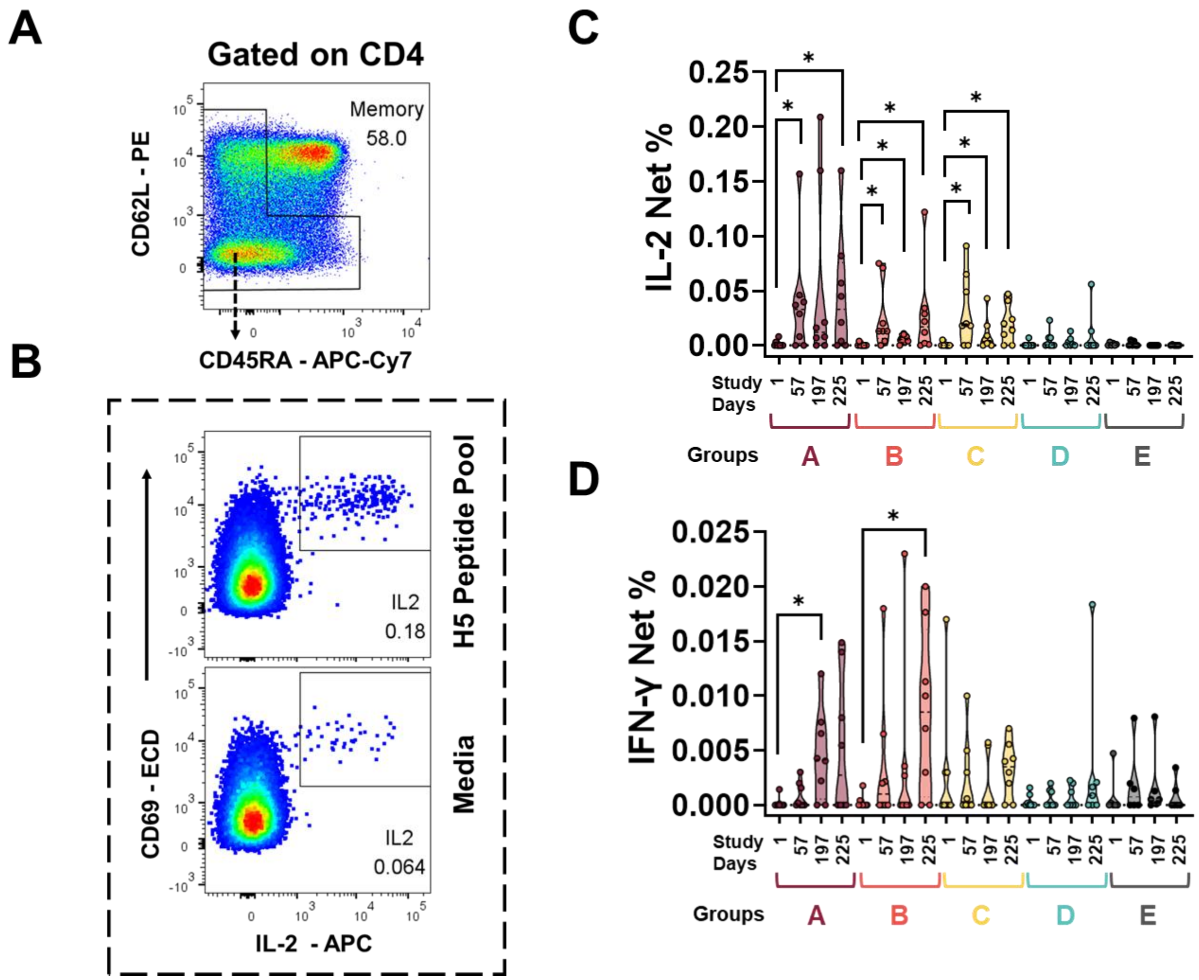

CD4 T cell Responses. In panel **A**, we show the basic gating strategy used to identify memory CD4 T cells using CD54RA and CD62L. Memory CD4 T cells were defined as CD4+ CD62L low/high and CD45RA low/high, excluding double positive CD62+CD45RA+ cells. In **B**, we show a representative example of activated (CD69+) cells producing IL-2 upon stimulation with media (mock stimulation) or the rH5 (clade 2.1) antigen. The data reported in the manuscript is the Net% (peptide-pool minus media). In the example the net percentage is 0.116. In **C**, we show the frequency of memory CD4 T cells producing IL-2 (net %) upon ex-vivo stimulation with rH5 (clade 2.1) antigen. The data is displayed in Violin plots. Panel **D** shows similar data but from IFN-γ producing cells. Data from volunteers vaccinated with the low-dose (Group A), middle-dose (Group B) and high-dose (Group C) of rH5-NE are shown in maroon, orange and yellow. Controls, including the unadjuvanted rH5 (Group D) and placebo (Group D), are shown in cyan and gray colors. Statistics from panels **C-D** are derived from signed-rank tests. *p<0.05.

A Phase 1 Randomized, Double-Blind, Active- and Placebo-Controlled, Dose-Range, Safety, Tolerability, and Immunogenicity Study of Two Doses of Intranasal Recombinant H5 Influenza Vaccine with and without Nanoemulsion Adjuvant Followed by One Boosting Dose of Intramuscular Influenza A (H5N1) Vaccine in Healthy Adult Volunteers

DMID Protocol Number: 20-0036

FDA Submission Tracking Number (STN): IND 27665

DMID Funding Mechanism: NIH grant U01AI148081

IND Sponsor: BlueWillow Biologics, Ann Arbor, MI, USA

Lead Principal Investigator: Justin Ortiz, MD, MS, FACP, FCCP

Draft or Version Number: 11.0

Day Month Year
29 May 2024

STATEMENT OF ASSURANCE

Each Institution will hold a current Federal Wide Assurance (FWA) issued by the Office of Human Research Protections (OHRP) for federally-funded human subjects research. Each FWA will designate at least one Institutional Review Board (IRB)/Independent Ethics Committee (IEC) registered with OHRP, for which the research will be reviewed and approved by the IRB/IEC and will be subject to continuing review [45 CFR 46.103(b)]. The IRB/IEC designated under an FWA may include an institution's IRB/IEC, an independent IRB/IEC, or an IRB/IEC of another institution after establishing a written agreement with that other institution.

STATEMENT OF COMPLIANCE

The study trial will be carried out in accordance with Good Clinical Practice (GCP) and as required by the following:

United States Code of Federal Regulations (CFR) 45 CFR Part 46: Protection of Human Subjects

Food and Drug Administration (FDA) Regulations, as applicable: 21 CFR Part 50 (Protection of Human Subjects), 21 CFR Part 54 (Financial Disclosure by Clinical Investigators), 21 CFR Part 56 (Institutional Review Boards), 21 CFR Part 11, and 21 CFR Part 312 (Investigational New Drug Application), 21 CFR 812 (Investigational Device Exemptions)

International Conference on Harmonisation: Good Clinical Practice (ICH E6); 62 Federal Register 25691 (1997); and future revisions

Belmont Report: Ethical Principles and Guidelines for the Protection of Human Subjects of Research, Report of the National Commission for the Protection of Human Subjects of Biomedical and Behavioral Research

National Institutes of Health (NIH) Office of Extramural Research, Research Involving Human Subjects, as applicable

National Institute of Allergy and Infectious Diseases (NIAID) Clinical Terms of Award, as applicable

Applicable Federal, State, and Local Regulations and Guidance

SIGNATURE PAGE

The signature below provides the necessary assurance that this trial will be conducted according to all stipulations of the protocol, including all statements regarding confidentiality, and according to local legal and regulatory requirements and applicable US federal regulations and ICH E6 Good Clinical Practice (GCP) guidelines.

I agree to conduct the study in compliance with GCP and applicable regulatory requirements.

I agree to conduct the study in accordance with the current protocol and will not make changes to the protocol without obtaining the sponsor's approval and IRB/IEC approval, except when necessary to protect the safety, rights, or welfare of subjects.

Site Investigator Signature:

Signed: ________________________________ Date: ______________

Justin Ortiz, MD, MS, FACP, FCCP
Principal Investigator
Professor of Medicine
Center for Vaccine Development
University of Maryland School of Medicine

TABLE OF CONTENTS

STATEMENT OF ASSURANCE 2
STATEMENT OF COMPLIANCE 3
SIGNATURE PAGE 4
TABLE OF CONTENTS 5
LIST OF TABLES 10
LIST OF ABBREVIATIONS 11
PROTOCOL SUMMARY 14
1 KEY ROLES 18
2 BACKGROUND AND SCIENTIFIC RATIONALE 20
2.1 Background 20
2.1.1 Investigational Product 20
2.1.2 Influenza Disease 20
2.1.3 Current Seasonal Influenza Vaccines 21
2.1.4 Licensed H5N1 Vaccines 22
2.1.5 Need for New Vaccines 22
2.2 Scientific Rationale 24
2.2.1 Purpose of Study 24
2.2.2 Study Population 24
2.3 Potential Risks and Benefits 25
2.3.1 Potential Risks 25
2.3.2 Potential Benefits 26
3 STUDY DESIGN, OBJECTIVES AND ENDPOINTS OR OUTCOME MEASURES 27
3.1 Study Objectives 28
3.1.1 Primary 29
3.1.2 Secondary 29
3.1.3 Exploratory 29
3.2 Study Endpoints or Outcome Measures 30
3.2.1 Primary Endpoints 30
3.2.2 Secondary Endpoints 31
3.2.3 Exploratory Endpoints 32

4 STUDY INTERVENTION/INVESTIGATIONAL PRODUCT 35
4.1 Study Product Description 35
4.1.1 Formulation, Packaging, and Labeling 35
4.1.2 Product Storage and Stability 37
4.2 Acquisition/Distribution 37
4.3 Dosage/Regimen, Preparation, Dispensing and Administration of Study Intervention/Investigational Product 37
4.3.1 Intranasal Administration Procedure Using Eppendorf Pipette 38
4.3.2 Intramuscular Administration of H5N1 IIV Vaccine 39
4.4 Accountability Procedures for the Study Intervention/Investigational Product(s) 39
5 SELECTION OF SUBJECTS AND STUDY ENROLLMENT AND WITHDRAWAL 40
5.1 Eligibility Criteria 40
5.1.1 Subject Inclusion Criteria 40
5.1.2 Subject Exclusion Criteria 41
5.2 Withdrawal from the Study, Discontinuation of Study Product, or Study Termination 42
5.2.1 Withdrawal from the Study or Discontinuation of the Study Product 42
5.2.2 Subject Replacement 43
5.2.3 Study Termination 43
6 STUDY PROCEDURES 44
6.1 Screening (-45 to -3 days prior to randomization) 44
6.1.1 Visit 1: Screening 44
6.2 Baseline, Randomization and Dosing (Day 1) 44
6.2.1 Visit 2: Baseline (pre-dose on Day 1) 44
6.2.2 Visit 2: Randomization and Dosing (Day 1) 45
6.3 Planned Study Visits 45
6.3.1 Visit 3: Safety Call (Day 4 ± 1 day) 45
6.3.2 Visit 4: Safety (Day 8 ± 2 days) 46
6.3.3 Visit 5: Dose #2 (Day 29 ± 4 days) 46
6.3.4 Visit 6: Safety Call (Day 32 ± 2 day) 47
6.3.5 Visit 7: Safety (Day 43 ± 2 days) 47
6.3.6 Visit 8: Safety (Day 57 ± 4 days) 48

6.3.7 Visit 9: Safety Call (Day 90 ± 7 days) 48
6.3.8 Visit 10: H5N1 IIV Boost Vaccine (Day 197 ± 7 days) 48
6.3.9 Visit 11: Safety Call (Day 200 ± 2 days) 49
6.3.10 Visit 12: Safety (Day 204 ± 2 days) 49
6.3.11 Visit 13: Safety (Day 225 ± 7 days) 50
6.3.12 Visit 14: End of Study Call (Day 393 ± 7 days) 50
6.3.13 Early Termination Visit 51
6.3.14 Use of left-over specimens after conduct of specified study assays 51
6.3.15 Collection of specimens for future use 51
6.4 Unscheduled Study Visits 51
6.5 Protocol Deviations 51
7 DESCRIPTION OF CLINICAL AND LABORATORY EVALUATIONS 53
7.1 Clinical Evaluations 53
7.1.1 Clinical Evaluations and Research Procedures 53
7.1.2 Prior and Concomitant Medications/Treatments 55
7.1.3 Assessment of Subject Compliance with Study Intervention/Investigational Product/Investigational Device 55
7.2 Laboratory Evaluations 55
7.2.1 Clinical Laboratory Evaluations 55
7.2.2 Research Assays 56
8 ASSESSMENT OF SAFETY 58
8.1 Assessing and Recording Safety Parameters 58
8.1.1 Adverse Events (AEs) 58
8.1.2 Reactogenicity 60
8.1.3 Serious Adverse Events (SAEs) 60
8.2 Specification of Safety Parameters 61
8.2.1 Solicited Events 61
8.2.2 Unsolicited Events 61
8.2.3 New-Onset Chronic Medical Conditions (NOCMCs) 62
8.2.4 Medically-Attended Adverse Events (MAAEs) 62
8.2.5 Potentially Immune-Mediated Medical Conditions (PIMMCs) 62
8.3 Reporting Procedures 62

8.3.1 Reporting Serious Adverse Events 62
8.3.2 Regulatory Reporting for Studies Not Conducted Under DMID Sponsored IND 63
8.3.3 Reporting of Pregnancy 63
8.4 Type and Duration of Follow-up of Subjects after Adverse Events....63
8.5 Procedures to be Followed in the Event of Abnormal Laboratory Test Values or Abnormal Clinical Findings....64
8.6 Halting Rules 64
8.6.1 Study Halting Criteria 64
8.6.2 Individual Halting Criteria 64
8.7 Safety Oversight 65
8.7.1 Safety Monitoring Committee (SMC) 65
9 HUMAN SUBJECTS PROTECTION 67
9.1 Institutional Review Board/Independent Ethics Committee 67
9.2 Informed Consent Process 67
9.2.1 Exclusion of Women, Minorities, and Children (Special Populations)....69
9.3 Subject Confidentiality 70
9.4 Certificate of Confidentiality 70
9.5 Costs, Subject Compensation, and Research Related Injuries 71
10 STATISTICAL CONSIDERATIONS 72
10.1 Study Hypotheses 72
10.2 Sample Size Considerations 72
10.3 Treatment Assignment Procedures 72
10.3.1 Randomization Procedures 72
10.3.2 Masking Procedures 72
10.4 Planned Interim Analyses 73
10.4.1 Sentinel Subject Safety Review 73
10.4.2 Interim Safety Review 73
10.4.3 Immunogenicity Analysis 73
10.4.4 Analysis Plan 73
11 SOURCE DOCUMENTS AND ACCESS TO SOURCE DATA/DOCUMENTS....75
12 QUALITY CONTROL AND QUALITY ASSURANCE 76

13 DATA HANDLING AND RECORD KEEPING 77
13.1 Data Management Responsibilities 77
13.2 Data Coordinating Center/Biostatistician Responsibilities 77
13.3 Data Capture Methods 77
13.4 Study Records Retention 77
14 CLINICAL MONITORING 79
15 PUBLICATION POLICY 80
16 LITERATURE REFERENCES....81
17 APPENDICES 83
Appendix A. Schedule of Events....84
Appendix B. Toxicity Tables 87
Appendix C. Targeted Neurologic/Otorhinoscopic/Respiratory Assessments 91
Appendix D. Reactogenicity Assessments (post-IN vaccination)....93
Appendix E. Reactogenicity Assessment (post-IM injection) 94
Appendix F. Potentially Immune-Mediated Medical Conditions (PIMMCs) 95
Appendix G. Procedure Volumes....98

LIST OF TABLES

Table 1. Study Treatment Arms....17
Table 2. Sentinel Subjects and Dose Escalation Schedule 28
Table 3. BW-1014 Vaccine Drug Product, Components and Suppliers....36
Table 4. rH5 Control....36
Table 5. Placebo....37
Table 6. Summary of Treatments, Dosages, Routes and Timing of Administration....38

LIST OF ABBREVIATIONS

˚C	degrees Celsius or Centigrade
≤	less than or equal to
µg	microgram
µL	microliter
µm	micrometer
AE	Adverse Event/Adverse Experience
bar	metric unit of pressure
BARDA	Biomedical Advanced Research and Development Authority
BW	BlueWillow Biologics
BW-1014	BlueWillow adjuvanted H5N1 vaccine
CBER	Center for Biologic Evaluation and Research
CDC	Centers for Disease Control and Prevention
CFR	Code of Federal Regulations
cGMP	Current Good Manufacturing Practice(s)
CI	Confidence Interval
CIOMS	Council for International Organizations of Medical Sciences
CITI	Collaborative Institutional Training Initiative
cm	centimeter
CMP	Clinical Monitoring Plan
CPC	cetylpyridinium chloride
CRF	Case Report Form
CRO	Contract Research Organization
CSR	Clinical Study Report
CTRIC	Clinical and Translational Research Informatics Center
CVD	Center for Vaccine Development
DHHS	Department of Health and Human Services
DLS	dynamic light scattering
DMID	Division of Microbiology and Infectious Diseases, NIAID, NIH, DHHS
eCRF	electronic case report form
EDC	electronic data capture
EDR	extemporaneous dispensing record
ELISA	enzyme-linked immunosorbent assay
ELISpot	enzyme-linked immunospot
FDA	Food and Drug Administration
FDAAA	Food and Drug Administration Amendments Act
FhCMB	Fraunhofer USA Center for Molecular Biology
FISMA	Federal Information Security Management Act FISMA
FWA	Federal Wide Assurance
g	gram(s)

GCP	Good Clinical Practice(s)
GLP	Good Laboratory Practice(s)
GMP	Good Manufacturing Practice(s)
GMT	geometric mean titer
h	hour(s)
H5N1 IIV	Sanofi Pasteur Inactivated Influenza Virus Vaccine, H5N1
HA	Hemagglutinin glycoprotein
HCV	Hepatitis C Virus
HHS	Health and Human Services
HI	hemagglutination inhibition
HIPAA	Health Insurance Portability and Accountability Act
HIV-1	Human Immunodeficiency Virus Type 1
HIV-2	Human Immunodeficiency Virus Type 2
IB	Investigator’s Brochure
ICF	Informed Consent Form
ICH	International Conference on Harmonisation
IEC	Independent ethics committee
IFN-ɤ	interferon gamma
IgA	immunoglobulin a
IgG	immunoglobulin g
IL	interleukin
IM	intramuscular
IN	intranasal
IND	Investigational New Drug application
IRB	Institutional Review Board
JAMA	Journal of the American Medical Association
L	liter
LAIV	live-attenuated influenza vaccine
MAAE	medically attended adverse events
MedDRA	Medical Dictionary for Regulatory Activities
mL	milliliter(s)
mM	millimolar
MOP	Manual of Procedures
N	Number (typically refers to subjects)
NALT	nasopharynx-associated lymphoid tissue
NEJM	New England Journal of Medicine
NIAID	National Institute of Allergy and Infectious Diseases, NIH, DHHS
NIH	National Institutes of Health
NOCMC	new onset chronic medical condition
NPIVS	National Pre–Pandemic Influenza Vaccine Stockpile
OHRP	Office for Human Research Protections

OHSR	Office for Human Subjects Research
PBMC	peripheral blood mononuclear cell
pH	potential of Hydrogen - a measure of acidity or alkalinity of water-soluble substances
PHI	Protected Health Information
PI	Principal Investigator
PIMMC	potentially immune-mediated medical condition
QA	Quality Assurance
QC	Quality Control
QDP	QuaDPharma
QMP	Quality Management Plan
SAE	Serious Adverse Event/Serious Adverse Experience
SMC	Safety Monitoring Committee
SOP	Standard Operating Procedure
Th-1	Type 1 T helper cells
Th-17	Type 17 T helper cells
Th-2	Type 2 T helper cells
TMP	transmembrane pressure
TNA	toxin-neutralizing antibody
TNF-α	tumor necrosis factor alpha
TRIS	tris(hydroxymethyl)aminomethane
TRIS/HCl	tris(hydroxymethyl)aminomethane/hydrochloric acid
TRM	tissue-resident T memory
US	United States
USP	United States Pharmacopeia
$W_{80}5EC$	oil-in-water nanoemulsion adjuvant
WHO	World Health Organization
wks	weeks

PROTOCOL SUMMARY

**Title:**	A Phase 1 Randomized**,** Double-Blind, Active- and Placebo-Controlled, Safety, Tolerability**,** and Immunogenicity Study of Two Doses of Intranasal Recombinant H5 Influenza Vaccine with and without Nanoemulsion Adjuvant Followed by One Boosting Dose of Intramuscular Influenza A (H5N1) Vaccine in Healthy Adult Volunteers
**Design of the Study:**	This is a Phase 1, first-in-human, single-center, randomized, placebo-controlled, double-blind study to assess the safety, tolerability, and immunogenicity of a primary series of intranasal recombinant H5 influenza vaccine with and without nanoemulsion adjuvant followed by boosting dose of licensed, intramuscular influenza A (H5N1) vaccine. Because this is a dose escalation trial, our study has four stages with one cohort receiving vaccines at each stage. Up to 40 participants will be randomized to one of the five study groups at an allocation ratio depending on the escalation stage. If all participants proceed to vaccination, the final vaccine allocation ratio will be 1:1:1:1:1. Subjects will receive a primary series of two intranasal vaccinations of study treatment administered on Days 1 and 29. Subject dosing will proceed in a stepwise process. Each dose of adjuvanted study vaccine will be assessed in sentinel participants before the remainder of the study group is vaccinated and before proceeding to vaccination of sentinel participants with the next higher dose of adjuvanted study vaccine. Following receipt of the first vaccine dose, sentinel participants will be followed for 7 days for halting criteria and SMC data review prior to proceeding with vaccination of the remainder of the study group. All participants will subsequently receive a third dose of intramuscular, heterologous influenza A (H5N1) vaccine on Day 197. Participants will be followed for safety and immunology endpoints for one year following their second study treatment vaccination.
**Study Phase:**	1
**Study Population:**	Up to 40 healthy adults aged 18 through 45 years, inclusive.
**Number of Sites:**	One (1) US Site:<br>Center for Vaccine Development and Global Health<br>University of Maryland School of Medicine<br>685 W. Baltimore St.<br>Baltimore, MD 21201

**Description of Study Product or Intervention:**	**BW-1014,** a nanoemulsion-adjuvanted recombinant H5 (A/Indonesia, clade 2.1) influenza vaccine administered intranasally, consisting of:<br>• **Recombinant influenza A H5 (rH5) antigen vaccine** produced by Fraunhofer USA Center for Molecular Biology (FhCMB).<br>• **Oil-in-water nanoemulsion W_{80}5EC,** a mucosal adjuvant produced by BlueWillow Biologics, Inc.<br>• **Formulation buffer**<br>• **Sterile water**<br><br>**Active Control** (identical to BW-1014 but without nanoemulsion adjuvant)<br>• **Recombinant influenza A H5 (rH5) antigen vaccine** produced by Fraunhofer USA Center for Molecular Biology (FhCMB).<br>• **Formulation buffer**<br>• **Sterile water**<br><br>**Inactive Control (Placebo)** consisting of formulation buffer.<br><br>**Influenza Virus Vaccine, H5N1,** (H5N1 IIV) a licensed, split virion vaccine (A/Vietnam, clade 1) produced by Sanofi Pasteur approved to be administered intramuscularly for active immunization of persons 18 through 64 years of age at increased risk of exposure to the H5N1 influenza virus subtype contained in the vaccine.<br><br>See Table 1 below for details of the five (5) treatment groups.
**Study Objectives:**	Primary:<br>• To assess safety and reactogenicity of two doses of intranasal rH5 vaccine (clade 2.1) with and without 20%W_{80}5EC adjuvant<br>Secondary:<br>• To assess the primary mucosal immune responses (by IgA; IgG; and vaccine-specific activation markers, cytokines/chemokines and degranulation markers by CD4 and CD8 T cell memory subsets) in nasal wash specimens to homologous H5N1 (clade 2.1) after two doses of intranasal rH5 vaccine (clade 2.1) with and without 20%W_{80}5EC adjuvant<br>• To assess safety and reactogenicity of one dose of intramuscular H5N1 IIV (clade 1) given approximately six months after the series of two doses of intranasal rH5 vaccine with and without 20%W_{80}5EC adjuvant<br>• To assess the primary humoral immune responses (by HI, IgA, and IgG), in serum to homologous H5N1 (clade 2.1) after two doses of intranasal rH5 vaccine (clade 2.1) with and without 20% W_{80}5EC adjuvant<br>Exploratory

	• To assess the primary humoral immune responses (by HI) to heterologous H5N1 (clade 1) in serum after two doses of intranasal rH5 vaccine (clade 2.1) with and without 20%W805EC adjuvant<br>• To assess the anamnestic humoral immune responses (by HI, IgA, IgG, influenza A antibody stem) to homologous H5N1 (clade 2.1) after two doses of intranasal rH5 vaccine (clade 2.1) with and without 20% W805EC adjuvant and one dose of intramuscular heterologous H5N1 IIV (clade 1)<br>• To assess the anamnestic humoral immune responses (by HI) to heterologous H5N1 (clade 1) after two doses of intranasal rH5 vaccine (clade 2.1) with and without 20%W805EC adjuvant followed by one dose of intramuscular heterologous H5N1 IIV (clade 1)<br>• To assess the primary T cellular immune responses (by Flow Cytometry) to homologous H5N1 (clade 2.1) after two doses of intranasal rH5 vaccine (clade 2.1) with and without 20% W805EC adjuvant<br>• To assess the anamnestic T cellular immune responses (by Flow Cytometry) to homologous H5N1 (clade 2.1) after two doses of intranasal rH5 vaccine (clade 2.1) with and without 20%W805EC adjuvant and one dose of intramuscular heterologous H5N1 vaccine (clade 1)<br>• To assess the humoral immune response in serum to the phylogenetic group 1 hemagglutinin stem<br>• To assess the mucosal immune response in nasal wash fluid to homologous H5N1 (clade 2.1) by HI after two doses of intranasal rH5 vaccine (clade 2.1) with and without 20%W805EC adjuvant followed by one dose of intramuscular heterologous H5N1 IIV (clade 1)<br>• To assess the durability of the primary humoral immune responses to homologous H5N1 (clade 2.1) and to heterologous (clade 1) by HI after two doses of intranasal rH5 vaccine (clade 2.1) with and without 20% W805EC adjuvant<br>• To assess memory B cell responses in peripheral blood (by B cell ELISpot) to homologous H5N1 (clade 2.1) after two doses of intranasal rH5 vaccine (clade 2.1) with and without 20%W805EC adjuvant as well as after one dose of intramuscular heterologous H5N1 vaccine (clade 1)
**Duration of Individual Subject Participation:**	13 months from enrollment

**Estimated Time to Last Subject/Last Study Day:**	2 years

Table 1. Study Treatment Arms

Group	No. of Subjects	Dose 1 and 2 (intranasal)	rH5 (µg)	Nanoemulsion (NE) Adjuvant	Total Volume (mL)	Dose 3 (intramuscular), antigen content (µg) / volume (mL)
A	8	BW-1014: 25 µg	25	20%	0.5	H5N1 IIV (90 µg/1mL)
B	8	BW-1014: 50 µg	50	20%	0.5	H5N1 IIV (90 µg/1mL)
C	8	BW-1014: 100 µg	100	20%	0.5	H5N1 IIV (90 µg/1mL)
D	8	rH5 Control: 100 µg	100	0%	0.5	H5N1 IIV (90 µg/1mL)
E	8	Placebo	0	0%	0.5	H5N1 IIV (90 µg/1mL)

H5N1 IIV = Sanofi Pasteur Inactivated Influenza Virus Vaccine, H5N1

1 KEY ROLES

Lead Principal Investigator:

Justin R. Ortiz, MD, MS, FACP, FCCP
Professor of Medicine
Center for Vaccine Development
University of Maryland School of Medicine

DMID:

Clinical Project Manager:
Melinda Tibbals
5601 Fishers Lane
Rockville MD 20852-9825
Work: (240) 627-3387
Email: melinda.tibbals@nih.gov

Medical Monitor:
Tatiana Beresnev, MD
5601 Fishers Lane
Rockville MD 20852-9825
Work: 301-761- 7062
Email: beresnet@mail.nih.gov

Program Officer:
Brooke Bozick, PhD
5601 Fishers Lane
Rockville, MD 20852
Work: 301-761- 6710
Email: brooke.bozick@nih.gov

**Statistical and Data Coordinating Center:**	Jessica P. Brown, Ph.D.Director of Clinical and Translational Informatics Center (CTRIC)<br>Department of Epidemiology and Public Health<br>University of Maryland School of Medicine<br><br>Yuanyuan Liang, Ph.D., M.S.<br>Professor in the Department of Epidemiology and Public Health University of Maryland School of Medicine<br>Section Head for Biostatistics at Center for Vaccine Development<br>University of Maryland School of Medicine

2 BACKGROUND AND SCIENTIFIC RATIONALE

2.1 Background

This study is a Phase I, first-in-human, single-center, randomized, placebo-controlled, double-blind study to assess the safety, tolerability and immunogenicity of a primary series of two doses of mucosal BW-1014 administered intranasally followed by one boosting dose of licensed, intramuscular H5N1 vaccine in healthy adult volunteers aged 18 through 45 years.

2.1.1 Investigational Product

BW-1014 is a nanoemulsion-adjuvanted recombinant H5 (A/Indonesia, clade 2.1) influenza vaccine (rH5) administered intranasally; three dose levels will be evaluated. The boosting dose will be the licensed, intramuscular Influenza Virus Vaccine, H5N1 (A/Vietnam, clade 1.1) vaccine (H5N1 IIV). BW-1014 is developed, manufactured, and provided by BlueWillow Biologics, Ann Arbor, MI, USA (BW) using rH5 antigen provided by Fraunhofer USA Center for Molecular Biotechnology, Plymouth, MI, USA (FhCMB). The H5N1 IIV is manufactured by Sanofi Pasteur, Swiftwater, PA, USA and donated by the Biomedical Advanced Research and Development Authority (BARDA) of the US Department of Health and Human Services (HHS).

Notably, the BW nanoemulsion combined with seasonal inactivated influenza vaccine (Fluzone®, Sanofi Pasteur, Swiftwater, PA) has been previously studied as an intranasally administered vaccine in a phase I trial of healthy adults,[1]and the FhCMB rH5 has been previously studied in a phase I trial as a intramuscular formulation in healthy adults.[2] This proposed trial will evaluate mechanisms of mucosal response to H5N1 vaccination and help to advance new immunization strategies to address an urgent unmet public health need.

2.1.2 Influenza Disease

Influenza virus circulates globally, affects people of all ages, and causes annual disease outbreaks. Influenza A viruses undergo frequent antigenic mutations (called antigenic drift) that allow the viruses to evade immune protection and cause repeated influenza virus infections in an individual over a lifetime. Novel virus strains may emerge through genetic reassortment of influenza virus ribonucleic acid (RNA) from different strains in a common host.[3] Further, non-human strains may be directly infecting and cause disease in humans.[4] Reassortment events or direct infection of humans by animal viruses have the potential to cause a pandemic if humans have little to no pre-existing immunity to the virus, if person-to-person transmission is sustained, and if infection causes clinical disease.[5] While the emergence of a pandemic virus could be catastrophic for human health, pandemics also have major negative effects on the functioning of society and economies.[6]

Each of the four pandemics in the last 100 years has been caused by influenza viruses with animal influenza genetic components.[7] Since its emergence in 1996, avian influenza A (H5N1) has caused wide-spread epizootic disease in birds and sporadic human infections. The World

Health Organization (WHO) has confirmed 860 human cases of H5N1 virus infection with >50% case fatality since 2003.[8] Circulating HPAI H5N1 virus strains include clades and subclades 1.1, 2.1, 2.2, 2.3, and 7.[9] Further subdivisions of antigenically distinct circulating subclades have also been described, and they continue to undergo antigenic drift.[10,11] Clades that have infected humans include 0, 1, 2, and 7.[12]

In 1999, NIH identified H5N1 as the novel influenza virus with the greatest threat to human health and began supporting vaccine development, a process which resulted in two FDA-approved H5N1 vaccines (detailed below).[13]To prepare and respond to pandemic influenza threats, BARDA established and maintains the National Pre–Pandemic Influenza Vaccine Stockpile (NPIVS).[14] The goal of NPIVS is to store vaccines for influenza viruses with pandemic potential to vaccinate 20 million people in the critical workforce in the event of a pandemic.[14] Current US pandemic plans call for the mobilization and distribution of stockpiled H5N1 vaccines as a first line of defense in the event of an H5N1 pandemic.

2.1.3 Current Seasonal Influenza Vaccines

Vaccination is the best approach to prevent influenza, and many licensed influenza vaccines are available on the global market. These vaccines fall into two broad categories: intramuscularly administered inactivated influenza vaccines (IIVs) and intranasally administered live-attenuated influenza vaccines (LAIVs). Current vaccines are further classified based on production substrate (eggs, cell), antigen dose and valence, and the presence or absence of adjuvants. Current IIVs are designed with the common goal to induce immunity to the hemagglutinin (HA) surface glycoprotein of the influenza virus. As the viral surface HA undergoes frequent antigenic drift, the seasonal influenza vaccine is reformulated as often as twice annually to match the strains projected to circulate in the following influenza season.

In addition to the cumbersome twice annual reformulation and production required to respond to antigenic change, the performance of current influenza vaccines is suboptimal and varies considerably by year, vaccine formulation, and the underlying age, health condition, and prior virus and vaccine exposure of the recipient.[15] Depending on the degree to which vaccine strains match circulating strains, seasonal IIV effectiveness will vary, as antibody against influenza is for the most part strain-specific. Over the last 14 years, the US Centers for Disease Control and Prevention (CDC) estimates that influenza vaccine effectiveness in the United States has had an annual average of 40% among all persons studied, and there were 2 years with no significant vaccine effectiveness.[16] Most licensed seasonal influenza vaccines are produced in egg culture in a process that is largely unchanged since the first IIVs were developed in the 1930s. From the identification of component viruses, production of seed viruses for use by manufacturers, growth and harvesting of egg-cultured viruses, manufacturing, assurance of potency, evaluation of performance, and packaging and distribution, traditional vaccines require around 7 months before product release. In the event of a pandemic, this lag ensures that no influenza vaccines will be developed in time to sufficiently control global virus spread.[17] Egg culture production of influenza vaccines is problematic, as some vaccine seed viruses have had difficulty growing in eggs,[18] and H3N2 virus adaptations to growth in egg culture have resulted in vaccine viruses

being antigenically distinct from the seed virus and circulating influenza virus.[19] Newer technologies have been developed to overcome some of the limitations of current vaccines. Oil-in-water adjuvanted vaccines and high dose vaccines elicit greater immune responses than traditional IIVs and are approved in the United States for persons 65 years and over. In head-to-head comparisons, high dose vaccines have shown superior efficacy to standard dose.[20-22] LAIVs have previously shown superior efficacy in children as compared to IIVs, though recent observational studies have not shown this advantage. Nasally delivered vaccines are detailed further below. The first fully recombinant vaccine was approved by the FDA in 2017.[23]Both recombinant and cell-based vaccines may overcome the egg-adaptation of vaccine strains that contribute to diminished vaccine effectiveness.[24]**Licensed H5N1 Vaccines**

The US government has supported the development of avian influenza vaccines for national pandemic preparedness. The two licensed H5N1 vaccines are both against clade 1 viruses and include Influenza Virus Vaccine, H5N1, a monovalent, inactivated, split virus vaccine (Sanofi Pasteur Inc., Swiftwater, PA, USA) and Q-Pan H5N1, a monovalent, inactivated, split virus vaccine with ASO3 adjuvant (ID Biomedical Corporation of Quebec, a subsidiary of GlaxoSmithKline, Quebec City, QC, Canada). Both H5N1 vaccines are made using similar processes to seasonal IIVs. In adults, Influenza Virus Vaccine, H5N1 is administered in two doses of 90µg HA content intramuscularly 28 days apart, while Q-Pan H5N1 is administered in two doses of 3.75µg HA content with ASO3 adjuvant intramuscularly 21 days apart. The clinical development of both vaccines was hampered by absence of existing immunity to H5N1 viruses in the general population and the poor immunogenicity of the vaccine H5 antigens, leading to the need for a two dose primary series, as well as the use of formulations with either high dose (Influenza Virus Vaccine, H5N1) or adjuvants (Q-Pan).[25] Influenza Virus Vaccine, H5N1 was licensed with a seroprotection rate of 46% for HI antibodies,[14] lower than the FDA specified success criterion of 70%,[26] while Q-Pan achieved this threshold.

Both H5N1 vaccines are stockpiled in the NPIVS, as are adjuvants for potential use in combination. In a Phase II trial sponsored by BARDA in 2016 and 2017, the safety and immunogenicity of two doses of stockpiled Influenza Virus Vaccine, H5N1 with and without MF59 adjuvant (an unlicensed combination) was assessed.[14] The antigens used in the trial had been in storage since 2004 or 2005. The safety profile was similar to previous studies of antigen alone vaccines or of MF59-adjuvanted vaccines. The vaccines were immunogenic, and the 90 µg group without adjuvant had post dose two hemagglutination inhibition (HI) geometric mean titer (GMT, 19.6) and seroprotection (34.1%) that were similar to the previous trial conducted for product licensure.[27] Vaccines with lower antigen content (7.5µg or 15µg) and MF59 adjuvant achieved higher seroprotection (65.9% and 64%). Moreover, the 90µg group without adjuvant achieved from 5-30% cross-reactive seroprotection against more contemporary strains of H5N1.

2.1.5 Need for New Vaccines

Seasonal and pandemic influenza are major public health threats. Vaccination is the best approach to prevent influenza, and many influenza vaccines are available on the global market. These vaccines fall into two broad categories: intramuscularly administered inactivated influenza

vaccines (IIV) and intranasally administered live-attenuated influenza vaccines (LAIV), and they can be further classified based on production substrate (eggs, cell, recombinant), antigen dose and valence and the presence or absence of adjuvants. Current IIVs are designed with the common goal to induce humoral immunity to the hemagglutinin (HA) surface glycoprotein of the influenza virus. As the viral surface glycoproteins undergo antigenic drift, the seasonal influenza vaccine is reformulated as often as twice annually to match the strains projected to circulate in the following influenza season.

In addition to the cumbersome manufacturing process required to respond to constant antigenic change, the performance of current influenza vaccines is suboptimal and varies considerably by year, vaccine formulation, and the underlying age, health condition and prior virus and vaccine exposure history of the recipient. Unfortunately, the relative contribution of each of these factors has not been well-studied, given the many variables involved and the complex interplay of exposure and host response. Depending on the degree to which the vaccine strains match the circulating strains, seasonal influenza vaccines will confer more or less protection, as antibody against influenza is for the most part strain-specific. The US CDC recently reported that the average influenza vaccine effectiveness in the US over the last 14 years was around 40%, including two years (2004-05 and 2005-06) where there was no significant vaccine effectiveness at all.[16]

The burden of seasonal influenza illness, the continuing threat of a pandemic, and the inadequacy of current influenza vaccines emphasize the urgent need for improved influenza vaccines. Recognizing the limitations of current influenza vaccines, NIH, WHO, BARDA and vaccine manufacturers have prioritized development of improved influenza vaccines that stimulate cellular and mucosal immune responses, protect more broadly against antigenically dissimilar viruses, and that can be feasibly administered during public health emergencies. Realizing that development of a universal influenza vaccine that meets all these criteria is a long-term goal, NIAID and others fully endorse the development of vaccines that will incrementally improve the breadth and duration of influenza protection.

Many infections occur via mucosal surfaces, including the respiratory, gastrointestinal, and urogenital surfaces. The mucosal immune system is therefore an important component of the body's defense against such infections. Consequently, induction of mucosal, in addition to systemic immunity, is important for protection from disease. Several vaccines that target the mucosal immune system are available e.g., oral polio vaccine, rotavirus vaccine, Ty21a (anti-S. Typhi; Vivitof), CVD 103-HgR (anti-cholera; Vaxchora), live-attenuated anti-influenza virus vaccines (FluMist), etc. The respiratory mucosal immune system consists of a complex and integrated network of tissues including nasopharynx-associated lymphoid tissue (NALT), which represents the first line of defense against pathogens. The presence of innate cells and their activation in this site during influenza infection have been shown. Importantly, in the gut and lungs a special TM subset, dubbed tissue-resident T memory (TRM), is generally acknowledged to provide the first line of defense against invading pathogens. Nevertheless, it is unknown whether TRM cells are present in the upper respiratory mucosa (initial site of influenza virus

infection) and their role in protection and/or infection control. Moreover, despite that IgA is known to play a role in protection from mucosal pathogens, including influenza virus, the presence of influenza-specific B cells in mucosal surfaces remains unexplored.

The purpose of this study is to evaluate a proof-of-principle that influenza A HA glycoproteins administered intranasally with or without nanoemulsion adjuvant can induce a detectable mucosal immune response in seronegative adults. The primary focus will be on evaluating the safety of this first-in-human vaccine. In addition, however, measures of mucosal, humoral, and cellular immunity will be assessed at a multiple time points before, during, and after a primary series of study vaccination to establish baseline measures of immunological response for this new immunization strategy. As there are no correlates of mucosal immunity to influenza virus infection, and as mucosal influenza immunization may not induce systemic immune responses comparable to intramuscular immunization, this study includes an innovative third, booster, dose of licensed intramuscular H5N1 vaccine. This dose will be given in order to probe the immune system to evaluate whether priming was achieved with the primary vaccine series, even in the setting of equivocal or absent measurable humoral immunity after the primary series. This trial will evaluate mechanisms of mucosal protection to influenza virus infection, and potentially help to establish new immunization strategies to address a current unmet public health need.

2.2 Scientific Rationale

2.2.1 Purpose of Study

The primary purpose of the study is to assess the safety, reactogenicity and tolerability of two doses of rH5 vaccine (BW-1014, with differing antigen content - 25, 50 and 100 μg) administered intranasally, followed by one dose of intramuscular H5 vaccine in healthy adults aged 18 through 45 years. An active control (rH5 antigen in formulation buffer) and a placebo control (formulation buffer alone) are also included.

Key study outcomes are to establish the safety profile of the intranasal (IN) BW-1014 vaccine, to characterize the mucosal, humoral and cellular immune response following two doses of vaccine, and to evaluate whether immunological priming can be uncovered by dosing of intramuscular, H5N1 IIV at 6 months following the primary BW-1014 series. Left-over clinical specimens will be stored, and additional clinical specimens will also be collected for the purpose of potential future use.

2.2.2 Study Population

The population of this study is healthy, adult volunteers aged 18 through 45 years, inclusive. Subjects will be drawn from volunteers presenting to the Center for Vaccine Development at the University of Maryland School of Medicine. Proximal location to the research site will help to ensure adherence of protocol schedule of clinic visits.

2.3 Potential Risks and Benefits

2.3.1 Potential Risks

The potential risks of participating in this trial are those associated with having blood drawn, the collection of nasal wash specimens, the collection of nasopharyngeal swab specimens, the collection of nasal absorptive matrix specimen, the collection of nasal mucosal scraping specimen, possible reactions to the IN BW-1014 vaccine (including antigen, adjuvant, and components), possible reactions to the IM injection of the H5N1 vaccine, and breach of confidentiality.

There is a small amount of risk to subjects who report that they are in good health but who have an unknown health problem at the time of screening. Experience with the influenza A (H5N1) vaccine and the rH5 vaccines given intramuscularly indicate a reactogenicity profile similar to other inactivated influenza vaccines (IIVs). IIVs can be associated with influenza-like reactions, such as fever, feverishness, fatigue, malaise, myalgia, arthralgia, headache, and/or nausea. Some subjects may develop reactions at the injection site, including pruritus, ecchymosis, erythema, induration, edema, pain, and/or tenderness.[28] Most of these reactions peak in intensity in the first 24 hours after vaccination and disappear without treatment within 1 or 2 days. Analgesics and rest may generally relieve or lessen these reactions. Bruising can sometimes occur due to the vaccination procedure.

BW-1014 given intranasally is expected to have similar reactogenicity profile as intranasal seasonal IIV with nanoemulsion adjuvant, which was associated with increased local symptoms throat pain, rhinorrhea, and nasal congestion.[1]

Acute and potentially life-threatening allergic reactions (i.e., anaphylaxis) are also possible, even for those in whom no history exists. These reactions occur in about 1 in 4 million people given a seasonal influenza vaccination. These reactions can manifest as hives, angioedema, difficulty breathing, tachycardia, or hypotension. If these reactions occur, they can usually be stopped by the administration of emergency medications by the study personnel. As with any vaccine or medication, there is a very small chance of a death, although researchers do not expect this to occur. Adjuvants have been shown to induce broader immune responses to immunization, particularly T cell responses, broadening the immunological mechanisms of protection but also increasing the theoretical risk of vaccine induced immune-mediated disease.[29] These may also include, but are not limited to, neuritis, convulsions, severe allergic reactions, syncope, encephalitis, thrombocytopenia, vasculitis, and Guillain-Barré syndrome. Given the association between a previously licensed IN IIV (Nasalflu previously produced by Berna Biotech) and Bell’s Palsy, participants may also be at increased risk for seventh cranial nerve disorders.

While the SARS-CoV-2 pandemic is on-going, there is a risk of exposure to others with SARS-CoV-2 infection at the in-person visits. We will minimize this risk by requiring the subjects have received COVID-19 vaccination, reminding subjects to inform us of COVID-19 symptoms prior to clinic visits, by inquiring of COVID-19 symptoms upon subject arrival for clinic visits, and by

testing subjects for COVID-19 within two days of BW-1014 vaccination. We will also maintain infection control procedures consistent with current CDC and institutional guidance. Subjects with symptoms consistent with COVID-19 or testing positive for COVID-19 will not be vaccinated until symptoms have resolved and sufficient time has passed, per current CDC guidance. Likewise, all study personnel will follow up-to-date CDC guidelines on personal protective equipment and will be vaccinated against COVID-19.

2.3.2 Potential Benefits

There are no known benefits attributable to the receipt of intranasal BW-1014 or a single dose of intramuscular H5N1 IIV. Vaccination may or may not provide protection against a serious disease with the influenza H5N1 virus, should the participant be exposed. The duration of any such protection is currently unknown. The study products are not expected to offer protection against circulating seasonal influenza viruses. There may be public health benefits to society in the future if the vaccine and adjuvants being evaluated in this clinical trial prove to be sufficiently safe and immunogenic and can lead to new licensed influenza prevention strategies or vaccines.

3 STUDY DESIGN, OBJECTIVES AND ENDPOINTS OR OUTCOME MEASURES

This is a Phase I, first-in-human, single-center, randomized, placebo-controlled, double-blind study to assess the safety, tolerability and immunogenicity of a primary series of intranasal rH5 (A/Indonesia) influenza vaccine with and without 20%W_{80}5EC adjuvant followed by one boosting dose of licensed, intramuscular H5N1 (A/Vietnam) vaccine in healthy adult volunteers aged 18 through 45 years, inclusive. Participants from a single center will be randomized to one of the five study groups depending on the dose escalation stage, and they will receive a primary intranasal vaccine series administered on Days 1 and 29. On Day 197 (6 months after enrollment) all participants will receive a dose of intramuscular, inactivated influenza A (H5N1), subsequently referred as “H5N1 IIV”. A summary of study groups is provided in Table 1.

BW-1014 antigen content was determined based on a desire to balance the necessary dose for humoral immune response in ferret dose ranging studies while minimizing participant exposure in this first-in-human study. The rH5 antigen in BW-1014 is derived from H5N1 (A/Indonesia, clade 2.1). While H5N1 IIV (A/Vietnam, clade 1) is licensed as a two-dose series, only one dose will be used to serve as an immunologic probe to uncover potential immunologic priming induced by the primary vaccine series. This H5N1 vaccine was chosen because it allows the evaluation of a heterologous H5N1 antigen boost, and because it is licensed, minimizing participant exposure to experimental vaccines. The study has been designed to focus narrowly on the study objectives listed in Section 3.1. Investigational, mucosal vaccine components will be donated through agreements with BlueWillow Biologics (60%W_{80}5EC, Formulation Buffer) and Fraunhofer (rH5). Licensed H5N1 IIV from the US Strategic National Stockpile will be donated through agreements with BARDA.

The study has four stages with one cohort receiving vaccine at each stage. Up to 40 participants will be randomized to one of the five study groups at an allocation ratio depending on the escalation stage. If all 40 participants proceed to vaccination, the final vaccine allocation ratio will be 1:1:1:1:1. The study investigational product compounding will be conducted by the CVD Tennant Lab. Tennant Lab personnel and designated study personnel will be unblinded and ensure the appropriate product is administered to each study subject. Designated unblinded study personnel will not be involved in safety or immunogenicity assessments.

Individual subject participation will be thirteen (13) months, thus following subjects for 1 year following their second vaccine in the primary treatment series and 6 months following the H5N1 IIV boost. It is expected that the entire study will require 14-15 months to complete the last subject visit.

Subjects will be dosed in an escalating fashion (Table 2). The study will be divided into four cohorts. Within the first three cohorts of participants receiving adjuvanted study vaccine, two sentinel participants will be vaccinated and followed for at least 7 days for halting criteria

(Section 8.6.1) and SMC data review. Each dose of adjuvanted study vaccine will be assessed in sentinel participants before the remainder of the study group is vaccinated and before proceeding to vaccination of sentinel participants with the next higher dose of adjuvanted study vaccine (next cohort). Each cohort will also include participants receiving comparator vaccines (unadjuvanted rH5 control and placebo) to minimize bias in assessment of vaccine performance.

Table 2. Sentinel Subjects and Dose Escalation Schedule

Cohort	Group	No. of Subjects	Dose 1 and 2
1	A	2	Sentinel Low Dose (BW-1014: 25 µg) with NE adjuvant
	D	2	rH5 Control: 100 µg
	E	2	Placebo
	Total	6	
2	A	6	Low Dose (BW-1014: 25 µg) with NE adjuvant
	B	2	Sentinel Middle Dose (BW-1014: 50 µg) with NE adjuvant
	D	2	rH5 Control: 100 µg
	E	2	Placebo
	Total	12	
3	B	6	Middle Dose (BW-1014: 50 µg) with NE adjuvant
	C	2	Sentinel High Dose (BW-1014: 100 µg) with NE adjuvant
	D	2	rH5 Control: 100 µg
	E	2	Placebo
	Total	12	
4	C	6	High Dose (BW-1014: 100 µg) with NE adjuvant
	D	2	rH5 Control: 100 µg
	E	2	Placebo
	Total	10	

Blood, nasal wash, and nasal mucosal scraping specimens used to assess humoral, mucosal, and cellular immunity will be collected prior to the initial vaccination in the primary series (baseline) and post-intranasal vaccination, immediately prior to IM H5N1 vaccination, and post IM H5N1 vaccination. Some specimens will be collected for future use only. A full description of the specimens collected by timepoint, can be found in Appendix G Procedure Volumes. Nasal wash, PBMC, and nasal mucosal scraping specimens will go to the Center for Vaccine Development (CVD) Cellular Immunology Lab for flow cytometry assessments. Nasal absorptive matrix, saliva, unused serum, and unused nasal wash samples will be stored for future use by CVD Cellular Immunology Lab. NP swabs will be used to test for COVID-19 at the University of Maryland, Baltimore. Serum samples will go to the CVD Applied Immunology Laboratory for hemagglutination inhibition (HI) assays.

3.1 Study Objectives

The purpose of the study is to inform selection of the optimal dose level(s) for further study. This purpose applies to all primary and secondary objectives.

3.1.1 Primary

- To assess safety and reactogenicity of two doses of intranasal rH5 vaccine (clade 2.1) with and without 20%W_{80}5EC adjuvant

3.1.2 Secondary

- To assess the primary mucosal immune responses (by IgA; IgG; and vaccine-specific activation markers, cytokines/chemokines and degranulation markers by CD4 and CD8 T cell memory subsets) in nasal wash specimens to homologous H5N1 (clade 2.1) after two doses of intranasal rH5 vaccine (clade 2.1) with and without 20%W_{80}5EC adjuvant
- To assess safety and reactogenicity of one dose of intramuscular H5N1 IIV (clade 1) given approximately six months after the series of two doses of intranasal rH5 vaccine with and without 20%W_{80}5EC adjuvant
- To assess the primary humoral immune responses (by HI, IgA, and IgG), in serum to homologous H5N1 (clade 2.1) after two doses of intranasal rH5 vaccine (clade 2.1) with and without 20% W_{80}5EC adjuvant

3.1.3 Exploratory

- To assess the primary humoral immune responses (by HI) to heterologous H5N1 (clade 1) in serum after two doses of intranasal rH5 vaccine (clade 2.1) with and without 20%W805EC adjuvant
- To assess the anamnestic humoral immune responses (by HI, IgA, IgG, influenza A antibody stem) to homologous H5N1 (clade 2.1) after two doses of intranasal rH5 vaccine (clade 2.1) with and without 20% W805EC adjuvant and one dose of intramuscular heterologous H5N1 IIV (clade 1)
- To assess the anamnestic humoral immune responses (by HI) to heterologous H5N1 (clade 1) after two doses of intranasal rH5 vaccine (clade 2.1) with and without 20%W805EC adjuvant followed by one dose of intramuscular heterologous H5N1 IIV (clade 1)
- To assess the primary T cellular immune responses (by Flow Cytometry) to homologous H5N1 (clade 2.1) after two doses of intranasal rH5 vaccine (clade 2.1) with and without 20% W805EC adjuvant
- To assess the anamnestic T cellular immune responses (by Flow Cytometry) to homologous H5N1 (clade 2.1) after two doses of intranasal rH5 vaccine (clade 2.1) with and without 20%W805EC adjuvant and one dose of intramuscular heterologous H5N1 vaccine (clade 1)
- To assess the humoral immune response in serum to the phylogenetic group 1 hemagglutinin stem
- To assess the mucosal immune response in nasal wash fluid to homologous H5N1 (clade 2.1) by HI after two doses of intranasal rH5 vaccine (clade 2.1) with and without

20%W805EC adjuvant followed by one dose of intramuscular heterologous H5N1 IIV (clade 1)

- To assess the durability of the primary humoral immune responses to homologous H5N1 (clade 2.1) and to heterologous (clade 1) by HI after two doses of intranasal rH5 vaccine (clade 2.1) with and without 20% W805EC adjuvant
- To assess memory B cell responses in peripheral blood (by B cell ELISpot) to homologous H5N1 (clade 2.1) after two doses of intranasal rH5 vaccine (clade 2.1) with and without 20%W805EC adjuvant as well as after one dose of intramuscular heterologous H5N1 vaccine (clade 1)

3.2 Study Endpoints or Outcome Measures

3.2.1 Primary Endpoints

3.2.1.1 Primary Endpoints: Safety and Reactogenicity of intranasal BW-1014

The following outcomes will be used in assessing the primary objective:

- Number and proportion of participants reporting solicited or unsolicited local or systemic reactions within 1 hour of intranasal BW-1014, positive control, and placebo administration, including visual assessment of nasal passages, by study arm [Time Frame: Day 1 and Day 29 post-vaccination]
- Number and proportion of participants reporting solicited reactions and general AEs within 7 days of vaccination with intranasal BW-1014, positive control and placebo, by study arm [Time Frame: Day 8 and Day 36, for previous 7-day period]
- Number and proportion of participants reporting unsolicited AEs within 28 days of primary vaccinations with intranasal BW-1014, positive control and placebo, by study arm [Time Frame: Day 29 and Day 57, for previous 28-day period]
- Number and proportion of participants reporting any hematological and biochemical laboratory abnormality (Class 1 of higher) within 7 days of first dose intranasal BW-1014 or within 14 days second dose intranasal BW-1014, positive control, and placebo, by study arm [Time Frame: Day 8, Day 43 and Day 204]
- Number and proportion of participants reporting medically attended AEs (MAAEs) within 28 days of primary vaccinations with intranasal BW-1014, positive control, and placebo, by study arm [Time Frame: Day 29 and Day 57, for previous 28-day period]
- Number and proportion of participants reporting serious adverse events (SAEs), by study arm [Time Frame: across entire study]
- Number and proportion of participants reporting potential immune-mediated medical conditions (PIMMCs), by study arm [Time Frame: across entire study]
- Number and proportion of participants reporting new onset chronic medical conditions (NOCMCs), by study arm [Time Frame: across entire study]

3.2.2 Secondary Endpoints

3.2.2.1 Secondary Endpoints: Mucosal Immunogenicity

The following outcomes will be used in assessing mucosal immunogenicity in nasal wash fluid following two doses of BW-1014 (clade 2.1) vaccine or the positive control:

- Vaccine-specific IgA Geometric Mean Titer (GMT) by ELISA [Time Frame: prior to 1 vaccination, Day 43, Day 57, Day 197]
- Vaccine-specific IgG Geometric Mean Titer (GMT) by ELISA [Time Frame: prior to 1 vaccination, Day 43, Day 57, Day 197]
- In cells isolated from the nasal cavity, presence/absence of vaccine-specific activation markers (CD69 and HLA-DR), cytokines/chemokines (IFN-γ, TNF-α, IL-2, IL-17A) and degranulation markers (CD107a) by CD4 and CD8 T cell memory subsets (effector memory, central memory, effector memory CD45RA+) as determined by Flow Cytometry. [Time Frame: Day 57 and Day 197]

3.2.2.2 Secondary Endpoints: Safety and Reactogenicity of Intramuscular H5N1 IIV

The following outcomes will be used in assessing the secondary safety objective:

- Number and proportion of participants reporting solicited or unsolicited local or systemic reactions within 1 hour of vaccination with intramuscular H5N1 IIV vaccine, by study arm [Time Frame: Day 197 post-vaccination]
- Number and proportion of participants reporting solicited reactions and general AEs within 7 days of vaccination with intramuscular H5N1 IIV vaccine, by study arm [Time Frame: Day 204, for previous 7-day period]
- Number and proportion of participants reporting unsolicited AEs within 28 days of vaccinations with intramuscular H5N1 IIV vaccine [Time Frame: Day 225, for previous 28-day period]
- Number and proportion of participants reporting any hematological and biochemical laboratory abnormality (Class 1 of higher) within 7 days of vaccination with intramuscular H5N1 IIV vaccine, by study arm [Time Frame: Day 204]
- Number and proportion of participants reporting medically attended AEs (MAAEs) within 28 days of vaccination with intramuscular H5N1 IIV vaccine, by study arm [Time Frame: Day 225, for previous 28-day period]

3.2.2.3 Secondary Endpoint: Primary Humoral Immune Response

The following outcomes will be used to assess primary humoral immune response to homologous H5N1 (clade 2.1) after two doses of intranasal rH5 vaccine (clade 2.1) with and without 20%W_{80}5EC adjuvant in serum:

- HI endpoints
  - Rates of seroconversion, defined as the percentage of subjects with either a pre-vaccination HI titer < 1:10 and a post-vaccination HI titer ≥ 1:40 or a pre-vaccination HI titer ≥ 1:10 and a minimum of four-fold rise in post-vaccination HI antibody titer. [Time Frame: prior to 1st vaccination and Day 57]
  - GMTs
- IgA and IgG endpoints

- Strain-specific GMT by ELISA [Time Frame: prior to 1st vaccination and Day 57]

3.2.3 Exploratory Endpoints

3.2.3.1 Exploratory Endpoint: Primary Humoral Immune Response to Heterologous (to primary series) H5N1 virus

The following outcomes will be used to assess the primary humoral immune response (by HI) to heterologous H5N1 (clade 1) after two doses of intranasal rH5 (clade 2.1) vaccine with and without 20%W_{80}5EC adjuvant in serum:

- HI Endpoints
  - Rates of seroconversion, defined as the percentage of subjects with either a pre-vaccination HI titer < 1:10 and a post-vaccination HI titer ≥ 1:40 or a pre-vaccination HI titer ≥ 1:10 and a minimum of four-fold rise in post-vaccination HI antibody titer. [Time Frame: prior to 1st vaccination and Day 57]
  - Geometric Mean Titers (GMTs) [Time Frame: Day 57]

3.2.3.2 Exploratory Endpoints: Anamnestic Humoral Immune Response to Homologous H5N1 virus

The following outcomes will be used to assess the anamnestic humoral immune response (by HI, IgA, IgG, influenza A antibody stem) to homologous H5N1 (clade 2.1) after two doses of intranasal rH5 (clade 2.1) vaccine with and without 20%W_{80}5EC adjuvant and one dose of intramuscular H5N1(clade 1) vaccine in serum:

- HI Endpoints
  - Rates of seroconversion, defined as the percentage of subjects with either a pre-vaccination HI titer < 1:10 and a post-vaccination HI titer ≥ 1:40 or a pre-vaccination HI titer ≥ 1:10 and a minimum four-fold rise in post-vaccination HI antibody titer. [Time Frame: Day 197 and Day 225]
  - Geometric Mean Titers (GMTs) [Time Frame: Day 225]
- IgA and IgG Endpoints
  - Strain-specific GMT by ELISA [Time Frame: Day 225]

3.2.3.3 Exploratory Endpoints: Anamnestic Humoral Immune Response to Heterologous H5N1 virus

The following outcomes will be used to assess the anamnestic humoral immune response (by HI) to heterologous H5N1 (clade 1) after two doses of intranasal rH5 (A/clade 2.1) vaccine with and without 20%W_{80}5EC adjuvant followed by a subsequent dose of intramuscular H5N1 (clade 1) vaccine:

- HI Endpoints
  - Rates of seroconversion, defined as the percentage of subjects with either a pre-vaccination HI titer < 1:10 and a post-vaccination HI titer ≥ 1:40 or a pre-vaccination HI titer ≥ 1:10 and a minimum four-fold rise in post-vaccination HI antibody titer. [Time Frame: Day 197 and Day 225]
  - Geometric Mean Titers (GMTs) [Time Frame: Day 225]

3.2.3.4 Exploratory Endpoints: Primary Cellular Immune Responses

The following outcomes will be used to assess the primary cellular immune response (by Flow Cytometry and/or ELISpot) to homologous H5N1 (clade 2.1) after two doses of intranasal rH5 (clade 2.1) vaccine with and without 20%W_{80}5EC adjuvant in PBMCs:

- Presence/absence of cell activation markers (e.g., CD69 and HLA-DR), cytokines/chemokines (e.g., IFN-γ, TNF-α, IL-2, IL-17A) and degranulation markers (e.g., CD107a) by CD4 and CD8 T cell memory subsets (effector memory, central memory, effector memory CD45RA+) as determined by Flow Cytometry [Time Frame: prior to 1st vaccination (Day 0) and Day 57]
- B cell responses. These studies will include the measurement of memory B cell responses to rH5 by ELISpot and/or flow cytometry, and other assays of B cell function. [Time Frame: prior to 1st vaccination (Day 0) and Day 57]

3.2.3.5 Exploratory Endpoints: Anamnestic Cellular Immune Response

The following outcomes will be used to assess the anamnestic cellular immune response (by Flow Cytometry and/or ELISpot) to homologous H5N1 (clade 2.1) after two doses of intranasal rH5 (clade 2.1) vaccine with and without 20%W_{80}5EC adjuvant and one dose of intramuscular H5N1 (clade 1) vaccine in PBMCs:

- Presence/absence of cell activation markers (e.g., CD69 and HLA-DR), cytokines/chemokines (e.g., IFN-γ, TNF-α, IL-2, IL-17A) and degranulation markers (e.g., CD107a) by CD4 and CD8 T cell memory subsets (effector memory, central memory, effector memory CD45RA+) as determined by Flow Cytometry [Time Frame: Days 197 and 225]
- B cell responses. These studies will include the measurement of memory B cell responses to rH5 by ELISPOT and/or flow cytometry, and other assays of B cell function [Time Frame: Days 197 and 225]

3.2.3.6 Exploratory Endpoints: Phylogenetic Group 1 Hemagglutinin Anti-Stem Response

The following outcomes will be used to assess primary humoral immune response to homologous H5N1 (clade 2.1) after two doses of intranasal rH5 vaccine (clade 2.1) with and without 20%W805EC adjuvant in serum:

- Influenza A phylogenetic group 1 stem-specific IgG GMT by ELISA [Time Frame: prior to 1st vaccination, and Days 57 and 197]

3.2.3.7 Exploratory Endpoints: Mucosal Immune Response by Hemagglutination Inhibition to Homologous H5N1 virus

The following outcomes will be used in assessing mucosal immunogenicity in nasal wash fluid following two doses of BW-1014 (clade 2.1) vaccine or the positive control:

- Vaccine-specific GMT hemagglutination inhibition (GMT HI) [Time Frame: prior to 1st vaccination, Day 57, Day 197]

3.2.3.8 Exploratory Endpoints: Durability of Primary Humoral Immune Response

The following outcomes will be used to assess the durability of the primary humoral immune response to homologous H5N1 (clade 2.1) after two doses of intranasal rH5 vaccine (clade 2.1) with and without 20%W805EC adjuvant in serum:

- HI endpoints
    - Rates of seroconversion, defined as the percentage of subjects with either a pre-vaccination HI titer < 1:10 and a post-vaccination HI titer ≥ 1:40 or a pre-vaccination HI titer ≥ 1:10 and a minimum of four-fold rise in post-vaccination HI antibody titer. [Time Frame: prior to 1st vaccination, and Day 57 and Day 197]
    - GMTs
- IgA and IgG endpoints
    - Strain-specific GMT by ELISA [Time Frame: prior to 1st vaccination, and Day 57 and Day 197]

4 STUDY INTERVENTION/INVESTIGATIONAL PRODUCT

4.1 Study Product Description

4.1.1 Formulation, Packaging, and Labeling

Manufacturing and packaging of the formulation ingredients will be performed for the study by the CVD Tennant Lab (heretofore referred to as the “study investigational product compounding facility”) and will be documented in conformity with Good Manufacturing Practice (GMP) per instructions provided in the Study Drug Preparation Manual.

The individual subject vaccines must be prepared from bulk and stored in capped, glass vials at 2-8ºC until ready for use; prepared treatments must be administered to the clinical trial subjects within 48 hours of formulation in the investigational product compounding facility.

The study products will be labeled according to manufacturer or regulatory specifications and include the statement “Caution: New Drug – Limited by Federal Law to Investigational Use.”

<u>BW-1014</u>

The candidate rH5 vaccine BW-1014 is an adjuvanted, liquid formulation administered via the intranasal (IN) route. The vaccine will be prepared in three concentrations: 25, 50 or 100 µg of rH5 adjuvanted with 20% W_{80}5EC in formulation buffer.

Four individual drug product components as shown in Table 3 will be used in the formulation of the BW-1014 treatments.

Table 3. BW-1014 Vaccine Drug Product, Components and Suppliers

Component	Composition	Supplier of Component
rH5 in PBS	rH5 (1.87 mg/mL) in PBS pH 7.4	Fraunhofer USA Center for Molecular Biology (FhCMB).
Formulation Buffer	Sterile water, TRIS (pH 8.0), sodium chloride, histidine, sucrose	Study investigational product compounding facility (CVD Tennant Lab)
Sterile Water for Injection	Sterile water	Cytiva (Marlborough, MA)
60%W_{80}5EC Nanoemulsion Adjuvant	Purified water, soybean oil, dehydrated alcohol, polysorbate 80, cetylpyridinium chloride (milky white appearance)	BlueWillow Biologics, Inc.
BW-1014 Vaccine	Combination of above 4 components to yield 25, 50 or 100 µg rH5 per 500 µL dose (final concentration of 50, 100, and 200 µg/mL) with 20% W_{80}5EC in formulation buffer. Total dose volume of 500µL, 250µL/nare. (milky white appearance).	Will be formulated extemporaneously by the clinical trial study investigational product compounding facility (CVD Tennant Lab)

rH5 Control: The study investigational product compounding facility will be responsible for the formulation and vialing of the rH5 control. The composition of the rH5 control is summarized in Table 4.

Table 4. rH5 Control

Component	Composition	Supplier of Component
rH5 Control	rH5 (1.87 mg/mL) in PBS pH 7.4	Fraunhofer USA Center for Molecular Biology (FhCMB).
Formulation Buffer	**Sterile water, TRIS (pH 8.0), sodium chloride, histidine, sucrose**	Study investigational product compounding facility (CVD Tennant Lab)
Sterile water for injection	Sterile water	Cytiva (Marlborough, MA)
Positive (rH5) control	Combination of above 3 components to yield 100µg rH5 per 500 µL dose (final concentration of 200 µg/mL) in formulation buffer. Total dose volume of 500µL, 250µL/nare (clear appearance).	Will be formulated extemporaneously by the clinical trial study investigational product compounding facility (CVD Tennant Lab)

Placebo: The study investigational product compounding facility will be responsible for the formulation and vialing of the placebo control. The composition of the placebo control is summarized in Table 5.

Table 5. Placebo

Component	Composition	Supplier of Component
Formulation Buffer facility	**Sterile water, TRIS (pH 8.0), sodium chloride, histidine, sucrose**	Study investigational product compounding facility (CVD Tennant Lab)
Sterile water for injection	Sterile water	Cytiva (Marlborough, MA)
Placebo Control	Combination of above 2 components to yield formulation buffer and water. Total dose volume of 500µL, 250µL/nare (clear appearance).	Will be formulated extemporaneously by the clinical trial study investigational product compounding facility (CVD

Influenza Virus Vaccine, H5N1 (H5N1 IIV): The H5N1 IIV manufactured by Sanofi Pasteur will be used as the antigen boost vaccine delivered to all subjects on Day 197 and will be provided per agreement with BARDA from the NPIVS. The vaccine is supplied in 5.0 mL multi-dose vials, each containing five 1.0 mL doses.

4.1.2 Product Storage and Stability

All vaccines used in this study must be stored at 2-8ºC.

4.2 Acquisition/Distribution

BW-1014 Vaccines: Table 3 provides the suppliers for each component for the BW-1014 vaccine.

rH5 Control: The rH5 antigen used in the rH5 control is the same as that used in the formulation of the BW-1014 vaccine and is supplied by FhCMB.

Placebo: The formulation buffer composing the placebo control is the same as that used in the formulation of the BW-1014 vaccine; it will be formulated by the study investigational product compounding facility.

H5N1 IIV: The H5N1 IIV manufactured by Sanofi Pasteur will be provided via an agreement with BARDA from the NPIVS.

4.3 Dosage/Regimen, Preparation, Dispensing and Administration of Study Intervention/Investigational Product

All individual subject vaccines are held at 2-8ºC until ready for use. The vials should be allowed to acclimatize to room temperature (20-40 minutes) before administration. Table 6 summarizes the treatment dose, volume, route and timing for each vaccine in the study.

Table 6. Summary of Treatments, Dosages, Routes and Timing of Administration

Product Name	Dose rH5 (µg)	Dose $W_{80}5EC$ (%)	Volume	Route	Timing of Administration
BW-1014 (all dose strengths)	25<br>50<br>100	20%	500µL (250/nare)	Intranasal	Day 1 and Day 29
rH5 control	100	0	500µL (250/nare)	Intranasal	Day 1 and Day 29
Placebo	0	0	500µL (250/nare)	Intranasal	Day 1 and Day 29
H5N1 IIV (antigen boost)	90	0	1 mL	Intramuscular	Day 197 (all subjects)

4.3.1 Intranasal Administration Procedure Using Eppendorf Pipette

IN administration will be performed using a metered electronic pipettes (Eppendorf Repeater E3) and Combitips by designated unblinded study personnel (nurses and/or clinician sub-investigators). This is the same device and administration procedure that was used to deliver nasal vaccine in two prior influenza vaccine clinical trials (IND # 13968 and 14671) and one prior anthrax vaccine clinical trial (IND# 19054). No contamination concerns were reported in these prior clinical trials as a result of using this device.

Subjects will be asked to blow their nose gently before vaccination. Subjects should close their eyes to ensure they remain blinded to the treatment. Every subject will be vaccinated using a new sterile Combitip for investigational product dosing. After attaching the Combitip to the pipette, the investigational product will be aspirated from the dosing vial. Subjects will lie supine on a bed or examination table. Following positioning of the subject, the nasal cavity will be inspected using a nasal speculum to determine the position of the inferior turbinate. The administrator will stabilize the subject's head and position the Eppendorf Electronic Repeater pipette tip into the subject's nostril (no more than 1.0 cm of the pipette tip should be inside the nares), and then administer the vaccine on the inferior turbinate. The administrator should be careful during administration to ensure that the subject will not inhale the investigational product. A 250 µL volume will be administered to each nare in a series of ten, successive, 25 µL volumes with a brief pause between each drop. This will be repeated for the other nare, for a total dose volume of 500 µL per subject.

After the last drop has been administered, the subject will turn their head to the left and to the right. The administrator may depress the nostril(s) gently for a moment, then request that the subject sniff gently prior to slowly returning to the midline position. Following these procedures should minimize any liquid dripping from the nose or being swallowed down the back of the throat. The subject may gargle with plain water if they experience stinging or burning in the back of their throat following vaccine administration.

If the subject sits up and/or sneezes during administration, they should be asked if they wish to continue the administration. If they wish to continue, they can be placed back in a supine position and administration can resume. If they do not wish to continue, the number of drops administered prior to stopping should be recorded. In either case, a description of the disruption in administration should be recorded.

Complete instructions for administering the IN vaccine is provided in the Manual of Procedures.

4.3.2 Intramuscular Administration of H5N1 IIV Vaccine

Study vaccine administration will be performed by study personnel credentialed to administer vaccines and who may also participate in dose preparation. Each dose (1 mL) of the boost vaccine will be administered to subjects via a single intramuscular injection given in the deltoid muscle of the subjects' preferred arm on the day of study vaccination. The site of injection (right or left arm) will be recorded on the appropriate data collection form. Complete instructions for administering the IM vaccine are provided in the Manual of Procedures.

4.4 Accountability Procedures for the Study Intervention/Investigational Product(s)

BlueWillow will ensure study vaccine materials are provided to the study investigational product compounding facility, except for the formulation buffer which will be prepared by the facility. Once received, vaccine materials will be stored in and dispensed by the facility or in another suitable location within the CVD. Unused materials may be destroyed.

The FDA requires accounting for the disposition of all investigational products. The Investigator is responsible for ensuring that a current record of product disposition is maintained and product is dispensed only at an official study site by authorized personnel as required by applicable regulations and guidelines. Records of product disposition, as required by federal law, consist of the date received, date administered, quantity administered, and the subject number to whom the drug was administered.

The investigational product compounding facility will be responsible for maintaining accurate records of the shipment and dispensing of the investigational product. The records must be available for inspection and will be subject to inspection by a regulatory agency (e.g., FDA) at any time. An assigned Study Monitor will review the facility records.

Unused investigational product vials will be stored at 2- 8C in the study investigational product compounding facility or a secure CVD site until clinical trial accountability is completed. At study termination, all unused investigational product will be disposed in accordance with the MOP following complete drug accountability and monitoring.

5 SELECTION OF SUBJECTS AND STUDY ENROLLMENT AND WITHDRAWAL

Subjects will be recruited from in and around the Baltimore, Maryland area. Interested volunteers will contact the CVD Recruitment Office in response to advertisements, flyers, or other forms of IRB-approved notification. Healthy adults from the region will be screened for eligibility. After listening to an IRB-approved telephone script, interested volunteers will be provided an appointment for a Screening Session. During the Screening Session, a copy the study consent form will be provided, and a study clinician will provide a verbal detailed description of all aspects of the study, including rationale and background, public health significance, procedures, schedule of visits, risks, benefits, and subject rights. After the informed consent form is signed, a member of the study team will administer a brief examination to assess the volunteer's comprehension of the study (i.e., Comprehension Assessment Tool); a passing score is ≥70% correct answers (two attempts permitted). The consent form will be signed prior to performance of any screening procedures. Staff will then review the eligibility criteria with volunteers.

Subject Inclusion and Exclusion Criteria must be confirmed by a study clinician licensed to make medical diagnoses.

No exemptions will be granted on Subject Inclusion/Exclusion Criteria. Questions about eligibility will be directed toward the PI and, if needed, DMID Medical Officer.

5.1 Eligibility Criteria

5.1.1 Subject Inclusion Criteria

Subjects must meet all Inclusion Criteria to be eligible for study participation:

1. Healthy men or women aged 18 through 45 years of age, inclusive.
2. Women must not be pregnant or nursing. If premenopausal, absence of pregnancy must be confirmed by a negative serum pregnancy test conducted at screening and a negative urine pregnancy test conducted at the site within 24 hours preceding receipt of vaccine.
3. Women who are not surgically sterile or at least one year post-menopausal must agree to use acceptable birth control. Acceptable birth control methods include oral, implantable, transdermal or injectable contraceptive; barrier methods such as condoms, cervical caps, or diaphragms with spermicide; abstinence from sexual relationships with a male partner, non-male sexual relationships, monogamous relationship with vasectomized partner who has been vasectomized for 180 days or more prior to the subject receiving the first study vaccination, and other reliable forms of contraception approved by the Investigator. Acceptable birth control must be used for a minimum of 30 days prior to vaccination and for 3 months following final study vaccination.

4. Subjects must be in good general health, as determined by medical history and physical examination. Acceptable vital signs and clinical laboratory examinations are within the normal range per study toxicity tables (Appendix B). Vital signs and clinical laboratory examinations meeting Grade 1 criteria may also be acceptable in the opinion of the PI or appropriate Sub-Investigator. For this study, an appropriate Sub-Investigator is a designated clinician licensed to make medical diagnoses and listed on the Form FDA 1572. Exclusionary clinical laboratory examinations may be repeated one time to assess clinical improvement in the event temporary halting criteria (described in Section 8.6.2) are met at the time of the first examination.
5. Subjects must be able to comprehend the study requirements as evidenced by a score of ≥ 70% on the comprehension assessment (two attempts permitted), be available for the required study period, and have the ability to attend scheduled visits.
6. Subjects must be able to provide written informed consent to participate in the study.
7. Subject agrees to future use of left-over specimens and to the collection of additional specimens for potential future use research.
8. Receipt of the CDC-recommended number of doses of an EUA authorized or licensed COVID-19 vaccine product ≥ four weeks prior to first study vaccination.

5.1.2 Subject Exclusion Criteria

Subjects meeting any of the Exclusion Criteria are NOT eligible for study participation:

1. Presence of significant acute or chronic, uncontrolled medical or psychiatric illness (institution of new medical or surgical treatment, or a significant dose alteration for uncontrolled symptoms or drug toxicity within 3 months). This includes signs or symptoms consistent with upper or lower respiratory tract infections.
2. Participants with symptoms of COVID-19 and/or who are positive for SARS-CoV-2 by molecular diagnostic assay conducted within 2 days pre-vaccination.
3. Have known hypersensitivity or allergy to eggs, egg or chicken protein, or other components of the Influenza Virus Vaccine, H5N1 (Sanofi Pasteur).
4. Receipt of licensed or experimental H5N1 influenza vaccine ever.
5. Subjects with a history of chronic cough, frequent sinus infections, sinusitis, allergic rhinitis, nasal polyps or obstruction, including deviated septum significant enough to obstruct the nasal openings or a history of nasal surgery.
6. Body mass index (BMI) BMI ≤ 18.5 or ≥ 40.
7. Positive serology for human immunodeficiency virus (HIV)-1 or HIV-2, hepatitis B, or hepatitis C (HCV).
8. Platelet count less than 100,000/mm prior to randomization during baseline visit.
9. History of drug or chemical abuse within the past year prior to screening.
10. History of aspiration, dysphagia, swallowing disorders, stroke or other neurologic conditions that may predispose the subject to aspiration of test articles into the respiratory tract.
11. History of Bell’s palsy.
12. History of Guillain-Barré syndrome within 6 weeks of prior influenza virus vaccine.

13. Cancer or treatment for cancer, within 3 years. Basal cell carcinoma or squamous cell carcinoma are allowed, unless present on or near the nose.
14. Impaired immune responsiveness, including a history of diabetes mellitus.
15. Chronic use of inhaled or intranasal sprays including decongestants and corticosteroids.
16. A current vaper, smoker or tobacco user or a history of smoking or tobacco use within the past year prior to screening.
17. Receipt or history of receiving any medications or treatments that affected the immune system such as immune globulin, interferon, immunomodulators, cytotoxic drugs or drugs known to be frequently associated with significant major organ toxicity, or systemic corticosteroids (oral or injectable) in the past 6 months.
18. Has received or has plans to receive any licensed or authorized vaccines within 2 weeks (for inactivated vaccines) or 4 weeks (for live vaccines) prior to first study vaccination through Day 57.
19. History of allergic and/or anaphylactic type reaction to injected influenza vaccines or to any of the components of BW-1014 [soybean oil, dehydrated alcohol (anhydrous ethanol), polysorbate (Tween 80), cetylpyridinium chloride (CPC), and tobacco].
20. Receipt of any investigational product or nonregistered drug within the 30 days before screening or currently enrolled in any investigational drug study or intends to enroll in such a study before completion of in person clinical evaluations.
21. Use of nasally administered prescription or over-the-counter medications within 7 days before vaccination.
22. Receipt of blood or blood products 8 weeks before screening or planned administration prior to the Week 8 visit.
23. Donation of blood or blood products within 8 weeks before screening or at any time up to the Week 4 clinic visit.

If a subject presents at screening or on a vaccination date with an acute illness, the Investigator will refer to Individual Halting Criteria (Section 8.6.2) to assess whether to temporarily delay enrollment or vaccination until the illness is resolved.

5.2 Withdrawal from the Study, Discontinuation of Study Product, or Study Termination

5.2.1 Withdrawal from the Study or Discontinuation of the Study Product

Subjects may voluntarily withdraw their consent for study participation at any time without penalty or loss of benefits to which they are otherwise entitled.

The Investigator may also withdraw a subject from receiving the study product for any reason. Follow-up safety evaluations will be conducted if the subject agrees. If a subject withdraws or is withdrawn prior to completion of the study, the reason for this decision must be recorded in the case report forms (CRFs).

The reasons, might include, but are not limited to the following:

- Subject no longer meets eligibility criteria
- Subject meets individual halting criteria (refer to Section 8.6.2)
- Subject becomes noncompliant
- Medical disease or condition, or new clinical finding(s) for which continued participation, in the opinion of the Investigator, might compromise the safety of the subject, interfere with the subject's successful completion of this study, or interfere with the evaluation of responses
- Subject lost to follow-up
- Subject becomes pregnant, if applicable
- Determined by the PI or appropriate Sub-Investigator discretion to require additional therapy not indicated in the protocol to ensure subject's health and well-being (or treatment failure, if applicable)

The Investigator should be explicit regarding study follow-up (e.g., safety follow-up) that might be carried out despite the fact the subject will not receive further study product. If the subject consents, every attempt will be made to follow all AEs through resolution. The procedures that collect safety data for the purposes of research must be inclusive in the original informed consent or the Investigator may seek subsequent informed consent using an IRB/IEC-approved consent form with the revised procedures.

The Investigator will inform the subject that data already collected will be retained and analyzed even if the subject withdraws from this study.

5.2.2 Subject Replacement

Subjects who withdraw from the study after initial immunization will not be replaced. Safety assessment should be encouraged for a full year following last dose. Subjects who decide to withdraw from the study prior to or during the first immunization could be replaced. The investigational product compounding facility personnel and the designated unblinded study personnel (nurses and/or clinician sub-investigators) will provide a replacement dose from the same vaccine arm. Designated unblinded study personnel will not be involved in safety or immunogenicity assessments.

5.2.3 Study Termination

If the study is prematurely terminated by the sponsor, any regulatory authority, or the Investigator for any reason, the Investigator will promptly inform the study subjects and assure appropriate therapy or follow-up for the subjects, as necessary. The Investigator will provide a detailed written explanation of the termination to the IRB/IEC.

6 STUDY PROCEDURES

6.1 Screening (-45 to -3 days prior to randomization)

6.1.1 Visit 1: Screening

All subjects will undergo a screening evaluation to include medical history, physical exam and drug use screening to evaluate their health status and study eligibility. Females of childbearing potential will have a serum pregnancy test. This screening will be conducted no more than 45 days prior to the first planned immunization (Day 1).

- Obtain informed consent: Prior to initiating any study procedures, including screening procedures, informed consent will be obtained from each study subject.
- Review inclusion/exclusion criteria: Review inclusion/exclusion criteria against subject's current health status to ensure that subject is eligible to participate in the study.
- Collect demographics: Age, sex, race and ethnicity will be recorded.
- Collect medical and surgical history: All significant past and current diagnoses will be documented. All signs and symptoms (regardless of severity) and medication allergies that the subject has experienced within 30 days prior to screening will be documented.
- Collect drug, alcohol and smoking history: Information on historical and current use of alcohol and tobacco will be collected. Historical information on drug abuse will also be collected and a drug screening assessment performed by the study clinician.
- Collect prior and concomitant medications: Refer to Section 7.1.2 for details.
- Collect weight and height and calculate BMI.
- Collect vital signs: Refer to Section 7.1.1 for details.
- Perform baseline physical examination: Refer to Section 7.1.1 for details.
- Perform baseline targeted neurologic/otorhinoscopic/respiratory examination: Refer to Section 7.1.1 for details.
- Perform serum pregnancy test: Serum pregnancy test will be used to rule out pregnancy in female subjects of childbearing potential only.
- Perform erythrocyte sedimentation rate and serology testing: Refer to Section 7.2.1.
- Collect samples for clinical safety Hematology and Chemistry: Refer to Section 7.2.1.
- Collect nasal wash sample.
- Collect saliva sample.
- Collect nasal absorptive matrix sample.

6.2 Baseline, Randomization and Dosing (Day 1)

6.2.1 Visit 2: Baseline (pre-dose on Day 1)

Prior to randomization and dosing, the following assessments will be completed:

- Review inclusion/exclusion criteria: Review inclusion/exclusion criteria against subject's current health status to ensure that subject remains eligible to participate in the study.
- Review and update medical history.
- Review and update prior and concomitant medications.

- Collect weight and calculate BMI.
- Collect vital signs: Refer to Section 7.1.1 for details.
- Perform directed physical examination: Refer to Section 7.1.1 for details. This exam is directed to new complaints or symptoms to assess for changes since screening visit. New findings will be assessed and documented as medical history.
- Perform targeted neurologic/otorhinoscopic/respiratory examination: Refer to Section 7.1.1 for details. This exam is directed to new complaints or symptoms to assess for changes since screening visit. New findings will be assessed and documented as medical history.
- Perform urine pregnancy test: If female subject of childbearing potential, complete a urine pregnancy test to establish pregnancy status. Pregnancy status must be negative in order for subject to receive first immunization.
- Collect serum sample.
- Collect PBMC sample.
- Collect nasopharyngeal swab sample for SARS-CoV-2 testing by molecular diagnostic assay. Result must be negative for SARS-CoV-2 before any vaccination can occur. Note that this procedure can occur at any time within two days of vaccination.

6.2.2 Visit 2: Randomization and Dosing (Day 1)

If the subject remains eligible (i.e., he/she passed all the inclusion criteria and do not meet any exclusion criteria), the subject will be randomized to a treatment arm and the following actions will be taken:

- Confirm SARS-CoV-2 test is negative.
- Complete pre-vaccination reactogenicity assessment.
- Randomize subject to Study Group. Immunize subject per randomization: See Section 4.3.1 for instructions.
- Observe participant for reactogenicity for 1 hour post-vaccination.
- Obtain vital signs at 1 hour following immunization.
- Perform targeted neurologic/otorhinoscopic/respiratory examination at 1 hour following immunization.
- Assess acute reactogenicity: Refer to Section 8.1.2 for details.
- Dispense Symptom Diary: Provide copy of the Symptom Diary to subject and instruct subject on its daily completion for the 7 days following immunization.
- Assess Adverse Events (AEs) and Serious Adverse Events (SAEs).

6.3 Planned Study Visits

6.3.1 Visit 3: Safety Call (Day 4 ± 2 days)

- Place phone call to subject.
- Review and update prior and concomitant medications.
- Remind subject to continue completing the Symptom Diary daily.
- Assess occurrence of AEs beyond symptoms on the Symptom Diary.
- Assess for occurrence of SAEs.

6.3.2 Visit 4: Safety (Day 8 ± 2 days)

- Collect vital signs.
- Review and update concomitant medications.
- Conduct directed physical examination: This physical exam is directed to new complaints or symptoms to assess for changes since prior visit; if there are clinically significant findings on exam, these should be reported as an AE and participant will be monitored for resolution of the finding.
- Perform targeted neurologic/otorhinoscopic/respiratory examination: Refer to Section 7.1.1 for details. This exam is directed to new complaints or symptoms to assess for changes since prior visit; if there are clinically significant findings on exam, these should be reported as an AE and participant will be monitored for resolution of the finding.
- Collect Symptom Diary: Discuss diary with subject, evaluate symptoms of reactogenicity over the last 7 days, and complete reactogenicity assessment.
- Assess AEs and SAEs.
- Collect samples for clinical safety Hematology and Chemistry.
- Nasal wash sample will be collected.
- Collect saliva sample.
- Collect nasal absorptive matrix sample.
- Collect nasal mucosal scraping sample. If limited cell numbers are obtained from nasal scrapings, specimens from the same dose group/visit will be pooled by an unblinded member of the clinical team. Pooled specimens will be given a separate code. The coded specimens (pooled) will be processed by the lab personnel, who will remain blinded.

6.3.3 Visit 5: Dose #2 (Day 29 ± 4 days)

The following assessments will be performed prior to second immunization:

- Review inclusion/exclusion criteria: Review inclusion/exclusion criteria against subject's current health status to ensure that subject remains eligible to participate in the study.
- Review and update concomitant medications.
- Collect weight and calculate BMI.
- Collect vital signs.
- Conduct directed physical examination: This physical exam is directed to new complaints or symptoms to assess for changes since prior visit; if there are clinically significant findings on exam, these should be reported as an AE and participant will be monitored for resolution of the finding.
- Perform targeted neurologic/otorhinoscopic/respiratory examination: Refer to Section 7.1.1 for details. This exam is directed to new complaints or symptoms to assess for changes since prior visit; if there are clinically significant findings on exam, these should be reported as an AE and participant will be monitored for resolution of the finding.
- Assess for the absence of acute illness or new medical conditions including any signs and symptoms of upper or lower respiratory tract or symptoms of infection with SARS-CoV-2. Subjects should not receive a second dose if they have any of these signs or symptoms.

- Perform urine pregnancy test: If female subject of childbearing potential, complete a urine pregnancy test to establish pregnancy status. Pregnancy status must be negative in order for subject to receive second immunization.
- Collect samples for clinical safety Hematology and Chemistry.
- Serum sample will be collected.
- Collect nasopharyngeal swab sample for SARS-CoV-2 testing by molecular diagnostic assay. Result must be negative for SARS-CoV-2 before any vaccination can occur. Note that this procedure can occur at any time within two days of vaccination.

The following activities and assessments will be completed after the pre-immunization assessments are complete

- Confirm SARS-CoV-2 test is negative.
- Complete pre-vaccination reactogenicity assessment.
- Immunize subject per randomization: See Sections 4.3.1 for instructions.
- Observe participant for reactogenicity for 1 hour post-vaccination.
- Obtain vital signs at 1hour following immunization.
- Perform targeted neurologic/otorhinoscopic/respiratory examination at 1 hour following immunization.
- Assess acute reactogenicity: Refer to Section 8.1.2 for details.
- Dispense Symptom Diary: Provide copy of the Symptom Diary to subject and instruct them on its daily completion for the 7 days following immunization.
- Assess AEs and SAEs.

6.3.4 Visit 6: Safety Call (Day 32 ± 2 days)

- Place phone call to subject.
- Review and update prior and concomitant medications.
- Remind subject to continue completing the Symptom Diary.
- Assess occurrence of AEs beyond symptoms on the Symptom Diary.
- Assess for occurrence of SAEs.

6.3.5 Visit 7: Safety (Day 43 ± 2 days)

- Review and update concomitant medications.
- Collect vital signs.
- Conduct directed physical examination: This physical exam is directed to new complaints or symptoms to assess for changes since prior visit; if there are clinically significant findings on exam, these should be reported as an AE and participant will be monitored for resolution of the finding.
- Perform targeted neurologic/otorhinoscopic/respiratory examination: Refer to Section 7.1.1 for details. This exam is directed to new complaints or symptoms to assess for changes since prior visit; if there are clinically significant findings on exam, these should be reported as an AE and participant will be monitored for resolution of the finding.
- Collect Symptom Diary: Discuss diary with subject, evaluate symptoms of reactogenicity over the last 7 days, and complete reactogenicity assessment.
- Assess AEs and SAEs.

- Collect samples for clinical safety Hematology and Chemistry.
- Nasal wash sample will be collected.
- Serum sample will be collected.
- PBMC sample will be collected.
- Saliva sample will be collected.
- Collect nasal absorptive matrix sample.

6.3.6 Visit 8: Safety (Day 57 ± 4 days)

- Review and update concomitant medications.
- Collect vital signs.
- Conduct directed physical examination: This physical exam is directed to new complaints or symptoms to assess for changes since prior visit; if there are clinically significant findings on exam, these should be reported as an AE and participant will be monitored for resolution of the finding.
- Perform targeted neurologic/otorhinoscopic/respiratory examination: Refer to Section 7.1.1 for details. This exam is directed to new complaints or symptoms to assess for changes since prior visit; if there are clinically significant findings on exam, these should be reported as an AE and participant will be monitored for resolution of the finding.
- Collect serum sample.
- Collect PBMC sample.
- Nasal wash sample will be collected.
- Assess AEs and SAEs.
- Saliva sample will be collected.
- Collect nasal absorptive matrix sample.
- Collect nasal mucosal scraping sample. If limited cell numbers are obtained from nasal scrapings, specimens from the same dose group/visit will be pooled by an unblinded member of the clinical team. Pooled specimens will be given a separate code. The coded specimens (pooled) will be processed by the lab personnel, who will remain blinded.

6.3.7 Visit 9: Safety Call (Day 90 ± 7 days)

- Review and update prior and concomitant medications.
- Assess for any neurologic/otorhinoscopic/respiratory complaints. A positive response would trigger an unscheduled clinic visit for clinical examination, targeted neurologic/ otorhinoscopic/respiratory examination and further evaluation as indicated.
- Assess for occurrence of SAEs.

6.3.8 Visit 10: H5N1 IIV Boost Vaccine (Day 197 ± 7 days)

The following assessments will be performed prior to IM immunization with H5N1 IIV:

- Review inclusion/exclusion criteria: Review inclusion/exclusion criteria against subject's current health status to ensure that subject remains eligible to participate in the study.
- Review and update concomitant medications.
- Collect weight and calculate BMI.
- Collect vital signs.

- Conduct directed physical examination: This physical exam is directed to new complaints or symptoms to assess for changes since prior visit; if there are clinically significant findings on exam, these should be reported as an AE and participant will be monitored for resolution of the finding.
- Perform targeted neurologic/otorhinoscopic/respiratory examination: Refer to Section 7.1.1 for details. This exam is directed to new complaints or symptoms to assess for changes since prior visit; if there are clinically significant findings on exam, these should be reported as an AE and participant will be monitored for resolution of the finding.
- Perform urine pregnancy test: If female subject of childbearing potential, complete a urine pregnancy test to establish pregnancy status. Pregnancy status must be negative for in order for subject to receive H5N1 IIV immunization.
- Collect samples for clinical safety Hematology and Chemistry.
- Collect Serum sample.
- Nasal wash sample will be collected.
- Saliva sample will be collected.
- PBMC sample will be collected.
  Collect nasal absorptive matrix sample. If limited cell numbers are obtained from nasal scrapings, specimens from the same dose group/visit will be pooled by an unblinded member of the clinical team. Pooled specimens will be given a separate code. The coded specimens (pooled) will be processed by the lab personnel, who will remain blinded.

The following activities and assessments will be completed after the pre-immunization assessments are complete:

- Complete pre-vaccination reactogenicity assessment.
- Immunize subject: See Section 4.3.2 for instructions.
- Observe participant for reactogenicity for 30 minutes post-vaccination.
- Obtain vital signs at 30 minutes following immunization.
- Assess acute reactogenicity: Refer to Section 8.1.2 for details.
- Dispense Symptom Diary: Provide copy of the Symptom Diary to subject and instruct them on its daily completion for the 7 days following immunization.
- Assess AEs and SAEs.

6.3.9 Visit 11: Safety Call (Day 200 ± 2 days)

- Place phone call to subject.
- Review and update prior and concomitant medications.
- Remind subject to continue completing the Symptom Diary.
- Assess occurrence of AEs beyond symptoms on the Symptom Diary.
- Assess for occurrence of SAEs.

6.3.10 Visit 12: Safety (Day 204 ± 2 days)

- Review and update concomitant medications.
- Collect vital signs.
- Conduct directed physical examination: This physical exam is directed to new complaints or symptoms to assess for changes since prior visit; if there are clinically significant

findings on exam, these should be reported as an AE and participant will be monitored for resolution of the finding.
- Perform targeted neurologic/otorhinoscopic/respiratory examination: Refer to Section 7.1.1 for details. This exam is directed to new complaints or symptoms to assess for changes since prior visit; if there are clinically significant findings on exam, these should be reported as an AE and participant will be monitored for resolution of the finding.
- Collect Symptom Diary: Discuss diary with subject, evaluate symptoms of reactogenicity over the last 7 days, and complete reactogenicity assessment.
- Collect samples for clinical safety Hematology and Chemistry.
- Assess AEs and SAEs.
- Nasal wash sample will be collected.
- Saliva sample will be collected.
- Serum sample will be collected.

6.3.11 Visit 13: Safety (Day 225 ± 7 days)

- Review and update concomitant medications.
- Collect vital signs.
- Conduct directed physical examination: This physical exam is directed to new complaints or symptoms to assess for changes since prior visit; if there are clinically significant findings on exam, these should be reported as an AE and participant will be monitored for resolution of the finding.
- Perform targeted neurologic/otorhinoscopic/respiratory examination: Refer to Section 7.1.1 for details. This exam is directed to new complaints or symptoms to assess for changes since prior visit; if there are clinically significant findings on exam, these should be reported as an AE and participant will be monitored for resolution of the finding.
- Assess AEs and SAEs.
- Nasal wash sample will be collected.
- Saliva sample will be collected.
- Serum sample will be collected.
- PBMC will be collected.
- Collect nasal absorptive matrix sample.
- Collect nasal mucosal scraping sample. If limited cell numbers are obtained from nasal scrapings, specimens from the same dose group/visit will be pooled by an unblinded member of the clinical team. Pooled specimens will be given a separate code. The coded specimens (pooled) will be processed by the lab personnel, who will remain blinded.

6.3.12 Visit 14: End of Study Call (Day 393 ± 7 days)

The final follow up for the study will be in the form of a telephone call 12 months following the subject's first dose. The following assessments will be completed:
- Review and update concomitant medications.
- Assess occurrence of any SAEs since prior visit.
- Record date and final disposition of the subject.

6.3.13 Early Termination Visit

In the event of the early termination of a subject, all attempts will be made to collect the assessments required at the current visit (if subject withdraws during a clinic visit) or at the next planned clinic visit (if subject withdraws between visits). Subjects will be encouraged to continue safety assessments per the study schedule through the end of the trial.

6.3.14 Use of left-over specimens after conduct of specified study assays

To participate in the study, participants will be required to consent to the use of biologic specimens that are left over from conducting the primary and secondary study assays. These specimens will be stored for potential future use.

6.3.15 Collection of specimens for future use

Certain biologic specimens will be collected that are not necessary for the evaluation of the primary and secondary study objectives. To participate in the study, participants will be required to consent to the collection and storage of these biologic specimens for potential future use. There will be no genetic testing done as part of this study or as part of any future use of clinical samples.

6.4 Unscheduled Study Visits

Unscheduled study visits will be scheduled for subjects to follow up on abnormal safety laboratory tests, medical complaints of concern for an AE, or to monitor documented AEs. The evaluation of the subjects for suspected AEs will be directed by the PI or appropriate Sub-Investigator per their clinical judgement. If there are clinically significant findings resulting in AE reports during any of the scheduled or unscheduled visits, the subject will be monitored for resolution of the symptoms with routine documented examinations. The medical evaluations conducted during these unscheduled visits will be dependent on the findings that resulted in their requirement, however targeted neurologic/otorhinoscopic/respiratory examination (Section 7.1.1) will be performed in all unscheduled study visits. Where possible, relevant data will be collected for unscheduled visits, i.e., labs, vitals, height and weight, physical and neurologic exams, etc.

6.5 Protocol Deviations

A protocol deviation is any noncompliance with the clinical trial protocol, GCP, or protocol-specific Manual of Operations requirements. The noncompliance may be either on the part of the subject, Investigator, or the site personnel. As a result of deviations, corrective actions are to be developed by the site and implemented promptly.

These practices are consistent with ICH E6:

- 4.5 Compliance with Protocol, Sections 4.5.1, 4.5.2, and 4.5.3
- 5.1 Quality Assurance and Quality Control, Section 5.1.1
- 5.20 Noncompliance, Sections 5.20.1, and 5.20.2.

It is the responsibility of the site PI and personnel to use continuous vigilance to identify and report deviations within five working days of identification of the protocol deviation, or within five working days of the scheduled protocol-required activity. All deviations must be promptly reported per federal regulations and NIH guidelines using protocol deviation reporting procedures.

All protocol deviations, as defined above, must be addressed in study subject data collection forms. A completed copy of the Protocol Deviation Form must be maintained in the Regulatory File, as well as in the subject's chart. Protocol deviations must be sent to the local IRB/IEC per their guidelines. The site PI and personnel are responsible for knowing and adhering to their federal regulations, NIH guidelines, and local IRB requirements.

7 DESCRIPTION OF CLINICAL AND LABORATORY EVALUATIONS

7.1 Clinical Evaluations

7.1.1 Clinical Evaluations and Research Procedures

- Medical History: All significant past and current diagnoses will be documented. All signs and symptoms, regardless of severity, that the subject has experienced within 30 days prior to screening, as well as medication allergies, should be documented. Medications prescribed for conditions not already captured in the Medical History should prompt a query about the reason for Drug, Alcohol and Smoking History. Information on historical and current use of alcohol and tobacco will be collected. Historical information on drug abuse will also be collected and a drug screening assessment performed by study clinician.
- Prior and Concomitant Medications: For any medication initiated during the study, the reason for starting the medication should be clear from either Medical History (for ongoing conditions) or from a reported adverse event. Thus, any medication prescribed during the study that is not for any ongoing condition reported in the Medical History, should prompt a query regarding an adverse event.
- Body Weight and Height: Weight and height will be collected at screening. Weight will also be collected on Days 1, 29, and 197, and when clinically indicated.
- Vital Signs: Vital signs include body temperature, blood pressure, heart rate, and respirations.
- Physical Examination: This protocol describes several different physical examination assessments. The following describes the different assessments and indicates when will be performed.
    - **Baseline physical examination** is an assessment of the general health status of the subject through examination of the cardiovascular, pulmonary, musculoskeletal, gastrointestinal, and dermatologic systems. The baseline physical examination is performed during the screening study visit and will assess the subject for any clinically significant abnormalities in the body systems noted.
    - **Directed physical exams** will be conducted on Days 1, 8, 29, 43, 57, 197, 204, and 225. The exam is directed to subjects' new complaints/symptoms that are considered change from base line physical exam. If a clinically significant abnormality or change in an existing abnormality occurs, the clinically significant finding should be reported as an adverse event and followed as directed in Section 8.
    - **Targeted neurologic/otorhinoscopic/respiratory examination**: This examination was developed by BlueWillow in partnership with both a neurologist and an ears/eyes/nose/throat specialist. The exam includes limited evaluation of extraocular movements, motor function of the facial muscles and sensory perception on the face, as well as assessment of mucosa, secretions, respiration, and some structural examinations. The examination also includes a respiratory evaluation. The list of

areas to be assessed is provided in Appendix C. The presence or absence of abnormalities will be assessed and recorded; if relevant, additional details may also be collected (e.g., mucosal color and texture). Targeted neurologic/otorhinoscopic/respiratory examinations will be performed at every clinic visit.

- Acute Reactogenicity Assessment: A specific list of signs and symptoms will be assessed with subject at 1-hour post-immunization for intranasal study vaccine and 30 minutes post-immunization for licensed Influenza Virus Vaccine, H5N1, before release from the clinic. The list of signs and symptoms to be evaluated for IN immunization are presented in Appendix D and for IM immunization in Appendix E. The grading system will be based on FDA toxicity grading scale for vaccines,[30] where:

Grade	Description
0 (None)	Not Present
1 (Mild)	Noticeable, but no interference with usual activities. Requires minimal or no treatment.
2 (Moderate)	May cause some interference with functioning and daily activities. Results in a low level of inconvenience or concern with therapeutic measures.
3 (Severe)	Interrupt daily activities and requires medical intervention. They are usually incapacitating.
4 (Potentially life-threatening)	Required ER visit or hospitalization
5 (Death)	Death

These data will contribute acute data to the overall assessment of reactogenicity.

- Symptom Diary: A Symptom Diary will be used by the subject to record signs and symptoms noted daily in the week following each immunization (IN and IM). Subjects will be instructed to record their temperature daily (after IN immunization) and will be asked to grade the severity of their symptoms daily using the same grading scale described in Appendix D (IN) or Appendix E (IM). The Symptom Diary will be collected as a non-source document at the next visit and used as a tool to enhance discussions with the subject, during which the Investigator or designee will complete the reactogenicity assessment at the clinic visit following an immunization visit. If the clinic visit is the same day as the final Symptom Diary entry, the final symptom assessment will be completed during the clinic visit. The symptoms to be assessed following IN vaccination are listed Appendix D. The symptoms for evaluation following IM immunization with H5N1 Influenza Vaccine are listed in Appendix E. An electronic Symptom Diary may be used. In this event, subjects will provide an email address or mobile phone number and agree to receive daily electronic messages directing them to a REDCAP survey that will be identical in language and format to a paper Symptom Diary. An electronic Symptom Diary would be used in the same way as a paper Symptom Diary – as a tool to enhance discussions between the subject and the Investigator or designee to complete the reactogenicity assessment following each immunization visit.
- Reactogenicity Assessment: An assessment of reactogenicity will be performed at each visit that follows an immunization visit, similar to the acute reactogenicity assessment made following each immunization. Utilizing the subject Symptom Diary, the Investigator or

designee will discuss signs and symptoms with the subject, identifying the presence or absence of each during the seven days following vaccination, and the maximum reported severity, if present. The symptoms to be assessed following IN vaccination are listed in Appendix D. The symptoms for evaluation following IM immunization with H5N1 Influenza Vaccine are listed in Appendix E.

- Follow up Safety Phone Calls: Subjects will be followed up with a phone call by the study personnel 2-4 days following each immunization to inquire about any symptoms. Subjects will be reminded to complete their symptom diary daily. On Day 393± 7 days, a phone call will be made by the study personnel to assess subject for SAEs and final end of study status.

7.1.2 Prior and Concomitant Medications/Treatments

All prescription and non-prescription medications, alternative therapies, and vaccines taken within 30 days prior to screening or since the last study visit should be documented. All concomitant medications, including contraceptives, vitamins and minerals should be documented during the study.

7.1.2.1 Prohibited Medications

The following types of medications (both prescription and over the counter) should not be used 7 days before or after each IN immunization:

- Nasally administered cold preparations (Dristan, Afrin, etc.)
- Nasally administered corticosteroids (Flonase, Nasonex, etc.)
- Any nasal spray

7.1.3 Assessment of Subject Compliance with Study Intervention/Investigational Product/Investigational Device

The investigational product will be administered by trained study personnel in the clinic. Compliance will be monitored and recorded by the study personnel documenting whether the subject received the complete volume of the vaccine dose or not. If a subject fails to receive the complete volume of the assigned dose, the number of drops or documentation of the difficulty with spray administration will be documented and the reason for the incomplete dose will be recorded onto the case report form (CRF).

7.2 Laboratory Evaluations

7.2.1 Clinical Laboratory Evaluations

A single laboratory will perform analysis of the following safety evaluations:

Hematology (screening and during study): hemoglobin, white blood cell count, and platelet count.

Biochemistry (screening and during study): creatinine, total bilirubin, alanine aminotransferase (ALT), and alkaline phosphatase.

Serology (screening): Hepatitis Bs (surface) antigen, Hepatitis C antibody, and HIV-1 and HIV-2 antibodies.

Pregnancy test (screening and during study): A serum pregnancy test (beta-HCG) will be conducted at screening. Prior to each vaccination, a urine pregnancy test will be conducted on all females of childbearing potential.

Other screening: Erythrocyte sedimentation rate (ESR).

COVID-19 testing: Molecular diagnostic-based qualitative analysis for evidence of SARS-CoV-2 infection.

7.2.2 Research Assays

7.2.2.1 Mucosal Immunogenicity Assays

Nasal wash specimens will be taken to evaluate for mucosal IgA, IgG, and HI, and mononuclear cells to evaluate T cell subset composition and functional properties. Certain aliquots of nasal wash specimens will go to the CVD Applied Immunology Laboratory for IgA, IgG analyses, and HI analyses, and CVD Cellular Immunology Lab for flow cytometry. Some specimens will be collected for future use only, as described in Appendix G Procedure Volumes.

7.2.2.2 Humoral Immunogenicity Assays

Blood samples will be taken to assess the levels of humoral HI titers, IgA, IgG, and Influenza A Group 1 antistem antibodies. These assays will be completed on samples obtained at baseline, post-intranasal vaccination and post H5N1 IM vaccination. Serum will be extracted from blood and aliquots will go to the CVD Applied Immunology Laboratory for IgA, IgG, HI analyses, and antistem IgG analyses. Some specimens will be collected for future use only, as described in Appendix G Procedure Volumes.

7.2.2.3 Cell-Mediated Immunogenicity Assays

Blood samples will be taken and peripheral blood mononuclear cells (PBMCs) isolated to assess the activation (e.g., CD69, HLA-DR), cytokine production and/or degranulation markers (e.g., IFN-γ, TNF-α, IL-2, IL-17A, CD107a) in T cell subsets upon rH5 ex-vivo stimulation. These assays will be completed on samples obtained at baseline, post-intranasal vaccination and post H5N1 IM vaccination to assess reactions to study treatments. These analyses will be done by the CVD Cellular Immunology Lab. Additionally, PBMC ELISpot analysis to quantitate the frequency of rH5-specific IgG and IgA producing memory B-cells will be conducted by the CVD Cellular Immunology Lab. Immunophenotypic determinations and/or T cell responses to rH5 in cells collected from nasal mucosal scrapings will be performed at the CVD Cellular Immunology Lab. Some specimens will be collected for future use only, as described in Appendix G Procedure Volumes. In the event of low cell yields from nasal wash or nasal mucosal scraping specimens, an unblinded study member who will not be involved in safety or otherwise involved in immunogenicity assessments will combine specimens by study arm and day for pooled analyses.

7.2.2.4 Laboratory Specimen Preparation, Handling, and Storage

Complete instructions for specimen preparation, handling and storage are provided in the Manual of Procedures.

7.2.2.5 Laboratory Specimen Shipping

Nasal washes, sera, saliva, nasal absorptive matrix, and PBMC from blood specimens will be prepared and stored at CVD.

8 ASSESSMENT OF SAFETY

8.1 Assessing and Recording Safety Parameters

Safety will be assessed in terms of the incidence of:

- Solicited reactogenicity reports (acute and within 7 days of each immunization),
- Unsolicited reports of adverse events (within 28 days of each immunization)
- Laboratory abnormalities (within 7 days of first dose BW-1014, within 14 days of second dose BW-1014, and within 7 days of parenteral H5N1 IIV vaccination)
- Medically attended adverse events (MAAEs) (across entire study period)
- New onset chronic medical conditions (NOCMCs) (across entire study period)
- Serious adverse events (across entire study period)
- Potentially immune-mediated medical conditions (PIMMCs) (across entire study period)

Severity of reactions and adverse events, as well as Investigator assessment of relatedness to treatment will also be evaluated.

8.1.1 Adverse Events (AEs)

ICH E6 defines an AE as any untoward medical occurrence in a patient or clinical investigation subject administered a pharmaceutical product regardless of its causal relationship to the study treatment. FDA defines an AE as any untoward medical occurrence associated with the use of a drug in humans, whether or not considered drug related.

An AE can therefore be any unfavorable and unintended sign (including an abnormal laboratory finding), symptom, or disease temporally associated with the use of medicinal (investigational) product. The occurrence of an AE may come to the attention of study personnel during study visits and interviews of a study recipient presenting for medical care, or upon review by a study monitor.

All AEs will be captured on the appropriate data collection form and eCRF. Information to be collected for AEs includes event description, date of onset, assessment of severity, relationship to study product and alternate etiology (assessed only by those with the training and authority to make a diagnosis and listed on the Form FDA 1572 as an investigator), date of resolution, seriousness and outcome. AEs occurring during the trial collection and reporting period will be documented appropriately regardless of relationship. AEs will be followed through resolution or until deemed stable by the Investigator.

Any medical condition that is present at the time that the subject is screened will be considered as baseline and not reported as an AE. However, if the severity of any pre-existing medical condition increases, it should be recorded as an AE.

Adverse events, which include abnormal and clinically significant clinical laboratory test variables, will be monitored from the time of first immunization until study participation is

complete (Week 56). Subjects should be instructed to report any adverse event that they experience to the study personnel. Any medical condition already present prior to the subject's initial immunization will be reported in the medical history. Should any changes in the subject's health occur between consent and first dose of study treatment, the study personnel will make corresponding updates to medical history.

Beginning with the first immunization, the study personnel will make an assessment for adverse events at each visit and record all adverse events, non-serious and serious, on the appropriate adverse event CRF. Any pre-existing medical condition or signs or symptoms that change adversely in severity, frequency, or seriousness (outside of the range of typical variation of the symptom over time in the past for the subject) after the subject receives the first immunization should be reported as an adverse event.

Wherever possible, a specific disease or syndrome rather than individual associated signs and symptoms should be identified by the study personnel and recorded on the CRF. However, if an observed or reported sign or symptom is not considered a component of a specific disease or syndrome by the study personnel, it should be recorded as a separate adverse event on the CRF. Additionally, the condition that led to a medical or surgical procedure (e.g., surgery, endoscopy, tooth extraction, or transfusion) should be recorded as an adverse event, not the procedure. Concomitant procedures should be recorded as such on the appropriate CRF.

Treatment of any adverse reactions will be evaluated and managed by the PI or appropriate Sub-Investigator.

8.1.1.1 Adverse Events Grading

All AEs (laboratory and clinical symptoms) will be graded for severity [30] and assessed for relationship to study product (see definitions). Appendix B contains the Toxicity Tables to be applied for the study. AEs characterized as intermittent require documentation of onset and duration of each episode. The start and stop date of each reported AE will be recorded on the appropriate data collection form and eCRF. Changes in the severity of an AE will be documented to allow an assessment of the duration of the event at each level of intensity.

For events not included in the protocol-defined grading system, the following guidelines will be used to quantify severity:

Mild (Grade 1): Events that are usually transient and may require only minimal or no treatment or therapeutic intervention and generally do not interfere with the subject's usual activities of daily living.

Moderate (Grade 2): Events that are usually alleviated with additional specific therapeutic intervention. The event interferes with usual activities of daily living, causing discomfort but poses no significant or permanent risk of harm to the research participant.

Severe (Grade 3): Events interrupt usual activities of daily living, or significantly affects clinical status, or may require intensive therapeutic intervention. Severe events are usually incapacitating.

Relationship to Study Product: The assessment of the AE's relationship to study product will be done by a clinician licensed to make medical diagnoses and listed on the Form FDA 1572 and the assessment will be part of the documentation process. Whether the AE is related or not is not a factor in determining what is or is not reported in this trial. If there is any doubt as to whether a clinical observation is an AE, the event should be reported.

In a clinical trial, the study product must always be suspect. The relationship to study product will be assessed for AEs using the terms related or not related:

Related – There is a reasonable possibility that the study product caused the AE. Reasonable possibility means that there is evidence to suggest a causal relationship between the study product and the AE.

Not Related – There is not a reasonable possibility that the administration of the study product caused the event.

8.1.2 Reactogenicity

Reactogenicity events are AEs that are common and known to occur following administration of this type of study vaccine. The reactogenicity grading scales for IN administration of vaccine are provided in Appendix D, and the reactogenicity grading scales for IM administration of vaccine are provided in Appendix E.

8.1.3 Serious Adverse Events (SAEs)

At all contacts, the subject will be queried and assessed for serious adverse events (SAEs), potentially immune- mediated medical conditions (PIMMCs, see Appendix F). An adverse event or suspected adverse reaction is considered "serious" if, in the view of the Investigator, it results in any of the following outcomes:

- Death,
- a life-threatening adverse event,
- inpatient hospitalization or prolongation of existing hospitalization,
- a persistent or significant incapacity or substantial disruption of the ability to conduct normal life functions, or
- a congenital anomaly/birth defect.

Important medical events that may not result in death, be life-threatening, or require hospitalizations may be considered serious when, based upon appropriate medical judgment they may jeopardize the patient or subject and may require medical or surgical intervention to prevent one of the outcomes listed in this definition. Examples of such medical events include allergic bronchospasm requiring intensive treatment in an emergency room or at home, blood dyscrasias or convulsions that do not result in inpatient hospitalization, or the development of drug dependency or drug abuse.

An adverse event is considered “life-threatening” if, in the view of either the Investigator, its occurrence places the patient or subject at immediate risk of death. It does not include an adverse event, had it occurred in a more severe form, might have caused death.

For serious adverse events, the reporting period to BlueWillow Biologics or its designated representative begins from the time that the subject provides informed consent through and including the follow-up period (for this study approximately 12 months after the last dose of the investigational product).

SAEs will be:

- Assessed for severity and relationship to study product and alternate etiology (if not related to study product) by the PI or appropriate Sub-Investigator.
- Recorded on the appropriate SAE data collection form and eCRF.
- Followed through resolution by the PI or appropriate Sub-Investigator .
- Reviewed and evaluated by DMID, the independent Safety Monitoring Committee (SMC; periodic review unless related), and the IRB/IEC (as per IRB requirements).

8.2 Specification of Safety Parameters

The following are the safety parameters to be assessed:

- Solicited reactogenicity reports (within 7 days of each immunization),
- Unsolicited reports of adverse events (within 28 days of each immunization)
- Laboratory abnormalities (within 7 days of first dose BW-1014, within 14 days of second dose BW-1014, and within 7 days of parenteral H5N1 IIV vaccination)
- Medically attended adverse events (MAAEs) (across entire study period)
- New onset chronic medical conditions (NOCMCs) (across entire study period)
- Serious adverse events (across entire study period)
- Potentially immune-mediated medical conditions (PIMMCs) (across entire study period)

8.2.1 Solicited Events

The solicited events in this study are the reactogenicity assessments. For acute reactogenicity, subjects will be assessed in the clinic following each immunization, 1 hour following IN vaccination and 30 minutes following IM vaccination, prior to release. Subject will then be discharged with a Symptom Diary that will list the solicited events to be aware of and record notes to discuss with the study personnel at the clinic visit one week after immunization; the subject may also note any other events they experience for discussion with the study personnel (unsolicited events).

8.2.2 Unsolicited Events

Unsolicited events are any other AEs that occur following administration of study. This will be collected via discussions between the PI and subject at each clinic visit, observations by the

Investigator and reported as abnormal laboratory test results (Grade 1 or higher, as reported by laboratory).

Any post-dose laboratory parameter that is a Grade 2 or greater will require the subject to return to clinic for retesting. The Investigator will evaluate whether this change in the laboratory test result has a clinical significance and define the cause and grade it according to AEs/SAEs grading. Any AE or SAEs should be reported accordingly. The subject will be followed until the value(s) return to pre-dose levels or is at a level that is acceptable to the Investigator.

8.2.3 New-Onset Chronic Medical Conditions (NOCMCs)

NOCMCs are defined as any new ICD-10 diagnosis that is applied to the subject during the duration of the study, after receipt of the study agent, that is expected to continue for at least 3 months and requires continued health care intervention.

8.2.4 Medically-Attended Adverse Events (MAAEs)

For each unsolicited AE experienced, the subject will be asked if he/she had received medical attention, defined as hospitalization, an ER visit, or an otherwise unscheduled visit to or from medical personnel for any reason. AEs characterized by such unscheduled medical care will be designated as MAAEs.

8.2.5 Potentially Immune-Mediated Medical Conditions (PIMMCs)

PIMMCs constitute a group of AEs that includes diseases which are clearly autoimmune in etiology and other inflammatory and/or neurologic disorders which may or may not have autoimmune etiologies. PIMMCs currently in effect are presented in Appendix F.

8.3 Reporting Procedures

Adverse events will be collected from the day of first study immunization (Day 1) through Day 225 (28 days following IM boost vaccination). Serious adverse events will be collected from the day of first study immunization (Day1) through Day 393 (end of study).

8.3.1 Reporting Serious Adverse Events

From the time of informed consent until Day 393, the Investigator or designee will notify the SMC membership (including observers) by email within 24 hours after the SAE detection, observation, or report of occurrence (regardless of the relationship to study treatment).

SAEs will be followed until resolution even if this extends beyond the study-reporting period. Resolution of an AE is defined as the return to pretreatment status or stabilization of the condition with the expectation that it will remain chronic.

Any AE that meets a protocol-defined serious criterion must be submitted immediately (within 24 hours of site awareness) on an SAE form to the DMID Medical Monitor, Medical Officer, and Clinical Project Manager. In addition to the SAE form, select SAE data

fields must also be entered into the data collection system for the clinical trial. Please see the protocol-specific MOP for details regarding this procedure.

Other supporting documentation of the event may be requested by the DMID and should be provided as soon as possible.

The site will send a copy of the SAE report(s) to the SMC (as deemed necessary) when they are provided to the DMID. The DMID Medical Monitor will review and assess the SAE and potential impact on study subject safety and protocol conduct.

At any time after completion of the study, if the site PI or appropriate Sub-Investigator becomes aware of an SAE that is suspected to be related to study product, the site PI or appropriate Sub-Investigator will report the event to the DMID.

8.3.2 Regulatory Reporting for Studies Not Conducted Under DMID Sponsored IND

The IND sponsor will review, assess, and report events that are both serious and unexpected that are related to study product(s) to the FDA within the required timelines as specified in 21 CFR Part 312.32 and provide a copy of the regulatory submission to DMID: fatal and life-threatening events within 7 calendar days (by telephone or fax). All written reports will be sent to the FDA within 15 calendar days. All serious events designated as “not related” to study product(s), will be reported to the FDA at least annually in a summary format.

Reporting to the NIH will follow NIH specified requirements and government regulations.

8.3.3 Reporting of Pregnancy

If any female study subject becomes pregnant during their study participation, the Investigator must complete a pregnancy notification form and submit to the SMC. The information submitted should include the anticipated date of delivery. Any further immunizations prescribed by the protocol should NOT occur.

The Investigator will be required to follow the pregnancy until completion or until pregnancy termination. If the pregnancy is terminated by induced abortion, the reasons for an induced abortion should be reported.

8.4 Type and Duration of Follow-up of Subjects after Adverse Events

Adverse events assessed as treatment-related will be followed until resolved or considered stable according to the judgment of the Investigator, up through the final clinic visit (Day 225, or 1 year from last immunization for subjects who terminate early). Treatment-related SAEs will be followed until resolution even if this extends beyond Day 393. Resolution of an AE is defined as the return to pretreatment status or stabilization of the condition with the expectation that it will remain chronic.

8.5 Procedures to be Followed in the Event of Abnormal Laboratory Test Values or Abnormal Clinical Findings

Safety laboratory results will be reported as AEs if they are assessed as a Grade 1 or higher results by the central laboratory. An abnormal laboratory result that is not verified by repeat testing does not necessitate reporting as an adverse event. The Investigator will exercise his or her medical, scientific, and clinical judgment in deciding whether an abnormal laboratory finding, or other abnormal assessment is clinically significant. Clinically significant abnormal laboratory values occurring during the clinical study will be followed until repeat tests return to normal, stabilize, or are no longer clinically significant. Any abnormal test that is determined to be an error does not require reporting as an adverse event.

8.6 Halting Rules

8.6.1 Study Halting Criteria

If any of the following halting rules are met, vaccinations will be suspended until after a review by the SMC:

- Any death or SAE for which causality is unknown or related to vaccination.
- If one or more subjects experience the same or similar Grade 3 or higher AE or laboratory abnormality rated as severe (Grade 3 or higher) with an underlaying clinical significance which are related to investigational vaccine.

These criteria will also be used by the SMC in its review of safety data at Day 7 review after sentinel participant vaccination. If the SMC concurs that the halting criteria were met, vaccination will be suspended.

8.6.2 Individual Halting Criteria

Individual dosing will be suspended for any subject with the following:

- A Grade 3 or higher adverse event (AE) until the AE is resolved and it has been determined that the event was not related to study product administration.
- Occurrence of any treatment-related serious adverse event (SAE) or Grade 3 or 4 adverse event (AE) following a preceding vaccination
- Individuals with fever >100.4˚ F within one week prior to any immunization require a temporary delay in dosing until fever resolves.
- Positive pregnancy test on the day of vaccination (female participants).
- New onset of illness or condition (including any evidence or strong suspicion of COVID-19) that meets the Subject Exclusion Criteria (see Section 5.1.2).Presence of signs or symptoms that could confound or confuse assessment of study vaccine reactogenicity. For subjects with an acute illness, including an oral temperature greater than or equal to 100.4°F, the second study vaccination should be postponed/deferred until signs, symptoms, or acute illness have resolved, or are improving as further specified below,

and if within the acceptable protocol-specified window for that visit. **Note for afebrile, acute illness only**: If a subject is afebrile, his/her acute illness is nearly resolved with only minor residual symptoms remaining, this occurs within the acceptable protocol-specified window for that visit, and, in the opinion of the PI or appropriate Sub-Investigator, the residual symptoms will not interfere with the ability to assess safety parameters as required by the protocol, the subject may receive the study vaccination.

- As deemed necessary by the PI or appropriate Sub-Investigator for noncompliance or other reasons.
- Subject refusal of further study vaccination.
- Subject withdrawal of consent.
- Subject lost to follow-up.
- Termination of this trial.
- New information becomes available that makes further participation unsafe.

8.7 Safety Oversight

8.7.1 Safety Monitoring Committee (SMC)

An independent Safety Monitoring Committee (SMC) will be responsible for the oversight of safety for this study. The SMC will be organized by the study Principal Investigator. The SMC will consist of up to three scientists that are not involved with the conduct of the study. The primary responsibility of the SMC is to monitor participant safety. Non-voting representatives of the sponsor and vaccine manufacturers will also participate in the SMC, as will the unblinded study biostatistician who will be available to assist in the event of unblinding or the need for interpreting the statistical results as needed. The SMC considers study-specific data as well as relevant background information about the disease, test agent, and target population under study. The SMC will review the following:

- sentinel subjects' safety data from each Cohort through seven days after their initial vaccine dose
- cumulative safety data shortly after completion of the Day 57 visit for all subjects (28 days following IN vaccine administration)
- cumulative safety data at the end of study

The SMC will be empowered to do any/all of the following:

- recommend proceeding with full study group vaccination upon review of sentinel subject safety data
- suspend (halt) the study
- recommend amendments to the protocol
- request further information for their review

Should there be a halt in the study, no further study vaccinations will be performed until the halt is lifted; all enrolled participants will continue to be followed for safety. An SMC Charter will be

reviewed and approved by the SMC members, the PI and DMID prior to the initiation of the trial and will include the scheduled frequency/timing of SMC meetings, types of data for review, halting rules, and roles/responsibilities.

The PI will convene the SMC at specified times during the course of study as defined in the SMC Charter and on an ad hoc basis according to protocol criteria or if there are immediate concerns regarding observations during the course of the study.

9 HUMAN SUBJECTS PROTECTION

9.1 Institutional Review Board/Independent Ethics Committee

A single research site will be involved in this study. The Investigator will obtain IRB approval for this protocol to be conducted at his/her research site and send supporting documentation to the DMID before initiating recruitment of subjects. The Investigator will submit applicable information to the IRB/IEC on which it relies for the review, to conduct the review in accordance with 45 CFR 46, ICH E6 GCP, and as applicable, 21 CFR 56 (Institutional Review Boards) and 21 CFR 50 (Protection of Human Subjects), other federal, state, and local regulations. The IRB/IEC must be registered with OHRP as applicable to the research. DMID must receive the documentation that verifies IRB/IEC-approval for this protocol, associated informed consent documents, and upon request any recruitment material and handouts or surveys intended for the subjects, prior to the recruitment and enrollment of subjects.

Any amendments to the protocol or consent materials will be approved by the IRB/IEC before they are implemented. IRB/IEC review and approval will occur at least annually throughout the enrollment and follow-up of subjects and may cease if annual review is no longer required by applicable regulations and the IRB/IEC. The Investigator will notify the IRB/IEC of deviations from the protocol and reportable SAEs, as applicable to the IRB/IEC policy.

Each institution engaged in this research will hold a current Federalwide Assurance (FWA) issued by the Office of Human Research Protection (OHRP) for federally funded research.

9.2 Informed Consent Process

Informed consent is a process that is initiated prior to an individual agreeing to participate in a trial and continuing throughout the individual’s trial participation. Before any study procedures are performed, informed consent will be obtained and documented. Subjects will receive a concise and focused presentation of key information about the clinical trial, verbally and with a written consent form. The explanation will be organized and presented in lay terminology and language that facilitates understanding why one might or might not want to participate.

The Investigator or designee will describe the protocol to potential subjects face-to-face. The key information about the purpose of the study, the procedures and experimental aspects of the study, risks and discomforts, any expected benefits to the subject, and alternative treatment will be presented first to the subject.

Subjects will also receive an explanation that the trial involves research and a detailed summary of the proposed study procedures and study interventions/products. Subjects will be asked to consent to the future use of left-over specimens and to future use of specimens collected at timepoints that will not be used to evaluate the primary or secondary study objectives. If they choose to not provide permission for left-over specimens or specimens collected for future use,

they will not be eligible for randomization and enrollment into the study. This will include aspects of the trial that are experimental, the probability for random assignment to treatment groups, any expected benefits, all possible risks (including a statement that the particular treatment or procedure may involve risks to the subject or to the embryo or fetus, if the subject is or may become pregnant, that are currently unforeseeable), the expected duration of the subject’s participation in the trial, alternative procedures that may be available and the important potential benefits and risks of these available alternative procedures.

Subjects will be informed that they will be notified in a timely manner if information becomes available that may be relevant to their willingness to continue participation in the trial. Subjects will receive an explanation as to whether any compensation and any medical treatments are available if injury occurs, and, if so, what they consist of, or where further information may be obtained. Subjects will be informed of the anticipated financial expenses, if any, to the subject for participating in the trial, as well as any anticipated prorated payments, if any, to the subject for participating in the trial. They will be informed of whom to contact (e.g., the Investigator) for answers to any questions relating to the research project.

Information will also include the foreseeable circumstances and/or reasons under which the subject’s participation in the trial may be terminated. The subjects will be informed that participation is voluntary and that they are free to withdraw from the study for any reason at any time without penalty or loss of benefits to which the subject is otherwise entitled.

The extent of the confidentiality of the subjects’ records will be defined, and subjects will be informed that applicable data protection legislation will be followed. Subjects will be informed that the monitor(s), auditors(s), IRB, NIAID, and regulatory authority(ies) will be granted direct access to the subject’s original medical records for verification of clinical trial procedures and/or data without violating the confidentiality of the subject, to the extent permitted by the applicable laws and regulations, and that, by signing a written informed consent form, the subject is authorizing such access.

Subjects will be informed that records identifying the subject will be kept confidential, and, to the extent permitted by the applicable laws and/or regulations, will not be made publicly available and, if the results of the trial are published, the subject’s identity will remain confidential. Subjects will be informed whether private information collected from this research and/or specimens will be used for additional research, even if identifiers are removed.

Subjects will be allowed sufficient time to consider participation in this research trial and have the opportunity to discuss this trial with their family, friends or legally authorized representative, or think about it prior to agreeing to participate.

Informed consent forms will be IRB-approved, and subjects will be asked to read and review the consent form. Physical (paper) informed consent forms (ICF) or electronic informed consent form (eICF) may be used. To facilitate an eICF process, the IRB-approved ICF will be entered into the study REDCap database. Subjects will be asked to read and review the consent form via

a tablet or laptop. Hard copies will also be available for those who would prefer to review a physical version of the consent form.

The eICF and hard copies will be identical in language and format. Subjects must sign the informed consent form prior to starting any study procedures being done specifically for this trial. Subject signatures will be captured electronically via REDCap’s electronic signature field using either a stylus or their finger. In addition to the REDCap system timestamp on the electronic signature, date and printed (typed) name fields will also be completed by the subject. All eICFs will be e-signed in the presence of the Investigator or designee. In the event that the REDCap system is unavailable the study team will revert to use of a paper ICF, a copy of which will be uploaded to the study REDCap database.

Once electronically signed, a pdf copy of the informed consent form is available through the REDCap system and can be downloaded and either printed, handed to the subject, or emailed to the subject for their records.

The subject(s) may withdraw consent at any time throughout the course of the trial. The rights and welfare of the subject(s) will be protected by emphasizing to them that the quality of their medical care will not be adversely affected if they decline to participate in this study.

Study personnel may employ recruitment efforts prior to obtaining study consent if a patient-specific screening consent is on record or if the IRB has agreed that chart review is allowed without a fully executed screening consent. In cases where there is not a patient-specific screening consent on record, site Clinical staff may pre-screen via chart review and refer potential subjects to the Research staff. Research staff would obtain written consent per the standard informed consent process before conducting protocol-specific screening activities.

New information will be communicated by the site PI to subjects who consent to participate in this trial in accordance with IRB requirements. The informed consent document will be updated, and subjects will be re-consented per IRB requirements, if necessary. Subjects will be given a copy of all informed consent forms that they sign.

9.2.1 Exclusion of Women, Minorities, and Children (Special Populations)

The enrollment of study participants, including women and minorities, is not targeted for specific populations and generally reflects the general population of the local Baltimore community and University campus. The inclusion and exclusion criteria were designed to screen for “health” and there is no selection according to gender, race, nor ethnicity. Persons with significant underlying diseases, which may affect immune responses, are intended to be excluded from study participation. Recruitment of study participants is performed by engaging with local schools and by flyers and word-of-mouth; no specific populations are targeted.

Persons <18 years of age or >45 years of age are not intended to participate in this study.

9.3 Subject Confidentiality

Every effort is made to protect the confidentiality of the study records. Subject confidentiality is strictly held in trust by the participating investigators and staff, the sponsor(s) and their agents; required NIH Certificate of Confidentiality is inclusive. Subjects have code numbers and are not identified by name. Records are maintained in locked cabinets, and access is limited to authorized personnel. This confidentiality extends to biological sample tests, and to the clinical information relating to participating subjects. No information concerning the study or the data will be released to any unauthorized third party without the prior written approval of the sponsor. The study monitor or other authorized representatives of the sponsor may inspect all documents and records required to be maintained by the Investigator such as medical records (office, clinic, or hospital). Record management complies with HIPAA regulations.

All computer entry and networking programs will be carried out with coded numbers only and with password protected systems. All non-clinical specimens, evaluation forms, reports, and other records that leave the site will be identified only by a coded number.

9.4 Certificate of Confidentiality

To protect privacy, we have received a Certificate of Confidentiality. With this Certificate, the researchers cannot be forced to release information that may identify the research subject, even by a court subpoena, in any federal, state, or local civil, criminal, administrative, legislative, or other proceedings. The researchers will use the Certificate to resist any demands for information that would identify the subject, except as explained below.

The Certificate cannot be used to resist a demand for information from personnel of the United States Government that is used for auditing or evaluation of federally funded projects, like this study, or for information that must be released in order to meet the requirements of the FDA.

A Certificate of Confidentiality does not prevent the subject from voluntarily releasing information about themselves or their involvement in this research. If any person or agency obtains a written consent to receive research information, then the researchers may not use the Certificate to withhold that information.

The Certificate of Confidentiality does not prevent the researchers from reporting without the subject’s consent, information that would identify the subject as a participant in the research project regarding matters that must be legally reported, including child and elder abuse, sexual abuse, or wanting to harm themselves or others.

The release of individual private information or specimens for other research will only occur if consent was obtained from the individual to whom the information, document, or biospecimen pertains, or for the purposes of other research that is in compliance with applicable Federal regulations governing the protection of human subjects in research.

9.5 Costs, Subject Compensation, and Research Related Injuries

There is no cost to subjects for the research tests, procedures, and study product while taking part in this trial. Procedures and treatment for clinical care may be billed to the subject, subject's insurance or third party. Subjects may be compensated for their participation in this trial. Compensation will be in accordance with the local IRB's policies and procedures, and subject to IRB approval.

If a subject suffers physical injury from this study, the study doctor will provide immediate medical treatment. The study doctor will also provide referrals to appropriate health care facilities. In general, no long-term medical care or financial compensation for research-related injuries will be provided by the NIH or the Federal Government. No financial compensation will be made for any discomfort suffered because of participation in this study. However, by signing this consent form the subject does not give up any of the legal rights you have as a participant in a research study.

10 STATISTICAL CONSIDERATIONS

The objectives of this study are to describe the safety and immunologic response of a novel influenza vaccine candidate used for the first time in humans. In this Phase I clinical trial of healthy adult volunteers, all analyses are exploratory in nature. The primary analysis population for both safety and immunology evaluations will be all subjects having received at least an initial dose of study treatment.

10.1 Study Hypotheses

There are no hypothesis tests pre-specified for this Phase I study, although some inferential statistics such as 95% confidence levels will be calculated to supplement the descriptive statistics provided for outcome measures.

10.2 Sample Size Considerations

The sample size is chosen without the intention that group differences will be detected with a sufficient power but is consistent with FDA guidance. A sample size of 40 with 8 persons per vaccine group is selected.

10.3 Treatment Assignment Procedures

10.3.1 Randomization Procedures

Upon verification of study eligibility at the Day 1 visit, subjects will be randomized according to Table 2. Sentinel Subjects and Dose Escalation Schedule

. Because this is a dose escalation trial, our study has four stages with one cohort receiving vaccines at each stage. Up to 40 participants will be randomized to one of the five study groups at an allocation ratio depending on the escalation stage. If all participants proceed to vaccination, the final vaccine allocation ratio will be 1:1:1:1:1. Subjects will receive a primary series of two intranasal vaccinations of study treatment administered on Days 1 and 29. Subject dosing will proceed in a stepwise process. Each dose of adjuvanted study vaccine will be assessed in sentinel participants before the remainder of the study group is vaccinated and before proceeding to vaccination of sentinel participants with the next higher dose of adjuvanted study vaccine. Randomization will be generated by the study statistician using block randomization for each cohort in Table 2 separately.

10.3.2 Masking Procedures

The vaccines containing nanoemulsion adjuvant are milky white in appearance, while the active control and placebo are clear in appearance. Therefore, there is potential that vaccinators or subjects can be unblinded by the vaccine presentation. The investigational product compounding

facility will provide formulated product to the designated unblinded study personnel (nurses and/or clinician sub-investigators)for administration. Only the investigational product compounding facility personnel, designated study personnel, and the study biostatistician will have access to the randomization code prior to study completion. Designated unblinded study personnel will not be involved in safety or immunogenicity assessments. The study will remain blinded until the completion of all clinical visits and laboratory data are available for all primary and secondary endpoints. If, however, there is a delay in a laboratory assay for any secondary immunogenicity endpoint, the sponsor may unblind without all laboratory data being available and after database lock for the purpose of early dissemination in response to a public heath need. In this circumstance, the laboratory personnel responsible for completing the delayed assays will remain blinded to subject vaccine allocation until the assays are completed.

10.4 Planned Interim Analyses

10.4.1 Sentinel Subject Safety Review

The SMC will meet after completion of Day 7 visit for each of Cohorts 1-3 (Table 2. Sentinel Subjects and Dose Escalation Schedule

) to review the sentinel subjects' safety data collected and to assess whether study halting criteria (Section 8.6.1) are met. If no halting criteria are met, the SMC will allow proceeding to vaccination of the following Cohort, and/or to make additional recommendations as described in Section 8.7.1 and per the SMC charter.

10.4.2 Interim Safety Review

There are no formal interim safety analyses planned for this study, except for the SMC review of safety data (see Section 8.7.1). The SMC charter will delineate the data that will be provided for safety reviews and the timing of reviews and will be included in the Manual of Operations.

10.4.3 Immunogenicity Analysis

Analyses of immunology objectives will be undertaken after last in-person study visit. No immunogenicity data will be collected at the last study visit (which is a telephone call). The study biostatistician will have access to unblinded, individual level data, will complete analyses, and will make results available as aggregated, summary data by study arm and timepoint according to the Analysis Plan to the study team, sponsor, BARDA, and vaccine manufacturers. Investigators (with the exception of the biostatistician and designated unblinded study personnel participating in vaccine administration) will remain blinded at this stage and will only be unblinded after study completion.

As noted previously, in the event of low cell yields from nasal wash or nasal mucosal scraping specimens, pooled analyses may be undertaken which combine specimens by study arm and study day for certain flow cytometry assessments.

10.4.4 Analysis Plan

All subjects receiving at least one dose of study treatment will be included in the analyses. Adverse Events (AEs) will be tabulated for each treatment arm and for all BW-1014 vaccine recipients combined. Serious Adverse Events (SAEs) will be summarized separately. The frequency and severity of systemic and local reactions reported via Symptom Diary after each vaccination will be tallied for each treatment group and for all BW-1014 vaccine recipients combined. An additional table will tally symptoms of moderate severity or higher. All AEs and results of regularly scheduled lab tests will be presented by subject listings.

Descriptive summary statistics will be provided for all assays at each time point. For binary outcomes such as presence/absence of an AE or seroprotection (Yes/No), proportion will be calculated for each group with the corresponding 95% confidence intervals. At each time point of interest, the difference in proportions between the treatment arms will be compared using Fisher's exact test or Chi-square test as appropriate. For continuous outcomes, such as antibody titer or percentage of a particular cell subset expressing a response to antigenic stimulation, mean, standard deviation, median, interquartile range, and geometric mean with the corresponding 95% confidence intervals will be calculated for each group. At each time point, the difference in continuous outcomes of interest between the treatment groups will be compared using Kruskal-Wallis H-test or ANOVA F-test on the log-transformed (base 10) scale as appropriate. Pairwise comparison will be conducted if needed. All analyses will be performed using Stata/SE version 17 (Stata Corp, College Station, TX).

11 SOURCE DOCUMENTS AND ACCESS TO SOURCE DATA/DOCUMENTS

Laboratory reports received from study laboratories will be printed/reviewed/signed/dated, then subject-specific information is entered into the data capture system; the laboratory reports are considered to be source documents. Source document templates may be created for study data; these will be included in the Manual of Procedures. Printed case report forms may also be used as source documents in certain circumstances; these will be identified in the Manual of Procedures.

The research site will maintain appropriate medical and research records in compliance with ICH E6, Section 4.9 and regulatory and institutional requirements for the protection of confidentiality of subjects. The site will permit authorized representatives of the DMID, its designees, and appropriate regulatory agencies to examine (and when required by applicable law, to copy) clinical records for the purposes of quality assurance reviews, audits, and evaluation of the study safety and progress. These representatives will be permitted access to all source data and source documents, which include, but are not limited to, hospital records, clinical and office charts, laboratory notes, memoranda, subjects' symptom diary or evaluation checklists, drug dispensing records, recorded data from automated instruments, copies or transcriptions certified after verification as being accurate and complete, microfiches, photographic negatives, microfilm or magnetic media, x-rays, and subject files and records kept at the investigational product compounding facility, at the laboratories, and medico-technical departments involved in the clinical trial.

12 QUALITY CONTROL AND QUALITY ASSURANCE

Following a written and approved quality management plan, designated site personnel will be responsible for conducting routine quality assurance (QA) and quality control (QC) activities to internally monitor study progress and protocol compliance. The site principal investigator will provide direct access to all study-related source data/data collection forms, and reports for the purpose of monitoring and/or auditing by the sponsor, and inspection by local and regulatory authorities. The principal investigator will ensure all study personnel are appropriately trained and training documentation is current and maintained on site. Designated site personnel will implement QC and QA procedures beginning at screening which will continue through to study completion.

13 DATA HANDLING AND RECORD KEEPING

13.1 Data Management Responsibilities

The Investigator is responsible to ensure the accuracy, completeness, legibility, and timeliness of the data reported. All source documents should be completed in a neat, legible manner to ensure accurate interpretation of data. Black or blue permanent ink is required to ensure clarity of reproduced copies. When making changes or corrections, cross out the original entry with a single line, and initial and date the change. DO NOT ERASE, OVERWRITE, OR USE CORRECTION FLUID OR TAPE ON THE ORIGINAL.

13.2 Data Coordinating Center/Biostatistician Responsibilities

Data collection is the responsibility of the study personnel at the participating clinical study site under the supervision of the Investigator. During the study, the Investigator must maintain complete and accurate documentation for the study. The data coordinating center for this study will be responsible for data management, quality review, analysis, and reporting of the study data.

Quality control audits of all key safety, laboratory, and clinical data in the database will be made after data entry has been completed. Coexistent medical conditions, adverse events and other medical events will be coded using the MedDRA dictionary. Concomitant medications will be coded using the WHO-DD dictionary. When the database has been declared to be complete and accurate, the database will be locked. Any changes to the database after that time will only be by joint written agreement of the study team.

At the end of the study, a copy of all datasets will be provided to DMID.

13.3 Data Capture Methods

Data for this trial will include clinical, safety, and outcome measures (e.g., clinical laboratory values, reactogenicity, and immunogenicity data). This study will utilize direct data entry. Source data will be entered into a REDCap data management system, managed by the Clinical and Translational Research Informatics Center (CTRIC), University of Maryland School of Medicine. REDCap is a secure web-based application for building an electronic database that provides a 21 CFR Part 11, FISMA, and HIPAA-compliant environment. Quality control audits of all key safety, laboratory, and clinical data in the database will be made after data entry has been completed.

13.4 Study Records Retention

Study records and reports including, but not limited to, eCRFs, source documents, ICFs, laboratory test results, and study drug disposition records will be retained for 2 years after a

marketing application is approved for the study product for the indication for which it is being investigated; or, if no application is to be filed or if the application is not approved for the study product, until 2 years after the investigation is discontinued and the FDA has been notified. These documents will be retained for a longer period, however, if required by local regulations.

No records will be destroyed without the written consent of the Investigator. It is the responsibility of the Investigator to determine when these documents no longer need to be retained. The site must contact NIH/NIAID for authorization prior to the destruction of any study records.

14 CLINICAL MONITORING

Site monitoring is conducted to ensure that the rights and well-being of human subjects are protected, that the reported trial data are accurate, complete, and verifiable, and that the conduct of the trial is in compliance with the currently approved protocol/amendment(s), with GCP, and with applicable regulatory requirement(s). Monitoring refers to the methods used by sponsors of investigational studies, or CROs delegated site monitoring responsibilities, to oversee the conduct of, and reporting of data from, clinical investigations. Site monitoring includes ensuring appropriate clinical investigator supervision of study site staff and third-party contractors.

Site monitoring will be conducted to ensure that human subject protections, study procedures, laboratory procedures, study intervention administration, and data collection processes are of high quality and meet sponsor, GCP/ICH, and regulatory guidelines, and that the study is conducted in accordance with the protocol and sponsor standard operating procedures. DMID, the sponsoring agency, or its designee may also conduct site-monitoring visits as necessary.

Site visits are anticipated to be made at standard intervals. Monitoring visits will include, but are not limited to, review of regulatory files, accountability records, eCRFs, informed consent forms, medical and laboratory reports, and protocol compliance. Site monitors will have reasonable access to the study site, study personnel, and all study documentation. Study monitors will meet with site investigators to discuss any problems and actions to be taken and document visit findings and discussions. A CMP will be written, approved, and implemented by the coordinating center or DMID prior to the initiation of the study.

15 PUBLICATION POLICY

Following completion of the study, the Investigator is expected to publish the results of this research in a scientific journal. All investigators funded by the NIH must submit or have submitted for them to the National Library of Medicine's PubMed Central (http://www.ncbi.nlm.nih.gov/pmc/) an electronic version of their final, peer-reviewed manuscripts upon acceptance for publication, to be made publicly available no later than 12 months after the official date of publication. The NIH Public Access Policy ensures the public has access to the published results of NIH funded research. It requires investigators to submit final peer-reviewed journal manuscripts that arise from NIH funds to the digital archive PubMed Central upon acceptance for publication. Further, the policy stipulates that these papers must be accessible to the public on PubMed Central no later than 12 months after publication.

As of January 2018, all clinical trials supported by the NIH must be registered on ClinicalTrials.gov, no later than 21 days after the enrollment of the first subject. Results of all clinical trials supported by the NIH, generally, need to be submitted no later than 12 months following the primary completion date. A delay of up to 2 years is available for trials that meet certain criteria and have applied for certification of delayed posting.

As part of the result posting a copy of this protocol (and its amendments) and a copy of the Statistical Analysis Plan will be posted on ClincialTrials.gov.

For this trial the Investigator is the responsible party for registration of the trial and posting results.

The responsible party does not plan to request certification of delayed posting.

16 LITERATURE REFERENCES

1. Stanberry LR, Simon JK, Johnson C, et al. Safety and immunogenicity of a novel nanoemulsion mucosal adjuvant W805EC combined with approved seasonal influenza antigens. *Vaccine*. Jan 5 2012;30(2):307-16. doi:10.1016/j.vaccine.2011.10.094
2. Chichester JA, Jones RM, Green BJ, et al. Safety and immunogenicity of a plant-produced recombinant hemagglutinin-based influenza vaccine (HAI-05) derived from A/Indonesia/05/2005 (H5N1) influenza virus: a phase 1 randomized, double-blind, placebo-controlled, dose-escalation study in healthy adults. *Viruses*. Nov 19 2012;4(11):3227-44. doi:10.3390/v4113227
3. Belshe RB. The origins of pandemic influenza--lessons from the 1918 virus. *N Engl J Med*. Nov 24 2005;353(21):2209-11. doi:10.1056/NEJMp058281
4. Beigel JH, Farrar J, Han AM, et al. Avian influenza A (H5N1) infection in humans. *N Engl J Med*. Sep 29 2005;353(13):1374-85. doi:10.1056/NEJMra052211
5. McCullers JA. Preparing for the next influenza pandemic. *Pediatr Infect Dis J*. Oct 2008;27(10 Suppl):S57-9. doi:10.1097/INF.0b013e3181684d41
6. World Health Organization. Vaccines against influenza WHO position paper - November 2012. *Wkly Epidemiol Rec*. Nov 23 2012;87(47):461-76.
7. Morens DM, Taubenberger JK, Fauci AS. The persistent legacy of the 1918 influenza virus. *N Engl J Med*. Jul 16 2009;361(3):225-9. doi:10.1056/NEJMp0904819
8. World Health Organization. Cumulative number of confirmed human cases for avian influenza A(H5N1) reported to WHO, 2003-2018. World Health Organization. Accessed 7 December 2018, https://www.who.int/influenza/human_animal_interface/2018_11_01_tableH5N1.pdf
9. World Health Organization. Antigenic and genetic characteristics of influenza A(H5N1) and influenza A(H9N2) viruses and candidate vaccine viruses developed for potential use in human vaccines - February 2010. *Wkly Epidemiol Rec*. Mar 12 2010;85(11):100-7.
10. Nguyen T, Rivailler P, Davis CT, et al. Evolution of highly pathogenic avian influenza (H5N1) virus populations in Vietnam between 2007 and 2010. *Virology*. Oct 25 2012;432(2):405-16. doi:10.1016/j.virol.2012.06.021
11. World Health Organization. Revised and updated nomenclature for highly pathogenic avian influenza A (H5N1) viruses. *Influenza Other Respir Viruses*. May 2014;8(3):384-8. doi:10.1111/irv.12230
12. Abdel-Ghafar AN, Chotpitayasunondh T, Gao Z, et al. Update on avian influenza A (H5N1) virus infection in humans. *N Engl J Med*. Jan 17 2008;358(3):261-73. doi:10.1056/NEJMra0707279
13. Iskander J, Strikas RA, Gensheimer KF, Cox NJ, Redd SC. Pandemic influenza planning, United States, 1978-2008. *Emerg Infect Dis*. Jun 2013;19(6):879-85. doi:10.3201/eid1906.121478
14. Oshansky CM, Zhou J, Gao Y, et al. Safety and immunogenicity of influenza A(H5N1) vaccine stored up to twelve years in the National Pre-Pandemic Influenza Vaccine Stockpile (NPIVS). *Vaccine*. 2018/12/12/ 2018;doi:https://doi.org/10.1016/j.vaccine.2018.11.069
15. Rotrosen ET, Neuzil KM. Influenza: A Global Perspective. *Pediatr Clin North Am*. Aug 2017;64(4):911-936. doi:10.1016/j.pcl.2017.03.007
16. Centers for Disease Control and Prevention. Past Seasons Vaccine Effectiveness Estimates. Accessed June 21, 2021, https://www.cdc.gov/flu/vaccines-work/past-seasons-estimates.html
17. Steel J. A paradigm shift in vaccine production for pandemic influenza. *Ann Transl Med*. Jul 2015;3(12):165. doi:10.3978/j.issn.2305-5839.2015.05.12
18. Pandey A, Singh N, Sambhara S, Mittal SK. Egg-independent vaccine strategies for highly pathogenic H5N1 influenza viruses. *Hum Vaccin*. Feb 2010;6(2):178-88.

19. Skowronski DM, De Serres G. Role of Egg-adaptation Mutations in Low Influenza A(H3N2) Vaccine Effectiveness During the 2012-2013 Season. *Clin Infect Dis*. Oct 15 2018;67(9):1474-1476. doi:10.1093/cid/ciy350
20. DiazGranados CA, Dunning AJ, Kimmel M, et al. Efficacy of high-dose versus standard-dose influenza vaccine in older adults. *N Engl J Med*. Aug 14 2014;371(7):635-45. doi:10.1056/NEJMoa1315727
21. Grohskopf LA, Sokolow LZ, Broder KR, Walter EB, Fry AM, Jernigan DB. Prevention and Control of Seasonal Influenza with Vaccines: Recommendations of the Advisory Committee on Immunization Practices-United States, 2018-19 Influenza Season. *MMWR Recomm Rep*. Aug 24 2018;67(3):1-20. doi:10.15585/mmwr.rr6703a1
22. Lee JKH, Lam GKL, Shin T, et al. Efficacy and effectiveness of high-dose versus standard-dose influenza vaccination for older adults: a systematic review and meta-analysis. *Expert Rev Vaccines*. May 2018;17(5):435-443. doi:10.1080/14760584.2018.1471989
23. Grohskopf LA, Sokolow LZ, Broder KR, et al. Prevention and Control of Seasonal Influenza with Vaccines: Recommendations of the Advisory Committee on Immunization Practices - United States, 2017-18 Influenza Season. *MMWR Recomm Rep*. Aug 25 2017;66(2):1-20. doi:10.15585/mmwr.rr6602a1
24. Parker L, Wharton SA, Martin SR, et al. Effects of egg-adaptation on receptor-binding and antigenic properties of recent influenza A (H3N2) vaccine viruses. *J Gen Virol*. Jun 2016;97(6):1333-44. doi:10.1099/jgv.0.000457
25. Luke CJ, Subbarao K. Improving pandemic H5N1 influenza vaccines by combining different vaccine platforms. *Expert Rev Vaccines*. Jul 2014;13(7):873-83. doi:10.1586/14760584.2014.922416
26. Food and Drug Administration. Guidance for Industry Clinical Data Needed to Support the Licensure of Pandemic Influenza Vaccines Accessed 6/21/21, https://www.fda.gov/downloads/BiologicsBloodVaccines/GuidanceComplianceRegulatoryInformation/Guidances/Vaccines/ucm091985.pdf
27. Mutsch M, Zhou W, Rhodes P, et al. Use of the inactivated intranasal influenza vaccine and the risk of Bell's palsy in Switzerland. *N Engl J Med*. Feb 26 2004;350(9):896-903. doi:10.1056/NEJMoa030595
28. World Health Organization. The immunological basis for immunization series: module 23: influenza vaccines. Accessed 6/21/21, https://apps.who.int/iris/rest/bitstreams/1090245/retrieve
29. Garcon N, Di Pasquale A. From discovery to licensure, the Adjuvant System story. *Hum Vaccin Immunother*. Jan 2 2017;13(1):19-33. doi:10.1080/21645515.2016.1225635
30. Food and Drug Administration. Guidance for Industry: Toxicity Grading Scale for Healthy Adult and Adolescent Volunteers Enrolled in Preventative Vaccine Clinical Trials. Accessed 6/21/21, https://www.fda.gov/regulatory-information/search-fda-guidance-documents/toxicity-grading-scale-healthy-adult-and-adolescent-volunteers-enrolled-preventive-vaccine-clinical

17 APPENDICES

Appendix A. Schedule of Events

Visit Number	1	2	3	4	5	6	7	8	9	10	11	12	13	14	
**Visit**	**Screen**	**Dose #1**	**Call[j]**	**Safety**	**Dose #2**	**Call[j]**	**Safety**	**Safety**	**Call[j]**	**Dose #3**	**Call[k]**	**Safety**	**Safety**	**Call[j]**	**Early Termination / Unsche-duled Visit[l]**
**Study Day[a]**	**-45 to -3**	**1**	**4**	**8**	**29**	**32**	**43**	**57**	**90**	**197**	**200**	**204**	**225**	**393**	
**Target Window (± days)[a]**	**NA**	**NA**	**± 2**	**± 2**	**± 4**	**± 2**	**± 3**	**± 4**	**± 7**	**± 7**	**± 1**	**± 2**	**± 7**	**+/-± 7**	
Informed Consent	X														
Incl/Excl Criteria	X	X			X					X					
Demographics	X														
Medical History	X	X													X
Prior/ Concomitant Medications	X	X	X	X	X	X	X	X	X	X	X	X	X	X	X
Height	X														
Weight	X	X			X					X					
Vital Signs[b]	X	X		X	X		X	X		X		X	X		X
Physical Examination[c]	X	X		X	X		X	X		X		X	X		X
Targeted neurologic/ otorhinoscopic/ respiratory examination[d]	X	X		X	X		X	X		X		X	X		X
Reactogenicity[e] Assessment		X		X	X		X			X		X			X
Drug Abuse Screening	X														
Serum Pregnancy	X														
Urine Pregnancy		X			X					X					
ESR and Screening Serology	X														
Chemistry, Hematology[g,h]	X			X	X		X			X		X			X
NP Swab Sample [i]		X			X										X
Serum Sample		X			X		X	X		X		X	X		X
PBMC Sample		X					X	X		X			X		X
Nasal Wash Sample	X			X			X	X		X		X	X		X
Saliva Sample	X			X			X	X		X		X	X		X
Nasal Matrix Sample	X			X			X	X		X			X		X

Visit Number	1	2	3	4	5	6	7	8	9	10	11	12	13	14	
Visit	Screen	Dose #1	Call[i]	Safety	Dose #2	Call[i]	Safety	Safety	Call[j]	Dose #3	Call[k]	Safety	Safety	Call[j]	Early Termination / Unsche-duled Visit[l]
**Study Day[a]**	**-45 to -3**	**1**	**4**	**8**	**29**	**32**	**43**	**57**	**90**	**197**	**200**	**204**	**225**	**393**	
**Target Window (± days)[a]**	**NA**	**NA**	**± 2**	**± 2**	**± 4**	**± 2**	**± 3**	**± 4**	**± 7**	**± 7**	**± 1**	**± 2**	**± 7**	**+/-± 7**	
Nasal Scraping Sample				X				X					X		X
Randomization		X													
Vaccination		X			X					X					
Symptom Diary		dispense	remind	collect	dispense	remind	collect			dispense	remind	collect			
Adverse Events		X	X	X	X	X	X	X	X	X	X	X	X		X
Serious Adverse Events		X	X	X	X	X	X	X	X	X	X	X	X	X	X
Subject Status-End of Study														X	

a. Days relative to vaccination are only estimates because the window allowance is not inclusive. Should a study pause occur, visits/windows will be adjusted to allow participants to continue without protocol deviation. Visit schedules following the vaccinations are calculated relative to the day the most recent vaccinations were received.

b. Obtain vitals prior to immunization and at 1-hour post-immunization during observation period (IN vaccine) or 30 minutes post-immunization (IM vaccine)

c. Physical exam is either 1) a baseline physical examination conducted during the screening visit to better characterize subject health and study eligibility, or 2) a directed physical examination targeting new complaints or symptoms to assess for changes since prior visit. If there are any clinically significant findings on physical exam performed after vaccination, these should be reported as an AE and repeat exam should be monitored until the AE is resolved. Refer to Section 7.1.1 for details.

d. The targeted neurologic/otorhinoscopic/respiratory examination will be of a limited nature as described in Appendix C. On IN vaccination days, the targeted neurologic/otorhinoscopic/respiratory examination will be performed prior to immunization and 1 hour following IN immunization at end of observation period on vaccination days If there are clinically significant findings on exam, these should be reported as an AE and repeat exam should be completed weekly until the AE is resolved.

e. Reactogenicity assessments will be conducted prior to immunization and 1 hour following IN immunization and 30 minutes following IM immunization. On non-immunization days, reactogenicity should be assessed following review of Symptom Diary with subject.

f. Serum screen for Hepatitis C Antibodies-EIA; Human Immunodeficiency Virus; HIV-1/HIV-2 ELISA or rapid test

g. Screening lab test set: white blood cell count, platelet count, hemoglobin, creatinine, alanine aminotransferase (ALT), total bilirubin, alkaline phosphatase

h. Safety lab test set: white blood cell count, platelet count, hemoglobin, creatinine, alanine aminotransferase (ALT), total bilirubin, alkaline phosphatase

i. Molecular diagnostic-based tests of nasopharyngeal swabs for SARS-CoV-2 will be performed within 2 days pre-vaccination, with a negative result required before vaccine can be received.

j. Symptom Diary is a tool created for the subject to record reactogenicity symptoms between leaving the clinic post-immunization through 7 days post – immunization. These serve as reminders to the subject what to discuss with the Investigator when the Investigator assesses reactogenicity symptoms at the next visit.

k. Safety Phone Call is scheduled 2-4 days post immunization to remind subject to complete Symptom Diary and to assess for potential AEs and SAEs beyond those listened on the Symptom Diary. Another Safety Phone Call is scheduled for Day 90 to assess for any new neurologic, otorhinoscopic, or respiratory symptoms requiring clinical evaluation. A final Safety Phone Call will be made at the study end.

l. Early Termination Visits or Unscheduled Visits may be conducted. In the event of the early termination of a subject, all attempts will be made to collect the assessments required at the current visit or at the next planned clinic visit. Subjects will be encouraged to continue safety assessments per the study schedule through the end of the trial. Unscheduled study visits will be scheduled for subjects to follow up on abnormal safety laboratory tests, medical complaints of concern for an AE, or to monitor documented AEs. Refer to Sections 6.3.13 and 6.4 for details.

Hematology – hemoglobin, white blood cell count, and platelet count.

Biochemistry – creatinine, total bilirubin, alanine aminotransferase (ALT), and alkaline phosphatase.

Immunology – nasal wash and blood samples will be collected, processed and stored for analyses of mucosal, humoral and cell-mediated immune responses.

Future Use – all saliva, nasal absorptive matrix, and some nasal wash and blood samples are collected for future use (as detailed in Appendix G).

Targeted Neurologic/Otorhinoscopic/Respiratory Exam: Will be performed to assess neurologic/ otorhinoscopic/respiratory functioning at screening, twice on the day of IN vaccination (before and 1 hour after vaccination), and during subsequent clinic visits.

Study Intervention – Study drug (BW-1014, active control or placebo) will be administered intranasally on Days 1 and 29 The boost vaccine of Influenza Virus Vaccine H5N1, will be administered by IM injection on Day 197.

Unless otherwise indicated, all procedures performed on Day 1/Day 29 /Day 197 should be completed prior to administering the study vaccine.

Appendix B. Toxicity Tables

CLINICAL ABNORMALITIES

Vital Signs *	Mild (Grade 1)	Moderate (Grade 2)	Severe (Grade 3)	Potentially Life Threatening (Grade 4)
Fever (°C) **<br>(°F) **	38.0 – 38.4<br>100.4 – 101.1	38.5 – 38.9<br>101.2 – 102.0	39.0 – 40<br>102.1 – 104	> 40<br>> 104
Tachycardia - beats per minute	101 – 115	116 – 130	> 130	ER visit or hospitalization for arrhythmia
Bradycardia - beats per minute***	50 – 54	45 – 49	< 45	ER visit or hospitalization for arrhythmia
Hypertension (systolic) - mm Hg	141 – 150	151 – 155	> 155	ER visit or hospitalization for malignant hypertension
Hypertension (diastolic) - mm Hg	91 – 95	96 – 100	> 100	ER visit or hospitalization for malignant hypertension
Hypotension (systolic) – mm Hg	85 – 89	80 – 84	< 80	ER visit or hospitalization for hypotensive shock
Respiratory Rate – breaths per minute	17 – 20	21 – 25	> 25	Intubation

* Subject should be at rest for all vital sign measurements.
** Oral temperature; no recent hot or cold beverages or smoking.
*** When resting heart rate is between 60 – 100 beats per minute. Use clinical judgement when characterizing bradycardia among some healthy subject populations, for example, conditioned athletes.

**Systemic (General)**	**Mild (Grade 1)**	**Moderate(Grade 2)**	**Severe (Grade 3)**	**Potentially Life Threatening (Grade 4)**
Nausea/vomiting	No interference with activity or 1 – 2 episodes/24 hours	Some interference with activity or > 2 episodes/24 hours	Prevents daily activity, requires outpatient IV hydration	ER visit or hospitalization for hypotensive shock
Diarrhea	2 – 3 loose stools or < 400 gms/24 hours	4 – 5 stools or 400 – 800 gms/24 hours	6 or more watery stools or > 800gms/24 hours or requires outpatient IV hydration	ER visit or hospitalization
Headache	No interference with activity	Repeated use of non-narcotic pain reliever > 24 hours or some interference with activity	Significant; any use of narcotic pain reliever or prevents daily activity	ER visit or hospitalization
Fatigue	No interference with activity	Some interference with activity	Significant; prevents daily activity	ER visit or hospitalization
Myalgia	No interference with activity	Some interference with activity	Significant; prevents daily activity	ER visit or hospitalization
**Systemic Illness**	**Mild (Grade 1)**	**(Moderate (Grade 2)**	**Severe (Grade 3)**	**Potentially Life Threatening (Grade 4)**
Illness or clinical adverse event (as defined according to applicable regulations)	No interference with activity	Some interference with activity not requiring medical intervention	Prevents daily activity and requires medical intervention	ER visit or hospitalization

LABORATORY ABNORMALITIES

The following tables will be used in the assessment of study laboratory abnormalities. The laboratory values provided in the tables below serve as guidelines and are dependent upon institutional normal parameters. Institutional normal reference ranges should be provided to demonstrate that they are appropriate.

Chemistry

**Serum ***	**Mild (Grade 1)**	**Moderate (Grade 2)**	**Severe (Grade 3)**	**Potentially Life Threatening (Grade 4)****
Creatinine – mg/dL	1.4 – 1.7 (male)<br>1.3 – 1.7 (female)	1.8 – 2.0	2.1 – 2.5	> 2.5 or requires dialysis
Alkaline phosphate – increase by factor	1.1 – 2.0 x ULN	2.1 – 3.0 x ULN	3.1 – 10 x ULN	> 10 x ULN
alanine aminotransferase (ALT) increase by factor	1.1 – 2.5 x ULN	2.6 – 5.0 x ULN	5.1 – 10 x ULN	> 10 x ULN
Bilirubin – when accompanied by any increase in ALT increase by factor	1.1 – 1.25 x ULN	1.26 – 1.5 x ULN	1.51 – 1.75 x ULN	> 1.75 x ULN
Bilirubin – when ALT is normal; increase by factor	1.1 – 1.5 x ULN	1.6 – 2.0 x ULN	2.0 – 3.0 x ULN	> 3.0 x ULN

* The laboratory values provided in the tables serve as guidelines and are dependent upon institutional normal parameters. Institutional normal reference ranges should be provided to demonstrate that they are appropriate.

** The clinical signs or symptoms associated with laboratory abnormalities might result in characterization of the laboratory abnormalities as Potentially Life Threatening (Grade 4). For example. a low sodium value that falls within a grade 3 parameter (125-129 mE/L) should be recorded as a grade 4 hyponatremia event if the subject had a new seizure associated with the low sodium value.

***"ULN" is the upper limit of the normal range.

Hematology

Hematology *	Mild (Grade 1)	Moderate (Grade 2)	Severe (Grade 3)	Potentially Life Threatening (Grade 4)
Hemoglobin (Female) - gm/dL	11.0 – 12.0	9.5 – 10.9	8.0 – 9.4	< 8.0
Hemoglobin (Female) change from baseline value - gm/dL	Any decrease – 1.5	1.6 – 2.0	2.1 – 5.0	> 5.0
Hemoglobin (Male) - gm/dL	12.5 – 13.5	10.5 – 12.4	8.5 – 10.4	< 8.5
Hemoglobin (Male) change from baseline value – gm/dL	Any decrease – 1.5	1.6 – 2.0	2.1 – 5.0	> 5.0
WBC Increase - cell/mm^3	11,001 – 15,000	15,001 – 20,000	20,001 – 25, 000	> 25,000
WBC Decrease - cell/mm^3	2,500 – 3,500	1,500 – 2,499	1,000 – 1,499	< 1,000
Platelets Decreased - cell/mm^3	125,000 – 140,000	100,000 – 124,000	25,000 – 99,000	< 25,000

* The laboratory values provided in the tables serve as guidelines and are dependent upon institutional normal parameters. Institutional normal reference ranges should be provided to demonstrate that they are appropriate.

** "ULN" is the upper limit of the normal range.

Appendix C. Targeted Neurologic/Otorhinoscopic/Respiratory Assessments

The assessments to be made as part of the targeted neurologic/otorhinoscopic/respiratory examination are provided below.

Grading Scale:

- Grade 0 = Not present.
- Grade 1 = Present but no interference with usual activities.
- Grade 2 = Present strongly enough to prevent a significant part of usual activities.
- Grade 3 = Present and prevented most or all of normal activities or warranted medical attention/prescription medicine.
- Grade 4 = Potentially life-threatening, requires ER visit or hospitalization.

Eyes

- Tearing or Discharge □ Present □ Absent
- Periorbital Swelling □ Present □ Absent
- Extraocular movements □ Normal □Abnormal

Ears

- Erythema □ Present □ Absent
- Discharge □ Present □ Absent
- Ear Drum □ Normal □ Abnormal
- Ear Canal □ Normal □ Abnormal

Nose

- External
  - Erythema □ Present □ Absent
  - Swelling □ Present □ Absent
  - Discharge □ Present □ Absent
  - Crusting □ Present □ Absent
- Mucosa
  - Color □ Normal □ Pale □ Erythematous
  - Texture □ Smooth □ Boggy
  - Swelling □ Present □ Absent
- Secretions
  - Color □ Clear □ Discolored □ Bloody
  - Consistency □ Thick □ Watery
  - Amount □ Profuse □ Moderate □ Minimal
- Polyps □ Present □ Absent
- Lesions □ Present □ Absent
- Obstruction □ Present □ Absent
- Septum □ Non-Deviated □ Deviated □ Obstructed

Mouth/Throat

- Lips Crusting □ Cracking □ Lesions □ Normal

- Mucosa
  - Color □ Normal □ Pale □ Erythematous
  - Texture □ Smooth □ Boggy
- Secretions
  - Color □ Clear □ Discolored □ Bloody
  - Consistency □ Thick □ Watery
  - Amount □ Profuse □ Moderate □ Minimal
- Obstruction □ Present □ Absent
- Lesions □ Present □ Absent
- Swallowing □ Normal □ Difficult

Face/Neck

- Eyebrow Lift □ Normal □ Abnormal
- Strength of Eye Lid Closure □ Normal □ Abnormal
- Smile □ Normal □ Abnormal
- Jaw Strength □ Normal □ Abnormal
- Facial Drooping □ Present □ Absent
- Pain/tenderness □ Present □ Absent
- Swelling □ Present □ Absent
- Lumps/Lymph nodes □ Present □ Absent

Respiratory

- Wheezing □ Present □ Absent
- Rales □ Present □ Absent
- Rhonchi □ Present □ Absent

Extremities

- Joint Swelling □ Present □ Absent
- Joint Pain □ Present □ Absent

Appendix D. Reactogenicity Assessments (post-IN vaccination)

Reactogenicity Assessments for BW-1014: The following grading scale and list of signs and symptoms will be used by investigators to rate reactogenicity at one hour following each IN immunization and at the clinic visit one week following each immunization. Subjects will be provided a Symptom Diary containing similar information to record symptoms daily during the intervening week for discussion with the investigator at their clinic visit one week after immunization.

<u>Grading Scale (over the last 24 hours):</u>

- Grade 0 = Not present.
- Grade 1 = Present but no interference with usual activities.
- Grade 2 = Present strongly enough to prevent a significant part of usual activities.
- Grade 3 = Present and prevented most or all of normal activities or warranted medical attention/prescription medicine.
- Grade 4 = Potentially life-threatening, requires ER visit or hospitalization.

<u>Signs and Symptoms of Reactogenicity to be Evaluated</u>

Fever >100.4 (measured as present/absent only) / Feverishness<br>Joint pain<br>Body aches/ Muscular pain<br>Headache<br>Tiredness<br>Nausea<br>Watery eyes<br>Itchy eyes<br>Red eyes<br>Blurry vision<br>Double Vision<br>Swelling around the eyes<br>Sneezing<br>Runny nose<br>Postnasal drip<br>Stuffy nose<br>Itchy nose<br>Inability to smell<br>Bleeding from the nose<br>Coughing<br>Difficulty with hearing<br>Ringing in ears	Tightness in the chest<br>Wheezing<br>Trouble swallowing<br>Hoarse voice<br>Sore throat<br>Slurred speech<br>Dizziness<br>Difficulty sleeping<br>Food tasting strange<br>Decrease in your appetite

In addition, subjects will be asked to record any other symptoms experienced since their last visit on the Symptom Diary.

Appendix E. Reactogenicity Assessment (post-IM injection)

Reactogenicity Assessments for H5N1 IIV: The following grading scales for signs and symptoms will be used by investigators to rate reactogenicity at 30 minutes following the IM immunization and at the clinic visit one week following each immunization.

**Local Reaction to Injectable Product**	**Mild (Grade 1)**	**Moderate (Grade 2)**	**Severe (Grade 3)**	**Potentially Life Threatening (Grade 4)**
Pain	Does not interfere with activity	Repeated use of non- narcotic pain reliever > 24 hours or interferes with activity	Any use of narcotic pain reliever or prevents daily activity	Emergency room (ER) visit or hospitalization
Tenderness	Mild discomfort to touch	Discomfort with movement	Significant discomfort at rest	ER visit or hospitalization
Erythema/Redness *	2.5 – 5 cm	5.1 – 10 cm	> 10 cm	Necrosis or exfoliative dermatitis
Induration/Swelling **	2.5 – 5 cm and does not interfere with activity	5.1 – 10 cm or interferes with activity	> 10 cm or prevents daily activity	Necrosis

* In addition to grading the measured local reaction at the greatest single diameter, the measurement should be recorded as a continuous variable.

** Induration/Swelling will be evaluated and graded using the functional scale as well as the actual measurement.

Vital signs assessed will include temperature, heart rate, blood pressure, and respiratory rate. Systemic signs and symptoms will include nausea/vomiting, diarrhea, headache, fatigue, myalgia, and illness or clinical adverse events. Reactogenicity assessment will be done according to relevant Toxicity Table in Appendix B.

Subjects will be provided a Symptom Diary containing similar information to record symptoms daily during the intervening week for discussion with the investigator at their clinic visit one week after immunization. In addition, subjects will be asked to record any other symptoms experienced since their last visit on the Symptom Diary.

Appendix F. Potentially Immune-Mediated Medical Conditions (PIMMCs)

Gastrointestinal Disorders

- Celiac disease
- Crohn's disease
- Ulcerative colitis
- Ulcerative proctitis

Liver Disorders

- Autoimmune cholangitis
- Autoimmune hepatitis
- Primary biliary cirrhosis
- Primary sclerosing cholangitis

Metabolic Diseases

- Addison's disease
- Autoimmune thyroiditis (including Hashimoto thyroiditis)
- Diabetes mellitus type I
- Grave's or Basedow's disease

Musculoskeletal Disorders

- Antisynthetase syndrome
- Dermatomyositis
- Juvenile chronic arthritis (including Still's disease)
- Mixed connective tissue disorder
- Polymyalgia rheumatic
- Polymyositis
- Psoriatic arthropathy
- Relapsing polychondritis
- Rheumatoid arthritis
- Scleroderma, including diffuse systemic form and CREST syndrome
- Spondyloarthritis, including ankylosing spondylitis, reactive arthritis (Reiter's Syndrome) and undifferentiated spondyloarthritis
- Systemic lupus erythematosus
- Systemic sclerosis

Neuroinflammatory Disorders

- Acute disseminated encephalomyelitis, including site specific variants (e.g., non-infectious encephalitis, encephalomyelitis, myelitis, myeloradiculomyelitis)
- Cranial nerve disorders, including paralyses/paresis (e.g., Bell's palsy)
- Guillain-Barré syndrome, including Miller Fisher syndrome and other variants

- Immune-mediated peripheral neuropathies and plexopathies, including chronic inflammatory demyelinating polyneuropathy, multifocal motor neuropathy and polyneuropathies associated with monoclonal gammopathy
- Multiple sclerosis
- Narcolepsy
- Optic neuritis
- Transverse myelitis
- Myasthenia gravis, including Eaton-Lambert syndrome

Skin Disorders

- Alopecia areata
- Autoimmune bullous skin diseases, including pemphigus, pemphigoid and dermatitis herpetiformis
- Cutaneous lupus erythematosus
- Erythema nodosum
- Morphea
- Lichen planus
- Psoriasis
- Sweet’s syndrome
- Vitiligo

Vasculitides

- Large vessels vasculitis including: giant cell arteritis such as Takayasu's arteritis and temporal arteritis
- Medium sized and/or small vessels vasculitis including: polyarteritis nodosa, Kawasaki's disease, microscopic polyangiitis, Wegener's granulomatosis, Churg–Strauss syndrome (allergic granulomatous angiitis), Buerger’s disease thromboangiitis obliterans, necrotizing vasculitis and anti-neutrophil cytoplasmic antibody (ANCA) positive vasculitis (type unspecified), Henoch- Schonlein purpura, Behcet's syndrome, leukocytoclastic vasculitis

Others

- Antiphospholipid syndrome
- Autoimmune hemolytic anemia
- Autoimmune glomerulonephritis (including IgA nephropathy, glomerulonephritis rapidly progressive, membranous glomerulonephritis, membranoproliferative glomerulonephritis, and mesangioproliferative glomerulonephritis)
- Autoimmune myocarditis/cardiomyopathy
- Autoimmune thrombocytopenia
- Goodpasture syndrome
- Idiopathic pulmonary fibrosis
- Pernicious anemia
- Raynaud’s phenomenon

- Sarcoidosis
- Sjögren’s syndrome
- Stevens-Johnson syndrome
- Uveitis

Appendix G. Procedure Volumes

**Visit Number**	1	2	3	4	5	6	7	8	9	10	11	12	13	14	
**Visit**	**Screen**	**Dose #1**	**Call**[i]	**Safety**	**Dose #2**	**Call**[i]	**Safety**	**Safety**	**Call**[i]	**Dose #3**	**Call**[j]	**Safety**	**Safety**	**Call**[j]	**Early Termination/ Unsche-duled Visit**[d]
**Study Day**	**-45 to -3**	**1**	**4**	**8**	**29**	**32**	**43**	**57**	**90**	**197**	**200**	**204**	**225**	**393**	
**Target Window (± days)**	**NA**	**NA**	**± 2**	**± 2**	**± 4**	**± 2**	**± 2**	**± 4**	**± 7**	**± 7**	**± 2**	**± 2**	**± 7**	**± 7**	
Urine Pregnancy		collect			collect					collect					
ESR, Screening Serology, Pregnancy	8.5 mL														
Chemistry, Hematology	12.5 mL			12.5 mL	12.5 mL		12.5 mL			12.5 mL		12.5 mL			12.5 mL
NP Swab Sample		collect			collect										collect
Serum Sample		17 mL			17 mL		17 mL	17 mL		17 mL		17 mL	17 mL		17 mL
PBMC Sample		70 mL					70 mL[b]	70 mL		70 mL			70 mL		70 mL
Nasal Wash Sample	3-7 mL			3-7 mL			3-7 mL[c]	3-7 mL[c]		3-7 mL		3-7 mL	3-7 mL		3-7 mL
Saliva Sample	2 mL[b]			2 mL[b]			2 mL[b]	2 mL[b]		2 mL[b]		2 mL[b]	2 mL[b]		2 mL[b]
Nasal Absorptive Matrix Sample	collect[b]			collect[b]			collect[b]	collect[b]		collect[b]			collect[b]		collect[b]
Nasal Mucosal Scraping Sample				collect[c]				collect[c]					collect[c]		collect[c]

a. Estimated total volume blood collected is 552.5mL over the study period.
b. Collected for future use only.
c. Cell isolation to be done from these nasal specimens.
d. May include specimen collection, as detailed in Sections 6.3.13.
