# Supplementary material for: An intranasal adjuvanted, recombinant influenza A/H5 vaccine primes against diverse H5N1 clades: a phase I trial
Source: Nat Commun. 2025 Nov 6;16:9321. doi: 10.1038/s41467-025-64686-3 (PMC12592354; doi:10.1038/s41467-025-64686-3)
Supplement: Supplementary file 2 — Reporting Summary [file 41467_2025_64686_MOESM2_ESM.pdf]

nature portfolio

Corresponding author(s): Justin R. Ortiz

Last updated by author(s): Feb 22, 2025

Reporting Summary

Nature Portfolio wishes to improve the reproducibility of the work that we publish. This form provides structure for consistency and transparency in reporting. For further information on Nature Portfolio policies, see our Editorial Policies and the Editorial Policy Checklist.

Statistics

For all statistical analyses, confirm that the following items are present in the figure legend, table legend, main text, or Methods section.

n/a	Confirmed	
☐	☒	The exact sample size (*n*) for each experimental group/condition, given as a discrete number and unit of measurement
☐	☒	A statement on whether measurements were taken from distinct samples or whether the same sample was measured repeatedly
☐	☒	The statistical test(s) used AND whether they are one- or two-sided<br>*Only common tests should be described solely by name; describe more complex techniques in the Methods section.*
☒	☐	A description of all covariates tested
☐	☒	A description of any assumptions or corrections, such as tests of normality and adjustment for multiple comparisons
☐	☒	A full description of the statistical parameters including central tendency (e.g. means) or other basic estimates (e.g. regression coefficient) AND variation (e.g. standard deviation) or associated estimates of uncertainty (e.g. confidence intervals)
☐	☒	For null hypothesis testing, the test statistic (e.g. *F*, *t*, *r*) with confidence intervals, effect sizes, degrees of freedom and *P* value noted<br>*Give P values as exact values whenever suitable.*
☒	☐	For Bayesian analysis, information on the choice of priors and Markov chain Monte Carlo settings
☒	☐	For hierarchical and complex designs, identification of the appropriate level for tests and full reporting of outcomes
☒	☐	Estimates of effect sizes (e.g. Cohen's *d*, Pearson's *r*), indicating how they were calculated

Our web collection on statistics for biologists contains articles on many of the points above.

Software and code

Policy information about availability of computer code

Data collection	We have included a description of the data collection and management software used for the trial (REDCap) with citations..
Data analysis	We have included a description of the statistical analysis software (Stata and SAS) and software to make the figures (Microsoft Office software, GraphPad Prism, and JMP.

For manuscripts utilizing custom algorithms or software that are central to the research but not yet described in published literature, software must be made available to editors and reviewers. We strongly encourage code deposition in a community repository (e.g. GitHub). See the Nature Portfolio guidelines for submitting code & software for further information.

Data

Policy information about availability of data

All manuscripts must include a data availability statement. This statement should provide the following information, where applicable:

- Accession codes, unique identifiers, or web links for publicly available datasets
- A description of any restrictions on data availability
- For clinical datasets or third party data, please ensure that the statement adheres to our policy

Data availability statement is after the Acknowledgment section.

Research involving human participants, their data, or biological material

Policy information about studies with human participants or human data. See also policy information about sex, gender (identity/presentation), and sexual orientation and race, ethnicity and racism.

Reporting on sex and gender	No sex or gender-based analyses were performed. We described how sex was defined and collected in a note after Online Display 1.
Reporting on race, ethnicity, or other socially relevant groupings	No race, ethnicity, or other socially relevant groupings-based analyses were performed. We describe how race and ethnicity were defined and collected in a note after Online Display 1.
Population characteristics	No analyses were performed by population characteristics. Age, sex, ethnicity, race, and body mass index are summarized by study group in Online Display 1.
Recruitment	Recruitment is described in the methods: "Participants were recruited from a volunteer database at the clinical site and through advertisements in the Baltimore/Washington, DC, region"
Ethics oversight	This is reported in a "regulatory and ethics" subsection at the end of the methods section: The protocol and informed consent forms were reviewed and approved by the University of Maryland, Baltimore Institutional Review Board and the study was registered at Clinicaltrials.gov (NCT05397119).

Note that full information on the approval of the study protocol must also be provided in the manuscript.

Field-specific reporting

Please select the one below that is the best fit for your research. If you are not sure, read the appropriate sections before making your selection.

☒ Life sciences ☐ Behavioural & social sciences ☐ Ecological, evolutionary & environmental sciences

For a reference copy of the document with all sections, see nature.com/documents/nr-reporting-summary-flat.pdf

Life sciences study design

All studies must disclose on these points even when the disclosure is negative.

Sample size	There were no pre-specified hypothesis tests for this phase I trial. The sample size of 40 with 8 persons per vaccine group was chosen without the intention that group differences would be detected with a sufficient power but was consistent with FDA guidance.
Data exclusions	The analysis population includes all participants who received study vaccination and for whom data were available at any particular timepoint.
Replication	Flow cytometry: Experiments were run groups of 3-5 volunteers (all time points from the same volunteers were run together). As PBMC from human trials are not always available for repeats, an internal control was used across all experiments from a specific project. The internal control consisted of PBMC from a volunteer with known responses to diverse stimuli. The samples from the internal control were stimulated with Media and SEB (Negative and Positive Controls). The percentages of the main T cells populations (CD4 and CD8) as well as one cytokine (IFN-gamma) in memory CD4 T cells were tracked in the internal control volunteer after each experiment. Percentages of CD4 and CD8 T cells were required to be +/- 5% of the initial experiment. The percentage of IFN-gamma positive cells in SEB stimulations with CD4+ CD69+ cells was required to be within +/- 1.5% of the initial experiment. If the percentages were out of range, all samples from the experiment were repeated (if samples available). Additionally, once all data was collected, CD4, CD8, as well as IFN-gamma, IL-2 and TNF-alpha in CD4 Memory cells of the internal control volunteer from all experiments were analyzed to ensure the percentages of these populations were within the Mean +/- 2SD. All experiments were performed by the same personnel. Monoclonal antibodies were from the same lot.<br>ADCC: All experiments were run in triplicate wells. Data from the triplicate wells was required to have a CV <25%. If necessary the samples were assessed again. Additionally, a universal positive and negative controls were included in each experiment. During optimization of the method a fold-change range was established for the the positive control (Mean +/- 2SD : 94 +/- 29.8 fold-change). All experiments were performed by the same personnel.<br>Memory B cell assays: All experiments were run in quadruplicate wells. The average of the 4 replicas was reported. Spots within the replicas were required to be +/- 2-fold from the Median of the 4 replicas. If out of range (outlier wells), the data from these wells was not considered for the final report. If the wells were over saturated and the spots could not be counted (too many spots to count), the experiment was repeated using 2-fold dilutions of the number of cells initially plated in each well. All experiments were performed by the same personnel.
Randomization	Eligible participants were randomized using block randomization.
Blinding	For the intranasal vaccinations, participants and study staff involved in post-vaccination assessments were blinded to vaccine allocation. However, due to differences in the appearance of rH5-NE compared to unadjuvanted or placebo formulations, the study staff administering the vaccines were unmasked.

Reporting for specific materials, systems and methods

We require information from authors about some types of materials, experimental systems and methods used in many studies. Here, indicate whether each material, system or method listed is relevant to your study. If you are not sure if a list item applies to your research, read the appropriate section before selecting a response.

Materials & experimental systems

n/a	Involved in the study	
☐	☒	Antibodies
☐	☒	Eukaryotic cell lines
☒	☐	Palaeontology and archaeology
☒	☐	Animals and other organisms
☐	☒	Clinical data
☒	☐	Dual use research of concern
☒	☐	Plants

Methods

n/a	Involved in the study	
☒	☐	ChIP-seq
☐	☒	Flow cytometry
☒	☐	MRI-based neuroimaging

Antibodies

Antibodies used	See Methods section and Supplemental Table 7 for descriptions of antibodies used.
Validation	In flow cytometry experiments no primary antibodies were used. In these experiments we used monoclonal antibodies derived from mice or rat (IL-2). All the monoclonal antibodies selected were tested for the target by the supplier. Please see corresponding web sites and catalog numbers for detailed descriptions

Eukaryotic cell lines

Policy information about cell lines and Sex and Gender in Research

Cell line source(s)	PBMC (primary cells) were collected from participants enrolled in the study. For ADCC assessments, a recombinant Jurkat T cell expressing the firefly luciferase gene under the control of the NFAT response elements with constitutive expression of human FcγRIIIa (V158) was acquired from BPS Bioscience.
Authentication	None of the cell lines were authenticated
Mycoplasma contamination	The cell line for ADCC assays was screened for mycoplasma using a PCR-based Venor GeM Mycoplasma Detection kit (Sigma-Aldrich).
Commonly misidentified lines (See ICLAC register)	N/A

Clinical data

Policy information about clinical studies

All manuscripts should comply with the ICMJE guidelines for publication of clinical research and a completed CONSORT checklist must be included with all submissions.

Clinical trial registration	Clinical trials registration number is in the Regulatory and Ethics subsection of the Methods Section and in the study abstract.
Study protocol	The full study protocol has been submitted to Clinicaltrials.gov, but it has not yet been published as we work through some data formatting queries from the website.
Data collection	This is described in the Methods section: We conducted this study at the University of Maryland School of Medicine Center for Vaccine Development and Global Health (Baltimore, MD, USA).
Outcomes	These are completely described in the Methods section.

Plants

Seed stocks	*Report on the source of all seed stocks or other plant material used. If applicable, state the seed stock centre and catalogue number. If plant specimens were collected from the field, describe the collection location, date and sampling procedures.*
Novel plant genotypes	*Describe the methods by which all novel plant genotypes were produced. This includes those generated by transgenic approaches, gene editing, chemical/radiation-based mutagenesis and hybridization. For transgenic lines, describe the transformation method, the number of independent lines analyzed and the generation upon which experiments were performed. For gene-edited lines, describe the editor used, the endogenous sequence targeted for editing, the targeting guide RNA sequence (if applicable) and how the editor was applied.*
Authentication	*Describe any authentication procedures for each seed stock used or novel genotype generated. Describe any experiments used to assess the effect of a mutation and, where applicable, how potential secondary effects (e.g. second site T-DNA insertions, mosiacism, off-target gene editing) were examined.*

Flow Cytometry

Plots

Confirm that:

- [x] The axis labels state the marker and fluorochrome used (e.g. CD4-FITC).
- [x] The axis scales are clearly visible. Include numbers along axes only for bottom left plot of group (a 'group' is an analysis of identical markers).
- [x] All plots are contour plots with outliers or pseudocolor plots.
- [x] A numerical value for number of cells or percentage (with statistics) is provided.

Methodology

Sample preparation	Please see Materials and Methods section.<br>Cryopreserved PBMC were thawed and rested for 4 hours (37°C, 5% CO2). PBMC were then washed and partitioned into four 2x10e6 cell aliquots. Two aliquots were stimulated with 1) rH5 from A/Indonesia/5/2005 (clade 2.1) (Fraunhofer USA Center for Molecular Biotechnology) at 3 μg/mL; and 2) a H5 HA peptide pool from A/Vietnam/1203/2004 (clade 1) at 2 μg/mL of each peptide. The peptide pool consisted of a 93-peptide array (12- or 17-mers, with 11aa overlap) from BEI Bioresources (NR-18974). The other two aliquots served as negative (media) and positive (Staphylococcal enterotoxin B -SEB-; 10 mg/mL) controls. All samples received anti-CD107a-FITC (clone H4A3; Becton Dickinson Biosciences -BDB-) and anti-CD28/CD49d co-stimulatory antibodies (BDB, USA). Samples were maintained at 37°C, 5% CO2. Two hours later, Brefeldin A and Monensin were added and incubated overnight (16 hours, at 37°C, 5% CO2). Next day the cells were stained for flow-cytometry. Briefly, cells were stained for viability (fixable yellow staining dye; 20 min; RT) and then with a surface antibody cocktail (30 min, Room temperature -RT-) that included: CD62L-PE , CD4-PerCP-Cy5.5, CD19-BV570, CD56-BV570, CD3-BV650, CD8-A700, CD45RA-APC-H7 (See Supplementary Table 1 for a list of the antibodies and lot numbers). The cells were then fixed, permeabilized and stained with an intracellular cocktail (30 min, RT) including the next antibodies: CD69-ECD, IFN-γ-PE-Cy7, IL-17A-BV605, TNF-α-BV711, CD154-BV785, IL-2-APC, and CD137-BV421 (see Supplementary Table 1 for further details). Cells were then fixed (1% PFA) and the samples collected in a custom LSRII flow cytometer (BD, USA). FCS files were analyzed using FlowJo (Tree Star, San Francisco, USA). Multifunctional analyses were performed using the Boolean gating function of FlowJo.
Instrument	BD LSR-2
Software	Software in cytometer: BD FACSDiva v.8.0.1.<br>Software for data analysis: FlowJo v10.10.0
Cell population abundance	No sorting was performed
Gating strategy	The lymphocyte population was identified in FSC/SSC biexponential plots. In this step, small events (debris) and large cells were eliminated. Subsequently, doublets were eliminated in FSC-H/FSC-A biexponential plots. Alive CD3+ cells were subsequently selected (Yevid- CD19- CD56-). Within CD3+ T cells, CD4+ and CD8+ T cells were selected. Within CD8 T cells, memory cells were identified using CD45RA and CD62L bi-exponential plots. CD8 memory T cells excluded double positive CD45RA/CD62L cells. A similar approach was used to identify CD4 memory T cells (See Supplementary Figure 1 for an example of the gating strategy used in a representative volunteer).<br>Cytokines were gated in CD69+ CD4 Memory or CD8 Memory T cells. Similarly, up-regulation of CD107a, CD137 and CD154 (in CD4 only) were assessed in CD69+ cells. Gates to determine positive and negative populations were based on the Media and SEB controls (Negative and Positive, respectively). Supplementary Figures 2 and 3 show representative plots of the gating approach used to identify cytokine/expression of activation markers in CD4 Memory and CD8 Memory T cells, respectively. Note: setting of the gates was based on positive and negative responses.

- [x] Tick this box to confirm that a figure exemplifying the gating strategy is provided in the Supplementary Information.
